# Thioester-containing protein TEP15 promotes malaria parasite development in mosquitoes through negative regulation of melanization

**DOI:** 10.1186/s13071-025-06772-5

**Published:** 2025-04-01

**Authors:** Xin Qin, Jianyong Li, Feng Zhu, Jian Zhang

**Affiliations:** 1 Department of Pathogenic Biology, Army Medical University (Third Military Medical University), Chongqing, 400038 China; 2 Key Laboratory of Extreme Environmental Medicine, Ministry of Education of China, Chongqing, 400038 China

**Keywords:** *Anophelesstephensi*, Thioester-containing protein, *Plasmodium*, AsTEP15, Melanization

## Abstract

**Background:**

Thioester-containing proteins (TEPs) serve as crucial effectors and regulatory components within the innate immune system of mosquitoes. Despite their significance, the mechanisms by which TEPs exert negative regulation on the immune response in mosquitoes remain inadequately understood. This study aims to elucidate the role of TEPs in the negative regulation of melanization, thereby advancing our comprehension of their regulatory function in the immune response.

**Methods:**

We infected female *Anopheles stephensi* mosquitoes with *Plasmodium yoelii* by allowing them to feed on pre-infected female Kunming mice. Western blot, quantitative polymerase chain reaction, differential gene expression analyses, and gene silencing were then conducted. Student’s *t*-test was used to analyze continuous variables, with statistical significance defined as* p* < 0.05.

**Results:**

*A. stephensi* TEP15 (AsTEP15) negatively regulated mosquitos’ innate immunity and promoted *Plasmodium* development. AsTEP15 knockdown induced mosquito resistance to malaria parasite melanization during the oocyst stage and significantly reduced sporozoite numbers. Further analysis showed that AsTEP15 mainly negatively affects the TEP1 and immune deficiency (IMD) pathway, thereby inhibiting melanization.

**Conclusions:**

We describe a mosquito TEP that negatively regulates immunity, further enriching the functional diversity of TEP family members. In addition, our results suggest that oocysts may exploit TEPs to escape or inhibit mosquito immunity, highlighting potential targets for blocking malaria transmission.

**Graphical Abstract:**

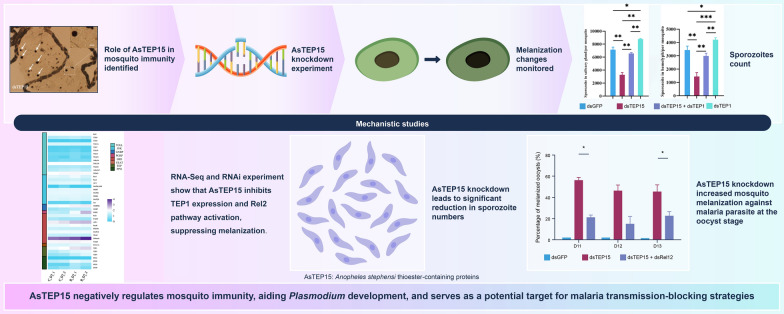

**Supplementary Information:**

The online version contains supplementary material available at 10.1186/s13071-025-06772-5.

## Background

Malaria is one of the most severe infectious diseases. In 2022, the worldwide incidence of malaria was estimated to be 249 million cases across 85 endemic countries and regions, marking an increase of 5 million cases compared with 2021[1]. *Plasmodium* transmission begins when a female *Anopheles* mosquito feeds on an infected host’s blood. Inside the mosquito, the parasite develops in the midgut: gametocytes transform into gametes, fertilize to form zygotes within 16–20 h, and become ookinetes. These ookinetes cross the midgut epithelium, forming oocysts on the basal side. The oocysts produce sporozoites, which enter the mosquito’s hemocoel and invade the salivary glands.

Mosquitoes have evolved a sophisticated innate immune system to counteract the proliferation of *Plasmodium*. Thioester-containing proteins (TEPs) play a pivotal role in the innate immune responses of arthropods [2–6]. In vertebrates, the TEP family encompasses α2-macroglobulins (A2Ms), CD109, and complement factors, all of which are integral to pathogen recognition and elimination [7–9]. A2Ms are primarily recognized as broad-spectrum protease inhibitors that sequester and deactivate microbial enzymes, which could pose a threat in the contexts of inflammation, immunity, and infection [7]. CD109 is characterized by its glycosylphosphatidylinositol (GPI) anchor and its substantial similarity to A2M. Its thioester domain (TED) indicates a potential for binding proteins that possess foreign carbohydrates or amines [8]. Furthermore, CD109 functions as a co-receptor for the isoforms of transforming growth factor-beta (TGF-β), thereby inhibiting TGF-β signaling [10].

Research on invertebrate thioester-containing proteins (TEPs) has predominantly concentrated on mosquitoes and *Drosophila melanogaster* [11–13]. The genome of *Anopheles gambiae* encodes 19 homologs of invertebrate TEPs (iTEPs). Among these, *A. gambiae* TEP1 (AgTEP1) is the most extensively studied, distinguished by its unique crystallized structure[14], and functions as an opsonin during the phagocytosis of both gram-negative and gram-positive bacteria [15–17]. AgTEP1 also binds to the surface of *Plasmodium*, facilitating melanization, which is crucial for reducing the number of *Plasmodium* ookinetes in mosquitoes [18, 19]. Our previous research demonstrated that *Anopheles stephensi* TEP1 (AsTEP1) plays a role in oocyst melanization [20]. Additionally, the knockdown of AgTEP1, AgTEP3, and AgTEP4 resulted in a decreased number of periostial hemocytes and reduced bacterial and melanin deposits following *E. coli* infection, without impacting non-periostial hemocytes. This suggests that these proteins are involved in the regulation of immune and circulatory system integration in mosquitoes [21]. In *Aedes aegypti*, silencing TEP1 led to an increase in viral RNA [5]. AgTEP15 was identified within the undirected gene co-expression network that includes *A. gambiae* genes from families known to facilitate melanization [22]. In addition, the silencing of *Wolbachia*-infection-responsive immune genes, specifically TEP4 and TEP15, in *A. stephensi* led to a significantly increased susceptibility to *Plasmodium falciparum* infection [23]. The *Drosophila* TEP family comprises six genes (TEP1–TEP6), with TEP5 appearing to be unexpressed [24–26]. DmTEP2, DmTEP3, and DmTEP6 function as opsonins, binding to fungi and both gram-negative and gram-positive bacteria to enhance phagocytosis [25]. Consequently, TEPs demonstrate functional diversity and play critical roles in the invertebrate immune response. Understanding their diverse regulatory mechanisms and mediated immune effects is essential for studying pathogen–invertebrate interactions.

Melanization is an effective antiparasitic and antimicrobial response in insects [27]. Phenoloxidase (PO) is a key enzyme involved in melanization, activated through the cleavage of its precursor form prophenoloxidase (PPO) [28]. Thioester-containing proteins (TEPs) play a pivotal role in the regulation of melanization. In *Drosophila*, this process is well characterized. Specifically, the absence of TEP4 leads to increased melanization and PO activity in pathogen-infected mutant *Drosophila* [29]. Similarly, TEP2 mutants exhibit elevated melanization and PO activity against *Photorhabdus* bacteria, highlighting that TEP2 and TEP4 play comparable roles in the immune response to *Photorhabdus* infection [30]. In comparison, TEP6 inactivation decreases PO and melanization in flies following infection, but exhibits lesser susceptibility to bacteria[26]. Further mechanistic studies showed that TEP4 inactivation upregulates the Toll and immune deficiency (IMD) signaling pathways while downregulating the Janus kinase(JAK)/signal transducer and activator of transcription (STAT) and c-Jun N terminal kinase (JNK) pathways upon infection [29]. TEP2 mutants, which do not affect Toll pathway activation but reduce IMD pathway activity, suggest that TEP2 and TEP4 operate through different mechanisms [30]. When *Photorhabdus* infects TEP6 mutants, Toll signaling activity is increased [26].

Currently, research on TEPs’ role in mosquito melanization primarily targets positive regulation of TEP1 in *A. gambiae*. The TEP1 resistance allele confers significant resistance to *Plasmodium berghei*, while also inducing parasite melanization [31]. Mosquitoes from the laboratory L3–5 line, which are homozygous for the TEP1 resistance allele, demonstrate the ability to eliminate *Plasmodium cynomolgi* and *P. berghei* parasites, accompanied by pronounced melanization of the parasites [31]. Moreover, blocking TEP1 in mosquitoes prior to infection raises parasite levels in vulnerable mosquitoes and eliminates resistance and parasite melanization in resistant mosquitoes [17]. However, despite artificially increasing TEP1 expression in transgenic *A. gambiae*, there was no boost in resistance melanization against *Plasmodium* [32]. These findings highlight the challenges of manipulating TEP1-mediated resistance and enhancing our understanding of the molecular mechanisms underlying the regulation of mosquito TEPs on melanization.

This study aimed to elucidate the role of mosquito TEPs in negatively regulating melanization and their involvement in facilitating *Plasmodium* development, which may suggest potential targets for blocking malaria transmission.

## Methods

### Malaria parasites, mosquitoes, and mice

Using cross-mediated insertion, RFP was integrated into the SSU rRNA gene of the *P. yoelii* 265BY parasite to create *P. yoelii* 265BY-RFP [33]. *A. stephensi* was maintained at 27 °C and 70–80% relative humidity, with a 10% sugar solution that included 0.5% para-aminobenzoic acid in a 12-h light/dark cycle room (lights off at 18:00). Female Kunming mice (6–8 weeks old) were provided by the Laboratory Animal Center of the Army Medical University (Chongqing, China), and maintained in a controlled room with a 12-h light/dark cycle (lights off at 19:00).

### Mice infection with malaria parasites

Female Kunming mice (6‒8 weeks; four mice per group) were intravenously infected with 1 × 10^6^ *P. yoelii* 265BY-RFP parasitized erythrocytes.

### Mosquito infection

Female *A. stephensi* mosquitoes, 3–5 days old, were allowed to feed on Kunming mice with gametocytemia exceeding 0.1%, and then received a 10% sugar solution at 23–24 °C. To determine the oocyst burden, midguts from mosquitoes were dissected on day 7 after they had a blood meal. Hemolymph was collected on days 11‒13 to count sporozoites, and salivary glands were dissected on day 17 post-blood-feeding.

### Polyclonal antibody preparation

AsTEP15 (VectorBase Gene ID: ASTE008639) and *A. stephensi* S7 (VectorBase Gene ID: ASTE004816) coding sequences were separately cloned into the pETB2M (GeneCreate Biotech, Wuhan, China) expression vector. Both AsTEP15 and S7 expressions in *E. coli* Arctic-ExpressTM were induced by isopropyl β-d-1-thiogalactopyranoside (IPTG), and the proteins were purified using a nickel column. Rats and New Zealand white rabbits were immunized with AsTEP15 or S7 proteins as antigens. After 3–4 immunization rounds, specific polyclonal antibodies against TEP15 or S7 were purified using an antigen affinity purification column. Experiments were conducted by Zoonbio and GeneCreate Biotech.

### Western blot

Hemolymph and carcasses were collected from uninfected female mosquitoes (*n* = 40). T-PER Tissue Protein Extraction Reagent (Thermo Fisher Scientific, MA, USA) was used to dissolve the samples, which included a Halt Protease and Phosphatase Inhibitor Cocktail from the same company. Approximately 20 µg of the protein samples was blended with Laemmli Sample Buffer (Bio-Rad, CA, USA), heated at 100 °C for 10 min, and subsequently separated on a 10% TGX Stain-Free FastCastTM Acrylamide Kit (Bio-Rad). The isolated proteins were transferred onto a polyvinylidene fluoride membrane and blocked with bovine serum albumin for 1 h at room temperature. To detect TEP15-derived tissue, the membrane was incubated overnight at 4 °C with rat anti-TEP15 diluted to 1:2,000 and rabbit anti-S7 as internal controls in tris-buffered saline with tween 20 (TBST) blocking buffer. After washing with TBST (three times for 5 min), the membranes were incubated with secondary horseradish peroxidase (HRP; 1:2,000; Beyotime Biotechnology, Shanghai, China) for 1 h at room temperature. After the membrane was washed, it was visualized using Takara Biotech Inc.’s Western BLoT HRP chemiluminescence substrate, and ImageJ was used for densitometric analysis for comparison.

### Quantitative real-time polymerase chain reaction analysis

Per manufacturer instructions, TRIzol^™^ reagent (Thermo Fisher Scientific) was employed to isolate total RNA from *P. yoelii 265BY-RFP*-infected mosquitoes. The quality and concentration of each RNA sample was evaluated using a NanoDrop One Spectrophotometer (Thermo Fisher Scientific). RNA (2 µg) was reverse transcribed into first strand cDNA using the PrimeScript™RT reagent Kit with gDNA Eraser (Perfect Real Time) (Takara Bio Inc., Japan). The cDNA produced was subsequently utilized as a template for quantitative real-time polymerase chain reaction (qPCR) with TB Green^®^ Premix Ex Taq^™^II (Tli RNaseH Plus) (Takara Bio Inc.) and primers specific to the target. Using a CFX96 Real-Time PCR Detection System from Bio-Rad, the relative quantitative data were adjusted using the ribosomal protein S7 gene as an internal control.

### RNA-Seq and differential gene expression analysis

After blood-feeding infection with parasites on day 7, total RNA was extracted from a pool of 30 whole mosquitoes using TRIzol^™^ Reagent after injection with AsTEP15 and enhanced green fluorescent protein (EGFP) double-stranded RNA (dsRNA). RNA purification, reverse transcription, library construction, and sequencing were conducted at Shanghai Majorbio Bio-pharm Biotechnology Co., Ltd. (Shanghai, China) per manufacturer instructions. Clean reads were mapped to the reference genome downloaded from VectorBase (*Anopheles_stephensi*_AsteI2 genome) using HISAT2 v2.0 (http://daehwankimlab. github.io/hisat2/) with default parameters. Using the transcripts per million reads method, the expression level of each transcript was calculated to identify differential expression genes (DEGs) between two different samples. Differential expression analysis was performed using DEGseq. Genes were considered significantly differentially expressed if the *q*-value was < 0.001 and |fold change| > 1.2. Gene expression data were deposited in the NCBI BioSample database and are accessible through accession number PRJNA1188637. DEGs were validated using qPCR as described above (Additional file 1).

### dsRNA synthesis and gene silencing

The dsRNA products of all silencing genes, including TEP15, TEP1, and Rel2, were generated using a cDNA template from *A. stephensi* and the MEGAscript^™^ RNAi Kit (Thermo Fisher Scientific), as previously described [34]. Female cold-anesthetized *A. stephensi* (3 days old) were administered 3 mg/mL dsRNA using a Nanoject II injector (Drummond Scientific Co., Broomall, PA, USA) 2 days before infection. dsRNA silencing efficiency was detected using qPCR on day 2 post-knockdown, as described above.

### Statistical analysis

Statistical analyses were conducted using SPSS (IBM, NY, USA). The Student’s *t*-test was used to compare continuous variables between two groups for normally distributed data. Significance was set at *p* < 0.05. Error bars represent the standard deviation.

## Results

### Characterization of AsTEP15 and its upregulation due to *Plasmodium* infection

**Fig. 1:**
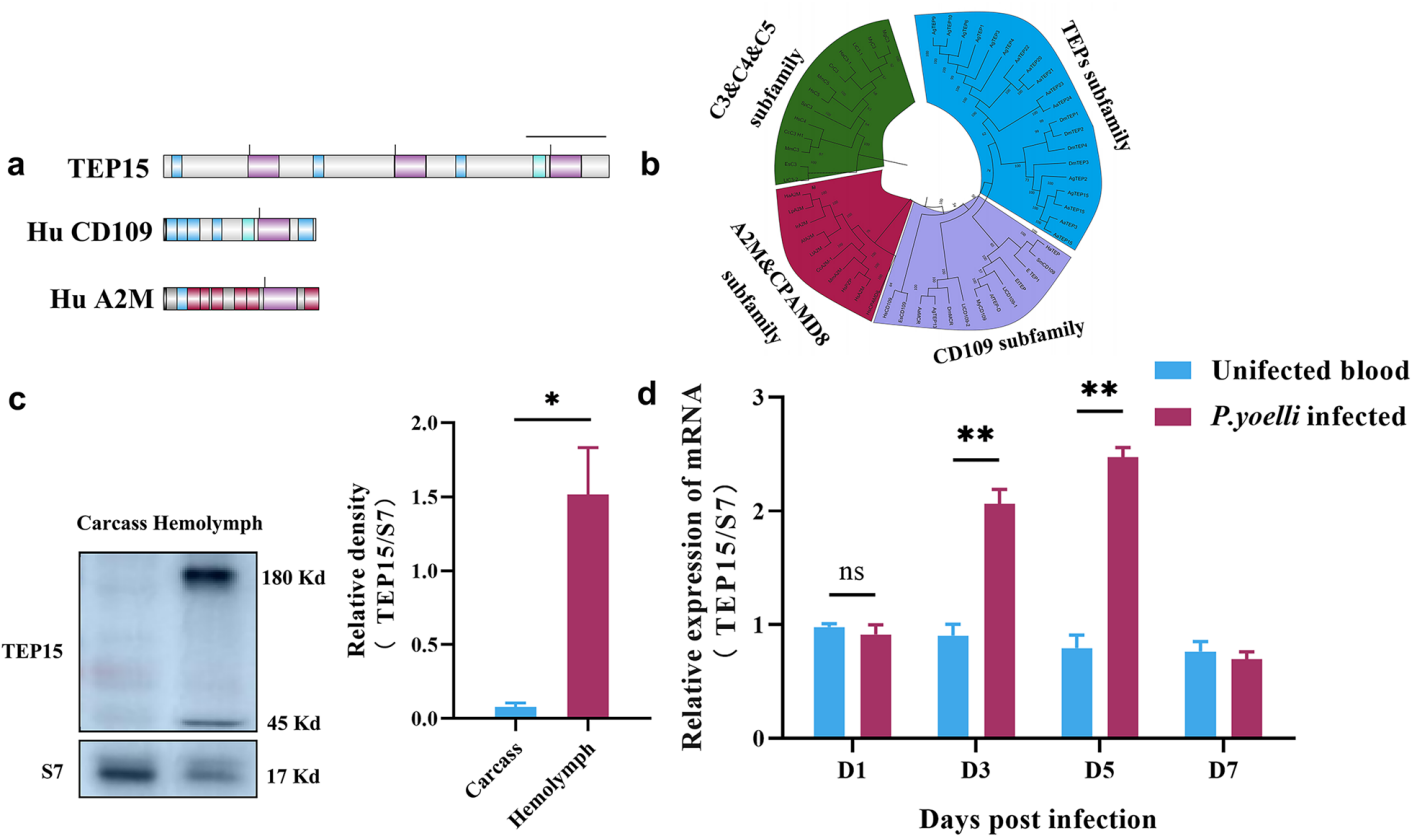
AsTEP15 sequence analysis and its transcriptional changes following *Plasmodium* infection (**a**) sequence comparison. Schematic illustration of proteins including thioester structures: AsTEP15 (VectorBase accession number ASTE008693), human (Hu) CD109, and A2M. Green, complement (C) 3 isoform X1; blue, CD109 isoform; purple, α_2_-macroglobulin-like TED domain; and red-brown, α_2_-macroglobulin. The black vertical line shows the α_2_-macroglobulin-like thioester bond-forming region. The black horizontal line shows the α_2_-macroglobulin/complement system. **b** Unrooted phylogenetic tree of thioester-containing proteins. The evolutionary history was inferred using the neighbor-joining method. The optimal tree is shown. Evolutionary distances were computed using the Poisson correction method and are in the units of the number of amino acid substitutions per site. This analysis involved 56 amino acid sequences, with 5404 positions in the final dataset. Evolutionary analyses were conducted in MEGA11. The invertebrate TEP clades are blue shades, and CD109 clades are purple shades; C3, C4, and C5 clades are green shades; and A2M and CPAMD8 clades are red-brown shades. **c** AsTEP15 immunoblotting analysis. Total protein extracts from hemolymph and carcasses of non-fed female mosquitoes (*n* = 40). The 180 kd band represents putative full-length AsTEP15, and the 45 kd band represents the activation and subsequent hydrolysis of AsTEP15. Hemolymph was collected directly into the loading buffer, immunoblotted with anti-AsTEP15 rat antiserum, and revealed by anti-rat horseradish peroxidase-conjugated antibody. The blot was stripped and reprobed with a polyclonal antibody raised against the S7 protein, serving as a loading control. The molecular weight scale is shown on the right. **d** The mRNA expression level of AsTEP15 in mosquitoes (*n* = 15) fed with *P. yoelii*-infected blood and uninfected blood at the indicated time points post-blood-feeding was determined using real-time PCR. TEP15 transcript levels were normalized to the internal control transcript for ribosomal protein S7. For most of the time points, experiments were performed three times (error bars indicate standard errors)

In an earlier study, we discovered a thioester-containing member with a 12,696 bp transcript and a 4231 amino acid protein. The predicted amino acid sequence featured a canonical thioester motif (GCGEQ), along with CD109 and A2M domains. Subsequently, the newly predicted AsTEP organization was compared with that of human α2-macroglobulin (A2M) and CD109, as detailed in Additional file1: Table S1 and illustrated in Fig. 1a. This AsTEP exhibits the characteristics of known TEP family members, including macroglobulin domains MG2 (pfam PF01835), MG3 (pfam PF17791), and MG4 (pfam PF17789), as well as the complement component domain (pfam PF07678), A2M receptor binding domain (pfam PF07677), A2M receptor (smart SM01361), several CD109 isoform domains, α_-_macroglobulin-like thioester bond-forming region, A_2_M conserved site, and A_2_M family thioester region.

To position this AsTEP within the TEP family, we conducted a phylogenetic analysis using 56 full-length amino acid sequences from the TEP superfamily (Additional file 2: Table S2). In light of the predicted structural characteristics of this TEP, our analysis incorporated complement-like factors (complement components 3, 4, and 5), A2M and pregnancy zone protein-like A_2_M domain-containing 8 (CPAMD8), invertebrate CD109, and TEPs. This analysis was performed using the neighbor-joining method [35] (Fig. 1b). The Poisson correction method was used to calculate evolutionary distances, measured in amino acid substitutions per site, and 5404 positions were identified in the final dataset. The resulting phylogenetic tree displayed the typical two-branch structure of the TEP superfamily, consisting of complement components (Fig. 1b, green shades) and A2Ms. The A2Ms cluster is further subdivided into true A2M (comprising A2M and CPAMD8; Fig.1b, red-brown shades), invertebrate CD109 (Fig. 1b, purple shades), and invertebrate TEP subgroups (Fig. 1b, blue shades). The invertebrate TEP and invertebrate CD109 subgroups formed two closely related clusters. Phylogenetic analysis has confirmed the localization of AsTEP within the invertebrate TEPs subfamily. Due to its close phylogenetic proximity to *Anopheles gambiae* TEP15, this newly identified thioester-containing protein has been designated as *A. stephensi* TEP15 (AsTEP15). The tissue distribution of AsTEP15 was assessed via Western blot analysis. Affinity-purified rat polyclonal antibodies, targeting an *N*-terminal polypeptide of AsTEP15, were generated for this purpose. The findings indicate that AsTEP15 is predominantly present in the hemolymph of adult mosquitoes. Two bands were observed: one approximately 180 kd, likely representing the full-length form of AsTEP15, and another around 45 kd, which may result from the activation and subsequent hydrolysis of AsTEP15 (Fig. 1c, Additional file 3: Fig. S1).

Previous research indicates that invertebrate thioester-containing proteins (TEPs) are involved in immune responses to infections [17]. In this study, we evaluated the differential mRNA expression levels of AsTEP15 in *A. stephensi* mosquitoes under various conditions: blood-fed versus sugar-fed, and *Plasmodium yoelii*-infected versus uninfected. The expression of AsTEP15 was normalized against the ribosomal protein rpS7 gene. Our findings revealed that AsTEP15 expression was significantly elevated, approximately two-fold, in blood-fed female mosquitoes compared with their sugar-fed counterparts (refer to Additional File 4: Fig. S2). In *P. yoelii*-infected mosquitoes, no significant change in AsTEP15 expression was observed on the first day post-infection (PI). However, a significant upregulation was detected on days 3 and 5 PI, with expression levels increasing by 2.0- and 2.5-fold, respectively (Fig. 1d). Transcript levels subsequently declined by day 7 PI. The peak upregulation of TEP15 coincided with the early stages of oocyst development (days 2–5 PI), during which oocysts are enclosed within the basal lamina and interact with hemolymph components. Notably, TEP15 is not upregulated at the time points of ookinete and mature oocyst stages (Fig. 1d; days 1 and 7 PI), and this indicates that TEP15 upregulation may be specific to certain stages of *Plasmodium* in mosquito.

### Silencing of AsTEP15 in mosquitoes inhibits *Plasmodium* development through enhanced oocyst melanization

**Fig. 2:**
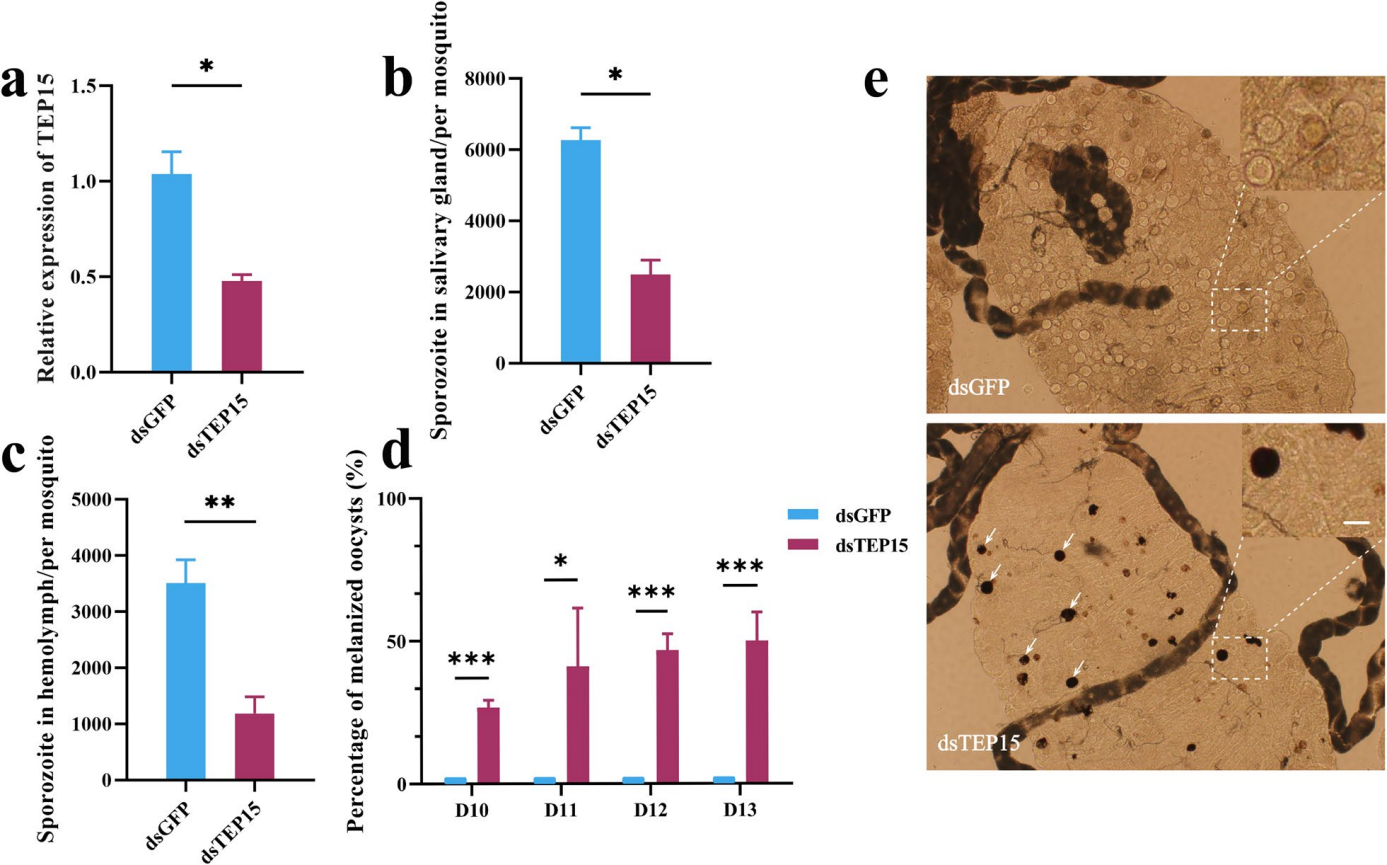
AsTEP15’s role in promoting *Plasmodium* development in mosquitoes. **a** Detection of RNA interference efficiency of AsTEP15. AsTEP15 mRNA levels in *P. yoelii*-parasite-infected mosquitoes (*n* = 15) determined using real-time PCR after AsTEP15 knockdown. **b–c** Number of salivary gland and hemolymph sporozoites in mosquitoes after AsTEP15 knockdown. The average number of salivary gland sporozoites (*n* = 15) **b** and hemolymph sporozoites (*n* = 20) (**c**) in mosquitoes infected with *P. yoelii* were compared after AsTEP15 knockdown. **d** Percentage of melanized oocysts in *P. yoelii*-parasite-infected mosquitoes (*n* = 22) at the indicated time points were compared after AsTEP15 knockdown. **e** Representative image of melanized oocysts in mosquitoes under a light microscope on day 12 post-blood feeding after AsTEP15 knockdown; scale bar 50 μm. Three individual experiments were performed

Due to the upregulation of AsTEP15 caused by *Plasmodium* infection, we aimed to determine whether AsTEP15 promotes or inhibits *Plasmodium* development. To achieve this, we further silenced the in vivo expression of AsTEP15 by injecting dsRNA corresponding to AsTEP15 into the thorax of female mosquitoes. Meanwhile, the controls received dsRNA of unrelated genes. We used qPCR to determine that the silencing efficiency of TEP15 is around 60% (Fig. 2a). The number of salivary gland sporozoites in the TEP15 knockdown control group was three times that in the knockdown group (Fig. 2b). Further observations on the changes in the number of hemolymph sporozoites also revealed that the number of hemolymph sporozoites in the knockdown control group was significantly higher than that in the knockdown group (Fig. 2c), indicating that interfering with the expression of TEP15 can inhibit the development of *Plasmodium* in mosquitoes. To analyze the reason for the reduced number of sporozoites in mosquitoes with TEP15 knockdown, we observed whether the development of *Plasmodium* oocysts in mosquitoes with TEP15 knockdown changed, compared with non-knockdown mosquitoes. Oocyst melanization was observed in the midgut of AsTEP15 RNA interference (RNAi) mosquitoes. The proportion of melanized oocysts in RNAi-treated mosquitoes averages 25% on the 10th day post-infection and increases to 50% by the 13th day (Fig. 2d). Melanized oocysts were not observed in control mosquitoes. Generally, oocyst melanization refers to the presence of varying amounts of black dots inside the oocyst capsule, as shown in the example images from 12 days post-infection (Fig. 2e). Therefore, the silencing of AsTEP15 in mosquitoes inhibits *Plasmodium* development through enhanced oocyst melanization.

### AsTEP15 regulates *Plasmodium* melanization in mosquitoes by negatively modulating AsTEP1

**Fig. 3:**
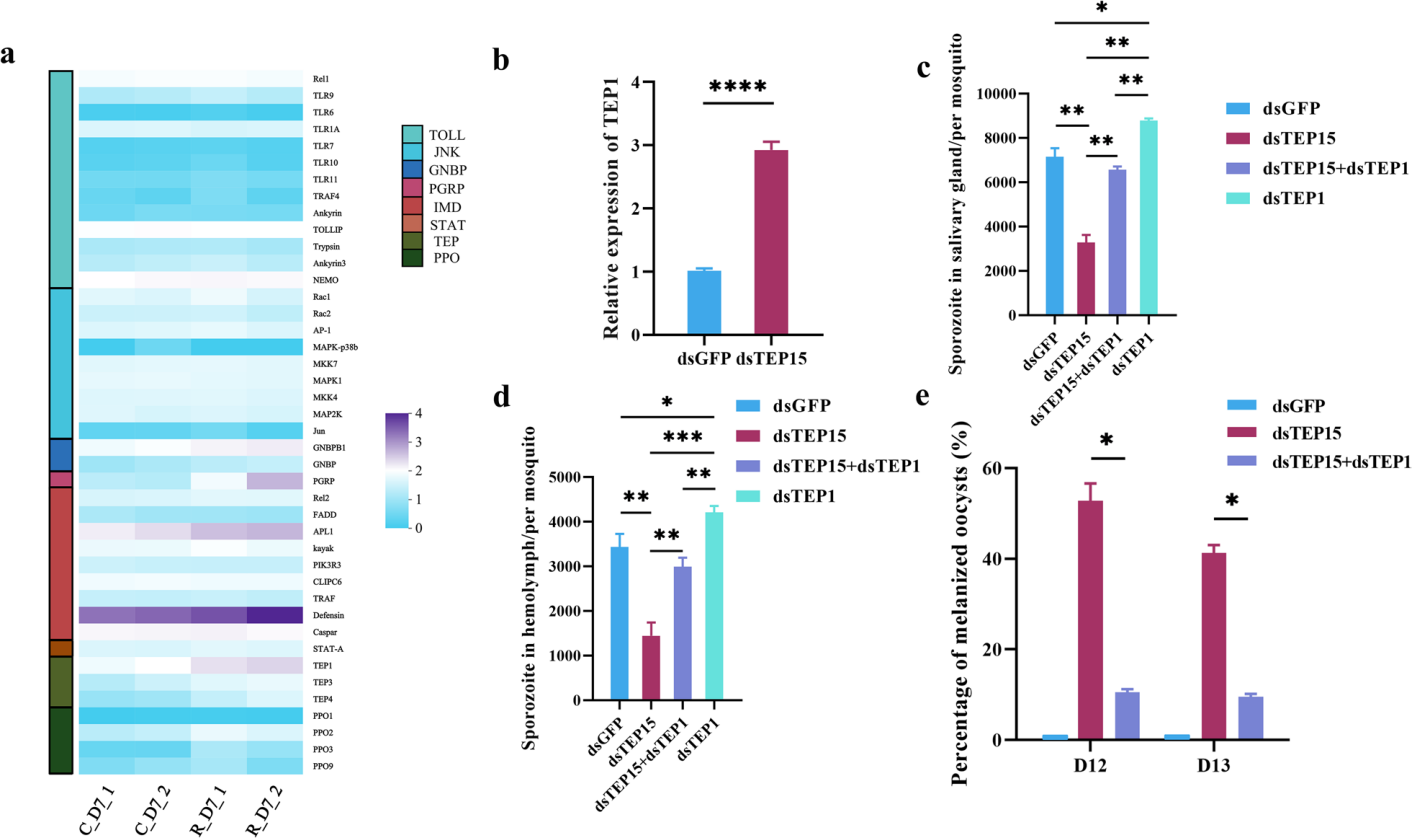
AsTEP15 in regulating TEP1-mediated oocyst melanization. **a** Differential gene expression of immune-related genes in *P. yoelii*-infected mosquitoes after AsTEP15 knockdown. Heatmap comparing the differential expression of immune-related genes between double-stranded green fluorescent protein (dsGFP) and double-stranded TEP15 (dsTEP15) *P. yoelii*-infected mosquitoes (*n* = 30) on day 7 PI. **b** Detection of AsTEP1 expression change after AsTEP15 knockdown. TEP1 mRNA levels in *P. yoelii*-parasite-infected mosquitoes (*n* = 15) determined using real-time PCR following AsTEP15 knockdown at day 7 PI. **c–d** Number of salivary gland and hemolymph sporozoites in mosquitoes after AsTEP1 and AsTEP15 double knockdown. Average number of salivary glands (*n* = 20) (**c**) and hemolymph (*n* = 22) sporozoites (**d**) in *P. yoelii*-parasite-infected mosquitoes were compared with AsTEP1 and AsTEP15 knockdown. **e** Percentage of melanized oocysts after AsTEP1 and AsTEP15 double knockdown. Melanized oocyst percentage in *P. yoelii*-parasite-infected mosquitoes (*n* = 22) at the indicated time points were compared after AsTEP1 and AsTEP15 double knockdown

To elucidate the mechanisms by which AsTEP15 regulates melanization, mRNA was extracted from mosquitoes infected with parasites and injected with AsTEP15 and EGFP dsRNA at 7 days post-infection (PI) for sequencing analysis. The findings revealed that the knockdown of AsTEP15 induced significant transcriptional alterations. Gene ontology (GO) analysis of differentially expressed genes highlighted the innate immune response process and defense response as prominently regulated biological processes (refer to Additional File 5: Fig. S3). Among the predicted immune and defense response genes were those implicated in thioester-containing proteins, the phenoloxidase (PPO) cascade, and immune signaling pathways (Fig. 3a). Given the pivotal role of TEP1 in oocyst melanization [20, 36, 37], our initial focus was on changes in TEP1 transcript levels in the AsTEP15-injected dsRNA group. The results indicated a ~2.5-fold increase in TEP1 expression in the AsTEP15 knockdown group compared with the control group, which was further corroborated by qPCR (Fig. 3a–b; Additional File 6: Table S3).

We further assessed the impact of simultaneous knockdown of AsTEP15 and AsTEP1 on *Plasmodium* development and oocyst melanization in mosquitoes. The results demonstrated that the number of salivary gland sporozoites in the TEP15 and TEP1 co-knockdown group was twice that of the TEP15 knockdown group. Similarly, the number of hemolymph sporozoites in the TEP15 and TEP1 co-knockdown group was also double that of the TEP15 knockdown group. However, the sporozoites number in the TEP15 and TEP1 co-knockdown group remained lower than that in the EGFP control group. Secondly, from the perspective of oocyst development, the rate of melanization in the group with co-knockdown of TEP15 and TEP1 was significantly reduced, with the rate of melanization in the TEP15 knockdown group being approximately five times higher than that in the co-knockdown group. However, melanization was still observed in mosquitoes with double knockdown of TEP15 and TEP1. These results suggest that knocking down of TEP15 and TEP1 can reverse the melanization caused by knocking down TEP15. Thus, we have demonstrated that AsTEP15 promotes *Plasmodium* development in mosquitoes by inhibiting AsTEP1-mediated melanization.

### AsTEP15 modulates *Plasmodium* melanization in mosquitoes by inhibiting the IMD pathway

We investigated the signal pathway by which AsTEP15 modulates oocyst melanization. Mosquitoes are capable of eliciting robust antiplasmodial responses through the activation of various signaling cascades associated with immune regulation, including the IMD, Toll, JNK, and Janus kinase–STAT pathways [38–41]. We analyzed the transcriptome sequencing data and noted gene expression changes in signaling pathways due to AsTEP15 knockdown in mosquitoes. The results showed that AsTEP15 knockdown significantly upregulated the IMD pathway genes, such as PGRP (ASTE000822), APL1 (ASTE016290), Defensin (ASTE011281), and Rel2 (ASTE010 360) (Fig. 3a; refer to Additional File6: Table S3). However, the expression levels of Rel1 (ASTE011378), JNK (ASTE007 551), and STAT (ASTE011642) in the Toll, JNK, and STAT signaling pathways showed no significant variation between the control and RNAi groups (Fig. 3a; refer to Additional File6: Table S3), which was further confirmed by qPCR (Additional file 7: Fig. S4).

**Fig. 4:**
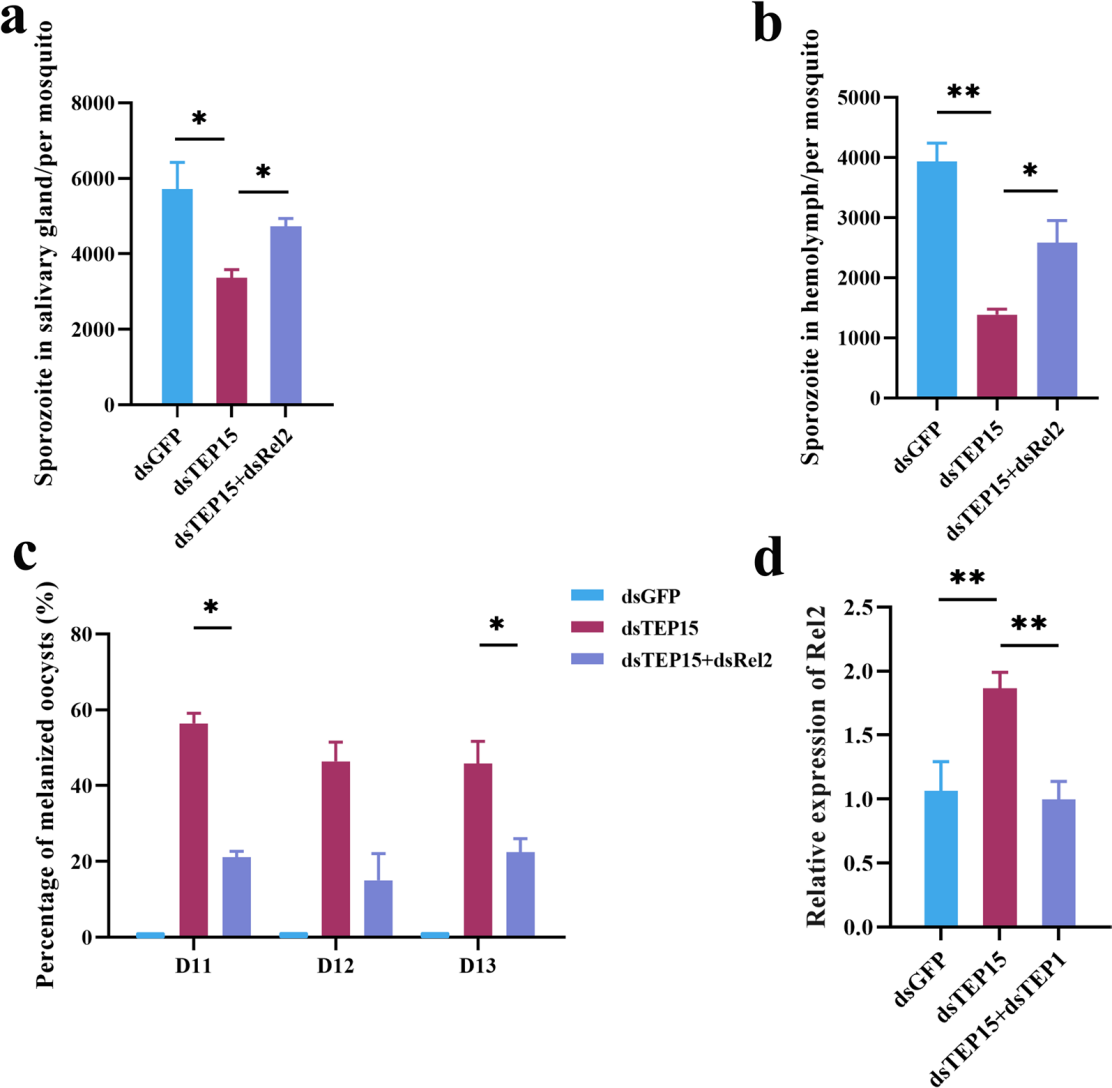
AsTEP15 promotes malaria parasite development by negatively regulating the IMD pathway. **a–b** Number of salivary gland and hemolymph sporozoites in mosquitoes after AsTEP15 and AsRel2 double knockdown. The average number of salivary gland (*n* = 15) (**a**) and hemolymph (*n* = 20) sporozoites (**b**) in *P. yoelii*-infected mosquitoes were compared with AsTEP15 and AsRel2 double knockdown. **c** Melanized oocyst percentage after AsTEP15 and AsRel2 double knockdown. Melanized oocyst percentage in *P. yoelii*-infected mosquitoes (*n* = 22) at the indicated time points were compared after AsTEP15 and AsRel2 double knockdown. **d** Detection of Rel2 expression change after AsTEP15 and TEP1 double knockdown

The role of IMD signaling pathway in the negative regulation of oocyst melanization by AsTEP15 was evaluated through genetic epistasis analysis. The simultaneous knockdown of AsTEP15 and IMD pathway-related gene Rel2 (ASTE010360) led to a significant increase in the number of sporozoites in the salivary glands and hemolymph, by approximately 1.5- and 2-fold, respectively, compared with the AsTEP15 knockdown group. However, the number of sporozoites in the AsTEP15 and Rel2 co-knockdown group remained lower than that in the EGFP control group (Fig. 4a–b). The melanization rate in the co-knockdown group of TEP15 and Rel2 was significantly reduced, with the melanization rate in the TEP15 knockdown group being approximately 2.9 times higher than in the co-knockdown group. Despite the double knockdown of AsTEP15 and Rel2, melanization was still observed in mosquitoes (Fig. 4c). These results suggest that the IMD pathway is implicated in the regulation of melanization by TEP15.

In addition, the activation of these pathways leads to the transcription of effector genes that facilitate the antiplasmodial response. TEP1 plays a critical role in the mosquito’s complement-like system and can be modulated by the Toll, JNK, and IMD pathways [20, 38, 41, 42]. However, the question remains whether TEP15 regulates TEP1-mediated melanization through the IMD signaling pathway. Our initial findings revealed no change in TEP1 expression when comparing the knockdown of AsTEP15 alone to the concurrent knockdown of AsTEP15 and Rel2 (Additional file 8: Fig. S5). Interestingly, when both AsTEP15 and TEP1 are knocked down, Rel2 expression decreases to a level twice that observed with only AsTEP15 knockdown, showing no significant difference from the dsGFP control group (Fig. 4d). These findings suggest that AsTEP15 modulates *Plasmodium* melanization in mosquitoes by inhibiting the IMD pathway.

## Discussion

We identified a TEP gene, namely, AsTEP15. Most TEPs share a distinctive thioester domain that enables them to create stable covalent bonds with target molecules containing hydroxyl or amine groups [43, 44]. TEPs contribute to the ability of the immune system to neutralize or eliminate targets by binding to or tagging molecules, an outcome fundamental to the monitoring and response of the innate immune system. Recent research on mosquitoes has demonstrated that TEPs attach to pathogens such as the malaria parasite *Plasmodium* and Dengue virus, triggering phagocytosis, lysis, and melanization to reduce infection [5, 45]. However, TEPs exhibit diversity through their numerous functions. In addition to positive immunity modulation, can they negatively regulate immunity? The specific negative regulatory molecular mechanism is also unclear. In this study, we demonstrated that AsTEP15 from *A. stephensi* hemolymph can be upregulated with *Plasmodium* infection. Moreover, AsTEP15 promotes malaria parasite development in mosquitoes by inhibiting melanization.

Putative polypeptide sequence analyses revealed that AsTEP15 includes domains conserved across other TEP superfamily members, including the macroglobulin, thioester, CD109 isoform domains, and A2M-receptor domains (Fig. 1a). Moreover, the amino acid sequence of AsTEP15 was further compared with human CD109, and A2M sequences showed that AsTEP15 differs from the above two molecules, highlighting that AsTEP15 might have unique physiological functions compared with vertebrate CD109 and A2M. The multifunctional GPI-anchored protein CD109 is important in the progression of tumors and the maintenance of physiological homeostasis, emphasizing its potential as a biomarker for diagnostic significance and the deregulation of immune checkpoint molecules [46]. In insects, a CD109 was found in *Helicoverpa armigera* larvae; the *H. armigera* CD109 (HaCD109) gene shows high expression levels in hemocytes, with notable upregulation upon stimulation by *Escherichia coli* and chromatography beads [47]. Silencing HaCD109 in vivo significantly increased bacterial load within larval hemolymphs and decreased hemocyte dispersion [47]. However, in our study, AsTEP15 silencing notably reduced the number of sporozoites in the mosquito salivary gland and hemolymph. Therefore, molecules with the CD109 domain may be involved in immune regulation for different infections.

To analyze AsTEP15’s involvement in the immune regulation of malaria parasite infection in mosquitoes, its expression changes were first observed. AsTEP15 was upregulated at days 3 and 5 PI (Fig. 1d), coinciding with mosquito early oocyst development. Previous findings have suggested that the mosquito immune response constrains oocyst growth [48–50]; moreover, in comparison to ookinetes, oocysts are less affected by the mosquito’s immune defenses [51]. This implies that oocysts have devised ways to avoid mosquito immune defenses. The preliminary research showed that malaria oocysts need circumsporozoite protein (CSP) to evade mosquito immune responses [20]. This study showed that malaria parasites may also induce the expression of immune negative regulatory molecules in mosquitoes at the oocyst stage, which is beneficial for *Plasmodium* development.

Further, RNA interference experiments revealed that AsTEP15 knockdown reduces the number of sporozoites in salivary glands and hemolymph (Fig. 2b–c) and results in oocyst melanization in mosquitoes (Fig. 2d–e). Melanization is an important mosquito immune response against malaria parasites. Studies have also found that mosquito melanization reactions occur during the ookinete [45] or oocyst stage [52]. Further research was conducted on the molecular mechanism of the melanization caused by AsTEP15 knockdown. Transcriptome sequencing revealed that knocking down AsTEP15 leads to transcriptional changes. The transcription change genes related to immunity include thioester containing proteins, PPO cascades, and immune signal pathways (Fig. 3a). Subsequently, we employed a genetic epistasis analysis experiment to examine the involvement of these critical immune genes in the inhibition of mosquito melanization by TEP15. The double knockdown of AsTEP15 and AsTEP1 can reverse the decrease in the salivary gland and hemolymph sporozoites and oocysts melanization caused by AsTEP15 single knockdown (Fig. 3c–e). However, melanization still occurred in the double knockdown group, which did not return to the rate observed in the EGFP knockdown control group. We further analyzed that the reason for the occurrence of melanization after knocking down the expression of TEP1 may be due to incomplete knockout.

In addition, transcriptome sequencing data revealed that, relative to the control group, the knockdown of AsTEP15 led to an approximate twofold increase in the expression of PPO1 and PPO2, a sixfold increase in PPO3, and a 1.3-fold increase in PPO9 (Additional file 6: Table S3). Previous studies have also found that important molecules related to PPO cascade reactions include PPO1, PPO2, PPO3, and PPO9, which play a role in melanization [53–55]. Moreover, a network of undirected gene co-expression was built to arrange all *A. gambiae* genes from families experimentally proven to aid melanization [22]. This network includes genes from the serpin (SRPN), PPO, and TEP families [22]. Subsequent co-immunoprecipitation (CoIP) experiments revealed 21 proteins that bind directly or indirectly to SRPN2 in the hemolymph of adult female mosquitoes 24 h after exposure to lyophilized *Micrococcus luteus* bacteria, including TEP15, TEP1, and PPO1 [22]. Thus, this study further indicates that PPO, in conjunction with TEP15 and TEP1, plays a role in the regulation of melanization in mosquitoes.

Moreover, transcriptome analysis revealed significant changes in the IMD immune signaling pathway caused by AsTEP15 knockdown in mosquitoes (Fig. 3a; refer to Additional File6: Table S3). Genetic epistasis analysis revealed that AsTEP15 and AsRel2 double knockdown significantly increases the number of sporozoites in salivary glands and hemolymph, and significantly reduces the melanization rate, reversing sporozoite numbers and melanization rate changes caused by AsTEP15 knockdown (Fig. 4a–c). The results suggest that AsTEP15 negatively influences the IMD pathway, regulating *Plasmodium* melanization in mosquitoes. However, our study failed to demonstrate that TEP15 negatively regulates TEP1-mediated melanization through the IMD pathway (Additional file 8: Fig. S5). Previous research showed the relevance of the IMD pathway components and regulated effector TEP1, in parasite infection intensity-dependent defense [42]. However, in our present research, we only discussed a single malaria parasite infection density. Previous research also determined that the IMD pathway is most potent against the parasite’s ookinete stage, yet also has reasonable activity against early oocysts and lesser activity against late oocysts [42]. We found that AsTEP15 may play a role in early oocysts. Consequently, in subsequent research examining the role of the IMD pathway in TEP1-mediated melanization, which is negatively regulated by TEP15, it is essential to incorporate a range of malaria parasite infection densities and various developmental stages of the malaria parasite within mosquitoes. Interestingly, double silencing of TEP15 and TEP1 inhibited the transcription factor Rel2 in mosquitoes (Fig. 4d). A similar phenomenon was observed in *Aedes Aegypti*, which showed that TEP1 also positively regulates the transcription factor Rel2 of the IMD pathway and consequently controls Dengue virus replication [5]. Overall, the above sequencing and RNAi experimental results suggest that these gene expression changes are consistent with the interpretation that AsTEP15 is counteracting AsTEP1-mediated melanization in *A. stephensi* infected with *P. yoelii*, and it is also possible that TEP15 inhibits the melanization by negatively affecting the IMD pathway and TEP1 in parallel.

## Conclusions

We found a thioester-containing member in *A. stephensi* induced by *Plasmodium* infection and designated this thioester-containing protein as AsTEP15. Further research showed that AsTEP15 knockdown induced mosquito resistance to malaria parasite melanization during the oocyst stage and significantly reduced sporozoite numbers. Furthermore, TEP15 inhibits the melanization by negatively affecting the IMD pathway and TEP1 in parallel. Thus, we described a TEP that can negatively regulate TEP1 and affect malaria parasite growth within mosquitoes. However, the observed variations in the impact of AsTEP15 on malaria parasites may be attributed to discrepancies between our study and previous research regarding the infection of *P. yoelii* and *P. falciparum*. Additionally, differences in our predicted protein sequence alignment with AsTEP15 from prior studies may arise from referencing different genomes of *A. stephensi*, or from variations in transcripts or subtypes. Further investigation is required to substantiate these findings. Our study clarifies the diverse functions of TEPs and could uncover an additional strategy for oocysts to utilize mosquito TEPs to escape and impair mosquito immune defenses.

## Supplementary Information

Additional file 1: Table S1. List of thioester-containing proteins, amino acid sequences used for sequence comparison.

Additional file 2: Table S2. List of 56 amino acid sequences used for an unrooted phylogenetic tree.

Additional file 3: Fig. S1. Full Western blot results.

Additional file 4: Fig. S2. The differential mRNA abundance of AsTEP15 in uninfected blood-fed versus sugar-fed mosquitoes.

Additional file 5: Fig. S3. Gene ontology (GO) analysis of differential gene expression in *P. yoelii*-infected mosquitoes after AsTEP15 knockdown on day 7 post-infection (PI).

Additional file 6: Table S3. List of abbreviations and gene names in the heatmap comparing the differential expression of immune-related pathways genes.

Additional file 7: Fig. S4. The expression levels of genes discovered in transcriptome sequencing were further confirmed by quantitative PCR experiments. (**a**) Detection of changes in the expression of AsAPL1 after AsTEP15 knockdown. The mRNA levels of APL1 in *P. yoelii*-parasite-infected mosquitoes (n = 15) were determined by using real-time PCR following AsTEP15 knockdown at day 7 PI. (**b**–**d**) Detection of immune-related signal pathway expression changes after AsTEP15 knockdown. The mRNA levels of JNK, Rel1, and STAT-A in *P. yoelii*-parasite-infected mosquitoes (*n* = 15) determined using real-time PCR following AsTEP15 knockdown on day 7 PI.

Additional file 8: Fig. S5. Detection of AsTEP1 expression change after AsTEP15 and AsRel2 double knockdown.

## Data Availability

Data are provided within the manuscript or supplementary information files.

## Abbreviations

α2Ms: α2-Macroglobulins
AgTEP1: *Anopheles gambiae* TEP1
AsTEP1: *Anopheles stephensi* TEP1
DEGs: Differential expression genes
GPI: Glycosylphos-phatidylinositol
GO: Gene ontology
IMD: Immune deficiency
iTEPs: Invertebrate TEPs
PO: Phenoloxidase
PPO: Prophenoloxidase
qPCR: Quantitative real-time polymerase chain reaction
Rel2: Relish-like protein 2
TEPs: Thioester-containing proteins
TED: Thioester domain
TGF-β: Transforming growth factor-beta
