# Supplementary material for: Thioester-containing protein TEP15 promotes malaria parasite development in mosquitoes through negative regulation of melanization
Source: Parasit Vectors. 2025 Apr 1;18:124. doi: 10.1186/s13071-025-06772-5 (PMC11963550; doi:10.1186/s13071-025-06772-5)
Supplement: Supplementary file 1 — Additional file 1: Table S1. List of thioester-containing proteins, amino acid sequences used for sequence comparison. [file 13071_2025_6772_MOESM1_ESM.pdf]

Additional file 1: Table S1. List of thioester containing proteins, amino acid sequences used for sequence comparison.

Name	Amino Acid Sequences
**AsTEP15**	MIVTNISPTKKEFECEIVDSNDVIVGWYPMAVPPYGLKKALMRVEGLEGKGYKLQVWDKHKRTLLNSTALECIRKSYLVLFQTDKPAYKPGDR<br>VQFRVIILYPNAAPVIRIQPDFFITDPDRLRMKQWLNATLTSGVFEGSFQLAEQTTVGLWTISTSLFDQQYKDSFLVEEYTLPLFKIETQSVPKRLL<br>HCKDPKMSLKLRASYVQGGAVRGNATVIVRTHFNNYPSQTKEVARKNLPINGVALVNFPTDLVAKNCDEERTVWFDVKVSESSTGVSYNATS<br>TMTVHNSEGVTMEVLDGKDAFYPGQPMRVKVKVATILDERPLVRRNVRILYRVVDEDHDERDEAMPNVLVLQTNGNGIVHFTVNTTIKTVEV<br>NVEGMYNNQTIPLVFAYPMYEDKSFDYLEMHSRNAYHTLDRNITIDLYSNVMLQRIYYVGYCMSRIAVHGVHEAKNPVKHHRLEIEPIRLMNP<br>RMKLLAYAMKDDGKILSSAIIIRFKPTSSTALNITATLKEPNSGKYEFNVTTEEEAFVGLLGVDERILQRSTIDNNISQRKLDKEMQDFEDPSGVW<br>SPYDSFGSVGMTILTDGYLPDVGFVPHLSELARAGTAPEQDYSTREDFPESWIWESAQAPNGKALFEKSLPDTITTWIVTGFSVSQRNGLQILKEP<br>LKINSKKWIFAQLHMPPSIKRFEEVTVHCLVHNYGAAANVTVEVRPKLKAVTPLFLAQGATETVRIKLKSASVGELAVEVILRGPTGKVIDALHQ<br>TIPVRPEGLIKTVEDVRVLSFPRHGKQNFSLSLPAIEKDRGTSSGEVTLSVIGTILNLNFFDLEHMVKSSHGNGEENLLFLQTTMAVYDYLEKTNR<br>LPPASKEKLLTYMEVAYQQMLSHRLEDGSYSTFKRIHKCGDVWFTASALVALQKLAKYIPVEDSLLTDSLDWLVLNSAEDGGYNESCTIVHPHI<br>QRTGGRELSLASSVLFAFVGGGSSKQYEQLTKKTVSLLLSSPIEDVYLLAKTTYLLSLMGHPESVGMLVTLNTMAVTDGKYRFWRVARRESTSL<br>VDMRDQEATAYALLANLKQNTIDQVEMVRVAQWLQKHSFAGDRCTPSLERVIALEALATVAHLIPSIVPSMYIAVGNVRFEVNATNKELLQTV<br>TLPKGTRSVSVSVRGSGLVLIKLSYRYSLANATTTGKTASNRVPNKGPIGVNIKKKAVKNSMLALHMCFSLIQPLYVYESDLWKAAIDLPTGYEI<br>DEQQQRYDNTTKSAILLENNSHLELVWDVRHVTEVCYAVTAISRLLPVDLMPGLIYLSSFDTKDIVAKFKQCFLRDVEYHLVVKCSELLPIRKTI<br>KMGKPSHYSIIGSRILRPNSVYCVQVSTFDTKSPVAFRISIVAKEKSIKSEEITLTGNESRLLRFTIDSIPEDEYELMAEGLSGLVFKTKAHIDFDNKF<br>CSVLIQTDKSVYKPGDTVRYRVLVLDRYMKPLPVDDGGMMVYIRDGKGNRVKQWNDASLGECGVFQSELTLSTEPVLGAWDINVDVLGLKH<br>TKSFDVDEYVLPTYEVTLESPGHTFLDDELLKLVINSKYTYGKPVVGELTVSIKVGQSMCFGRGPNTSSICQKVVPTEGRTVVEFNLKEILLNKM<br>YIRELQIEAEVCEALTGRTQQGSTTVTLHENRFEVRFIEESSYFPGLPYNAWIQVTNLDGSPVREGTREVEIALRNYNTDLFKQTGTLDDAGKVK<br>LNVQLDELTFDYVSVEVKFRGKDYYVQGITKPRADQEAFMKAMPLEKLTIPGTELKFDVACTKPLQLVAYSLLARGELLAGGAVQGADNKQT<br>SITIPSSYCMVPRAKLLVHYISPTGYIVSTYTEVKFGRLFENQIQLTLSKDEVKPGESLDIDVRTEEGSFVGLLAVDQSVLLLKSGNDIGREQIEQEL<br>EMYESSQSHHRYWNNNSTSDCQSVGAVLLSNRFIPKDIFPRGVMFACRAAPMGGVFGAAPQMMLKRANMPMMEADMMMESAAEPTIRANFP<br>ETWIWESIINSKETESIRKIVPDTITSWIITGFSLSKSQGFGLMDSPSKVNVFMPFFVTIDLPYSVKLGETVRIPVIVFNYMDEDQTADVVFFNDNDE<br>FEFFSDDAADQQDKHRQEKLPVSRGGGKTLTFMIKPTKVGHITLKLTAKCALAGDGIERQLLVEPEGLPQYINKALLIDLRSVKEVSQTFEVQVP<br>ADAVPDSTKVEVSVIGDVLGSSIENLDSLIRMPFGCGEQNMLNFVPCIVVLDYLKACRRLTVEIESKAKKCMEVGYQRELTYKHQDGSFSAFGES<br>DKSGSTWLTAFVAKSFQQAAAHITIEEDVIDKALQWLSKIQSADGAFPEVGSICHKDMQGGAGSGIALTAYTVVAFLENAKLGEKFKSTVDKAL<br>AYIQQHIAELDDVYAHALAAYALQIAKHSLKDEVLAGLQSKATKEGDMQWWTKSLPEKTETDCWWHRPCSVNVEMSAYGLLATLETCTGLE<br>GLPIMKWLVSQRNDKGGFESTQDTVVGLQALSKMAAQLSSSEADVSIKVTAPNGQERNISVNKHNTLVLQKHELAVDTKKVDMAATGTGCAL<br>FQLSYKYNIKDVDKAPRFMLKSEAKKGSIKSCIDLTVTTSFIPQEDQAVSNMAVMEVDMPSGFIVEADVLKQLKELELVKKVETKRGNTTVVLY<br>FDNVSEDVIKLRMSAFQKHEVENAKPANVIIYDYYDNSNHYSIVGAKLLRPNSEYHVAVTNQDVSEPIRFSLAITDASNVIEKQEITLNTGETRLV<br>PFVIGDIPESSYKLVAEGLSGLTFKNETDLEYQQKSFSVFVQTDKSIYKPGDTVRFRVLVLDPNTKPLPKADSINVHINDAKANRIKQWKEGKLV<br>KGVFESELTLSTAPVLGAWTINVEVLGTKHNKVFEVDEYVLPKFEVTVESPGITTFKDGKVKAIIRSKYTYGKPVKGEATVSASPEFQFHYVQPF<br>AKDVITRKVVPIDGKGSVEFDLREELRLEGDYTRNIVIEAVVEEELTGRKQNASAKVMIYDRRYKMELIKSDDNFKPGLPYTAWLKVTYQDGAP<br>VQDQTNPVEVKQSTYESTQFLRNYTLDQNGMAKLEINTDVNSTYINVVGVYLGQEFYLSGISKADSDVDSYIRARVLTEMPLVGSDVVVEVSA<br>TAPMKFFTYQLLGRGDVLLSNTVAVPESKTHKFKFPATFAMVPRAKLVVFYIAPNGDMVSDSKVITFDSELQNFMKVSLSKDQTKPGQDVEISIS<br>TNPDSYVGLLGVDQSVLLLKSGNDITKEQVFSELEKYEERSYGYYRRKKRFAWNPHIEHQDFNTVGAFVLSNANDPPHDVIDIRVAEEDRIGGPL<br>LQYAPSTSSLGPAGDASSSPTPAVRKSFPESWIWHTIARFEQLQYSHAHVEMSAIKFSASQSQTGFRLCMRPGERANNTLPEVPIYKFHRAPNGTV<br>LYTTIEKPRSMQNQRQVLKTNTRPPLAGPFAFSRIPRPHRDIPRIFLSQEIQNTWLFDNTYSGFSGEKTLQKKVPDTITSWIITGFSVNPVYGLGLTQ<br>QPRKLKVFLPFFVSTNLPYSVKRGEVVAIPIVVFNYMEDDQTAEVVLHNDEQEFEFADVENEVVESSKVELFRQKRLDIASNTGKSVSFMVKPK<br>KLGHITIKVTAKTKIAGDAVERQLLVEPEGLPQFINKAAFIDLRAVPEATKTFEVEIPKNAVPDSTRIEVAVIGDVMGSTIQNLDSLIRMPYGCGEQ<br>NMLNFVPNIVVLDYLKATNKLTANIEAKAKKFMEAGYQRELSYKHQDGSFSAFGESDKSGSTWLTAFVARSFKQAANHITIDEKVIDKSLEWLS<br>DHQAPNGSFPEVGVVSHKDMQGGSGSGVALTAYTLIAFLENINLVDKYKNTINKAIDYVYRNTESLDDTYALALAAYALQLADHSSKQLILSKL<br>DAKATTDSDSKWWHKPIPEVEQKNPWYSRPNSVNVEMSAYGLLAFLEAGLDTDALPIMKWLIGQRNDKGGFQSTQDTVVGLQALAKLAAKIT<br>SPNNDVAIVAKINENQEKRMSVNAENGMILQKFELPSAARNIEIKATGSGFAVVQLSYKYNMNVTGEWPRFVLDPQVNANTNPDHLHLSVCAS<br>FVPSAGQNVSNMAVMEVGFPSGFTADSDTLPSLENMPFIKKVETKDGDTTVVLYFDSLDQRELCPTISAFRTHKVAKQKPAPVVIYDYYDNSRI<br>ARQFYEGPKASLCDICENEDCSEACSIKSQKQRSSDSPSREPTVDNGPTLSSSSPTVRISFFTFLFATVLVTIFH
**Hu CD109**	MQGPPLLTAAHLLCVCTAALAVAPGPRFLVTAPGIIRPGGNVTIGVELLEHCPSQVTVKAELLKTASNLTVSVLEAEGVFEKGSFKTLTLPSLPLN<br>SADEIYELRVTGRTQDEILFSNSTRLSFETKRISVFIQTDKALYKPKQEVKFRIVTLFSDFKPYKTSLNILIKDPKSNLIQQWLSQQSDLGVISKTFQL<br>SSHPILGDWSIQVQVNDQTYYQSFQVSEYVLPKFEVTLQTPLYCSMNSKHLNGTITAKYTYGKPVKGDVTLTFLPLSFWGKKKNITKTFKINGSA<br>NFSFNDEEMKNVMDSSNGLSEYLDLSSPGPVEILTTVTESVTGISRNVSTNVFFKQHDYIIEFFDYTTVLKPSLNFTATVKVTRADGNQLTLEERR<br>NNVVITVTQRNYTEYWSGSNSGNQKMEAVQKINYTVPQSGTFKIEFPILEDSSELQLKAYFLGSKSSMAVHSLFKSPSKTYIQLKTRDENIKVGSP<br>FELVVSGNKRLKELSYMVVSRGQLVAVGKQNSTMFSLTPENSWTPKACVIVYYIEDDGEIISDVLKIPVQLVFKNKIKLYWSKVKAEPSEKVSLR<br>ISVTQPDSIVGIVAVDKSVNLMNASNDITMENVVHELELYNTGYYLGMFMNSFAVFQECGLWVLTDANLTKDYIDGVYDNAEYAERFMEENE<br>GHIVDIHDFSLGSSPHVRKHFPETWIWLDTNMGYRIYQEFEVTVPDSITSWVATGFVISEDLGLGLTTTPVELQAFQPFFIFLNLPYSVIRGEEFALE<br>ITIFNYLKDATEVKVIIEKSDKFDILMTSNEINATGHQQTLLVPSEDGATVLFPIRPTHLGEIPITVTALSPTASDAVTQMILVKAEGIEKSYSQSILL<br>DLTDNRLQSTLKTLSFSFPPNTVTGSERVQITAIGDVLGPSINGLASLIRMPYGCGEQNMINFAPNIYILDYLTKKKQLTDNLKEKALSFMRQGYQ<br>RELLYQREDGSFSAFGNYDPSGSTWLSAFVLRCFLEADPYIDIDQNVLHRTYTWLKGHQKSNGEFWDPGRVIHSELQGGNKSPVTLTAYIVTSLL<br>GYRKYQPNIDVQESIHFLESEFSRGISDNYTLALITYALSSVGSPKAKEALNMLTWRAEQEGGMQFWVSSESKLSDSWQPRSLDIEVAAYALLSH<br>FLQFQTSEGIPIMRWLSRQRNSLGGFASTQDTTVALKALSEFAALMNTERTNIQVTVTGPSSPSPVKFLIDTHNRLLLQTAELAVVQPMAVNISAN<br>GFGFAICQLNVVYNVKASGSSRRRRSIQNQEAFDLDVAVKENKDDLNHVDLNVCTSFSGPGRSGMALMEVNLLSGFMVPSEAISLSETVKKVE<br>YDHGKLNLYLDSVNETQFCVNIPAVRNFKVSNTQDASVSIVDYYEPRRQAVRSYNSEVKLSSCDLCSDVQGCRPCEDGASGSHHHSSVIFIFCFK<br>LLYFMELWL
**Hu A2M**	MGKNKLLHPSLVLLLLVLLPTDASVSGKPQYMVLVPSLLHTETTEKGCVLLSYLNETVTVSASLESVRGNRSLFTDLEAENDVLHCVAFAVPKS<br>SSNEEVMFLTVQVKGPTQEFKKRTTVMVKNEDSLVFVQTDKSIYKPGQTVKFRVVSMDENFHPLNELIPLVYIQDPKGNRIAQWQSFQLEGGLK<br>QFSFPLSSEPFQGSYKVVVQKKSGGRTEHPFTVEEFVLPKFEVQVTVPKIITILEEEMNVSVCGLYTYGKPVPGHVTVSICRKYSDASDCHGEDSQ<br>AFCEKFSGQLNSHGCFYQQVKTKVFQLKRKEYEMKLHTEAQIQEEGTVVELTGRQSSEITRTITKLSFVKVDSHFRQGIPFFGQVRLVDGKGVPI<br>PNKVIFIRGNEANYYSNATTDEHGLVQFSINTTNVMGTSLTVRVNYKDRSPCYGYQWVSEEHEEAHHTAYLVFSPSKSFVHLEPMSHELPCGHT<br>QTVQAHYILNGGTLLGLKKLSFYYLIMAKGGIVRTGTHGLLVKQEDMKGHFSISIPVKSDIAPVARLLIYAVLPTGDVIGDSAKYDVENCLANKV<br>DLSFSPSQSLPASHAHLRVTAAPQSVCALRAVDQSVLLMKPDAELSASSVYNLLPEKDLTGFPGPLNDQDNEDCINRHNVYINGITYTPVSSTNE<br>KDMYSFLEDMGLKAFTNSKIRKPKMCPQLQQYEMHGPEGLRVGFYESDVMGRGHARLVHVEEPHTETVRKYFPETWIWDLVVVNSAGVAEV<br>GVTVPDTITEWKAGAFCLSEDAGLGISSTASLRAFQPFFVELTMPYSVIRGEAFTLKATVLNYLPKCIRVSVQLEASPAFLAVPVEKEQAPHCICA<br>NGRQTVSWAVTPKSLGNVNFTVSAEALESQELCGTEVPSVPEHGRKDTVIKPLLVEPEGLEKETTFNSLLCPSGGEVSEELSLKLPPNVVEESAR<br>ASVSVLGDILGSAMQNTQNLLQMPYGCGEQNMVLFAPNIYVLDYLNETQQLTPEIKSKAIGYLNTGYQRQLNYKHYDGSYSTFGERYGRNQGN<br>TWLTAFVLKTFAQARAYIFIDEAHITQALIWLSQRQKDNGCFRSSGSLLNNAIKGGVEDEVTLSAYITIALLEIPLTVTHPVVRNALFCLESAWKT<br>AQEGDHGSHVYTKALLAYAFALAGNQDKRKEVLKSLNEEAVKKDNSVHWERPQKPKAPVGHFYEPQAPSAEVEMTSYVLLAYLTAQPAPTSE<br>DLTSATNIVKWITKQQNAQGGFSSTQDTVVALHALSKYGAATFTRTGKAAQVTIQSSGTFSSKFQVDNNNRLLLQQVSLPELPGEYSMKVTGEG<br>CVYLQTSLKYNILPEKEEFPFALGVQTLPQTCDEPKAHTSFQISLSVSYTGSRSASNMAIVDVKMVSGFIPLKPTVKMLERSNHVSRTEVSSNHVL<br>IYLDKVSNQTLSLFFTVLQDVPVRDLKPAIVKVYDYYETDEFAIAEYNAPCSKDLGNA
