# Supplementary material for: Thioester-containing protein TEP15 promotes malaria parasite development in mosquitoes through negative regulation of melanization
Source: Parasit Vectors. 2025 Apr 1;18:124. doi: 10.1186/s13071-025-06772-5 (PMC11963550; doi:10.1186/s13071-025-06772-5)
Supplement: Supplementary file 2 — Additional file 2: Table S2. List of 56 amino acid sequences used for an unrooted phylogenetic tree. [file 13071_2025_6772_MOESM2_ESM.pdf]

Additional file 2: Table S2. List of 56 amino acid sequences used for Unrooted phylogenetic tree.

Name	Amino Acid Sequences
**AgTEP2**	MFSKGGGMRFGGEVKRTVPDPKDHKDGHYSIIGARILRPNSVYRCVVSTFDTKSAIVFRISIAAKDKPIATEEITLNSNESRLIS<br>FTIDSIPEEEYELVAEGLSGLEFKTKSRLDFDNKFCSVLIQTDKSVYKPGDTVRYRVLVLDRSMKLLPAGDSGMMVYIRDGKG<br>NRIKQWSNASLGECGVFQAELTLSTEPVLGEWTINVEVVGLKESKTFDVDEYVLPTYEVTVESPGYTFLDDELLKVVVNSKY<br>TYGKPVAGELTVSVKLASSMCFRREPTETSICQKVLPIDGKTDVEFNLKEILSSKTYIRELTIEAEVCETLTGRTQKGSTTVQLH<br>DERYQVRMIEESSYFPGLPYNAWIQVTNLDGSPVQDGAKEVEIVLRNYNIDLHKQSSTLDDKGMAQLNVKLDELDFDYVSV<br>EVKYRGKDYYVQGITKPRDYEEALMRVRLSEKEPTAGKDLTFDVACTKPLQCVSYSLLARGELLAGGAVKGSEASTTISITIP<br>STFAMVPRAKLLVHYISSAGYIVSSYDTVEFKRVFENQIQLTLSKDELKPVETLDIDIRTEKDSFVGLLAVDQSVLLLKSGNDIS<br>RDEVVQQLEMYESAQNYHWDAYSTSDCQSVGAVLLSNRFIPRDIFPQARLFACSTSAGGFGAAPMMAACKMKGVIMESEM<br>ATAPVNEPTVRSKFPETWIWESISKCKEMESIRKIVPDTITSWIITGFSLSKSHGLGLVDNPSKVNVFMPFFLSIDLPYSVKLGET<br>IRIPVVVFNYMDEDQLADVIFYNNDDEFEFVSDTKDQKEKHRQEQITVPRGTGKTLTFVLKPTKVGHVTLKITAKCALAGDGI<br>ERQLLVEPEGLPQYINKALLVDLRLVKEIKQPFEVEIPVDAVPDSTNVEVSVIGDVLGSSIENLDSLIRMPFGCGEQNMLNFVP<br>CIVVLDYLKACKRLTVEIESKAKRCMEIGYQRELTYKHQDGSFSAFGESDKSGSTWLTAFVAKSFQQAAKHMTIEEDVIDSA<br>LGWLSKVQTADGAFPEVGTICHKDMQGGAGSGMALTAYTVIAFLENPKLGEKYKASVDKALTYVKEHISELDDVYAHALA<br>AYALQIADHPLKNEVYASLLSKSNKQGDIQWWSKEIPEKNDSNCCWWYRPCSVNVEMSAYGLLATLEASSAGLEGLPIMK<br>WLVSQRNDKGGFESTQDTVVGLQALSKMAAQLSSSEADMSLKVIITGEQEKCLQVNGGNILVLQKHELAANTRKLEMIATG<br>TGCALFQLSYKYNIKDVDNSPRFTLKPEAKQGSIKSCIDLSITTSFIPKEDQAVSNMAVMEVDMPSGFIVESDTLKQLKQHEM<br>VKKVETKRSDTTVVLYFDNIGEEAVHLQMSAFQKHEVENAKPANVIIYDYYDNTRCARSFYEIAV
**AgTEP3**	MCHPLVVMPWYVRILVVISLLGSSWGVLVVGPKFVRSNQEYALVISNFNSGSSKVNLMLHMEGFSKNQTSVFAIRKPVDVR<br>RFMSRIVSFDIPNIASPVDIKLTMVGQRGFSFHEEEHLVHRSKSISGLIQIDKPVFRPGDLVKFRAIVLDTELKPPARIKSVNVTIQ<br>DPHQNKIRGWPAAKLYAGVFENDLQLAPAPLLGVWNITVQVGEEQLVFKTFEVKEYVLTSYDVQVMPSVMPLVEHQTLNL<br>TIVANYHFGKPVQGVAKVELYLVDDTLDQKKELTMYGMGQVELRFNELLELYEDQQDVRVKLTFTEQHTNRTVVKEQAIT<br>VYKHPYRAQLTKESPQFRPGTPFKCTLTLIYHDGRPAGHVPFFVNVEGEDVDHQQTYTTGRDGTIKLLMRPTELTETIDITVSE<br>DNSEFTYTERIEKVHADTNVFLKLELKSPIKLGKLIRLMVTCNERMTFFIYYVISKGNIVDAGFVRPNRQTKFMFQLTASEKMI<br>PKAYIFVATVSQDVVVWDSLEIDLKQFSNHLDIIIDEKELKPGQEIELLLKGRPSAYVGLAAYDKGLLAYSKQHDLFWEDVM<br>QVFDTFHATDQNEFDVFNSMGLFARLSGGNRIGASPTTTERFGSAASRPISRLVAYRTNFLESWLWQNVSIGRTGSRTVHEVL<br>PDTTTSWYLTGFSIDPVYGLGIIKKPIEFITVKPFYIVDSLPYSIKRGEAAVLQFTLFNNLEAEYIADVTLYNVANQTEFIERPDK<br>DLSYTKSVSVPPKVGVPISFGVKARKLGEMVVRIKASIMTGKETDAMEKVIRVIPENIMFEKTETRFFSMDEYGKQEFNMQLD<br>IPKNISTVQIKCRISSNLLSPVIHNLDSLLDVPSASGAPSMINFIPPLVVLDYLKAVSSTTTHLIEKATGLLRNGYQLELKYRQRD<br>GSFGNWRDSKGSVFVTALVGTSLEAASKHITEVDLTLVDRLFEWLAAKQHSSGRFDEEQPITYYSLQGGSRNGIALTSFVLIA<br>FLQNTKASAQHRSIIEKGIQYVANQLESIADVYDLSLATYALMLADHRQKSSALNKLIELGIATNETRYWPRDTASIETTAYA<br>LLSLVHAKRYADGLMVMHWLVNQQSATGSFPRTQDTFVGIRALAALSEAIAPQKNDYTAIVLHGKARKVYKVAASEADQE<br>YHDVLPGDSKLVRFSANGRGFGMFTVAFQYGIDVRNIEHGFSLRLVDQFSNEAYTLQLQVCTSFSPQLMHTRSNLALVEVNF<br>PSGYVVSRKSLVDETRRNPFKDVEVRYGQTSLVIYYETLGPEENCFSVTANRLFRVAFHRQAYVMVHDTYDEKFRAIKFYQV<br>PHDGADIQSYLD
**AgTEP4**	MRHLASASLLVITLLINFVHGILIVGPKHIRAGQNYTVILSSFESNTNERNLLLQLDGLSAGGKKLLHLGQPTRVQPLSNSVISF<br>PLPESLPAGSYKLTIDGQNGFKFHREADLLLAASTTTALVQLSKPIYKPGDVLQFRVIVLGGDLKPPAPSVTATVIVHDPQRNV<br>IRRWTAVSLQLGVFEEQLQIGTVPLLGRYTITVTVTGANEIVSKTFDVREYVLPAFEVAVKARAVPLEKHQRLNLTLSARYYT<br>GQPVRGVATVELYLEDDKLDQRRVVGVYGAIQLDLPFNEHLSVYDDSQDVRVHVAFTQDETNRTIYKEQRITVYKLPYRVE<br>LVKEQPEFRAKVPFDCQLRVRYQDGTPAKGAAFEVKVEGAYTTDRRVAYTSDAAGVIKLTLQPEASSESIDITVNAGSEELL<br>YERISKREASKAFLQVKLDSDVKIGKPFKLKVTCNEELSFFMYYVVSRGVIVDSAFLQPKKVTEHFIEIEASDQMVPRSKVIVV<br>TVAKNVVLCDFVDIDFEDLRNNFDLQIDRTEIRPGDQLQLNMRGPPGAFVALAAYDKSLLQYSNNHDIFWEDVWGVFDKFY<br>SVERNEFDLFHSLGLFARTMEHITFDKANDQTARDGSSSSKNGPSNSQASFRTNFLESWLWKTDKIGSSGSATTKESVPDTITA<br>WHLTGFSIDPVYGLGIIKQPLQLTTVQPFYIVPNMPYSIKRGELVELQFIVFNNFPKKYKASVTLFSVDNQTEFVGRPATETSYT<br>KSIEASPDTGVPVAFLIKARKLGEMTVRVDASIEPAKDSIESVIRVIPESLVKREMISRFFCHNTYQNQSFVLGLDFDRKADAGT<br>RKIDFILTPNILTSVMDNLESLLSVPTGCGEQNMMRLVPIVLVLDYLTSIGSADKQLTAKAIGLLRAGYQNQMRYRQPDGSFG<br>LWEKSGGAVFLTAFVGKTLATAAKYISEIEPSMVEQAFDWLAARQHSTGRFDEVGPVFHRDMQGGLRQGIALTSFVLIALLE<br>QPKVATKHRAAIEKGIDYVTQTLGSIEDSYDLAIATYALLLQKHSSGERFLEKLIGLSTVQQNGTERFWARDAHGIETTAYGL<br>LSFVLAEKYVDGTSIMRWLVKQRYTPGSFPRTQDTFVGLKALTKLAEKISPSRNDYSVQLRHAGRKKEFRVTSQDIGTLQNA<br>QQGVDETAQLELHVAGIGFGLLQVVYEYGVDLRNFTAQFVLELQKSVTNANHQLQLEVCSSFTPQLSDGRSNMVLVEVNFP<br>SGYTVEQRGQPITGATKHNPIQKTEVRFGATSVVVYYNSMGPERNCFTITAYRRQKVTLKRPAYVLVHDYYDPKLNAIKMY<br>QVDD
**AgTEP6**	MSKSIMWKCIRSRILMVIIVIGAAHGLLVVGPKFIRANQEYTLVISNFNSQLSKVDLLLKLEGETDNGLSVLNVTKMVDVRRN<br>MNRMINFNMPEDLTAGNYKITIDGQRGFSFHKEAELVYLSKSISGLIQVDKPVFKPGDTVNFRVIVLDTELKPPARVKSVYVTI<br>RDPQRNVIRKWSTAKLYAGVFESDLQIAPTPMLGVWNISVEVEGEELVSKTFEVKEYVLSTFDVQVMPSVIPLEEHQAVNLTI<br>EANYHFGKPVQGVAKVELYLDDDKLKLKKELTVYGKGQVELRFDNFAMDADQQDVPVKVSFVEQYTNRTVVKQSQITVY<br>RYAYRVELIKESPQFRPGLPFKCALQFTHHDGTPAKGISGKVEVSDVRFETTTTSDNDGLIKLELQPSEGTEQLSIHFNAVDGF<br>FFYEDVNKVETVTDAYIKLELKSPIKRNKLMRFMVTCTERMTFFVYYVMSKGNIIDAGFMRPNKQTKYLLQLNATEKMIPK<br>AKILVATLVNRTMVNDIVDIDFQGFRSNFDLSIDEQEIKPGRQIELSMSGRPGAYVGLAAYDKALLLFNKNHDLFWEDIGQVF<br>DGFHSYDTNEFDLFDNMGLFARMDNIMFDQSNDKSARSGQQADGTVFRKQFLESWLWKTAIIGNSGTLKLIEVVPDTTTTW<br>YLTGFSIDPVYGLGIVKKPIEITTVQPFVVMESLPYSIKRGEAIEIQFILISNLQEEYTVDVTLYNENNEMEFLGRSISNVSYTKSV<br>SVPPKVGKPVSFLVKAKKLGEMMVRVKASIANELATDALEKVIRVTPESLVQSGVESFGFFMDTHQNRTFLVNPNIDKKADN<br>GSIAIELRVNPNLLIKVKENLNDLRTVSPTCKNVIRLALNFAVADYLIACGPKEQNLPENAVDALSKEYMLLMTCLNSDSSFD<br>SVQNIVSNIFYTAFVANTLDTAPKNVCQTSNVKLEKAFDWLASRQQRFGSFREIETDLHYTRSEIALTSYVLASMLENESAKV<br>KHAVVIEKGMWFLSNQFDLITSANDLAIVTYAMMLYGHWRKDAAFEKLIDMSTITNNGAERYWKTSNSVEATSYALLSHVL<br>SNKLLAALPIMRWLVNQKSEIDSVSGQETTYLRLKALSRMTNKISPSRNDYMVQLKYKQSTRLLRFDSYRNMIQNITTPQDL<br>RKIKITVEGIGVGLLEVMYKYRLNLVNFEHRFQLDLQKQNTSSDNELRLKVCANYIPTLRDSHSNMTLIEVTLPSGYVVDRNP<br>ISEQTTVNPIQNMEIRYGGTSVVLYYYNMGTERNCFTVTAYRRFKVALKRPAYVVVYDYYDTNQNAIKVYEMDKQNVCEIC<br>DACGCTAECKT
**AgTEP9**	MWQLIRSRILTVIICIGAAHGLLFVGPKFIRDNHSYTLTISNFYSNPRKMYLMVKLEGQTDNGLSVLNITKMIDVRSNSIRMISF<br>CMPDNLSTGDYKITIDGQQGFNFHMETDLFYLKKTVAGLIQVDKPVFKPGDTVNFRVIVLDTELKPPARVKSVHVTIRDPQRS<br>VIRKWSTAKLYTGVFEGDLQIAPTPMLGVWNILVQVEGEELVSKTFEVKEYVLSMFDVQVMPSVIPLKKHQALNLTIEAYYH<br>FGKPVQGVAKVELYLDDDKLDQQKEITVYGIGQVELRFADYFDMYEDQQDVRVKVTFIEHYTNRTVVKQSQITVYRYAYR<br>VQLIKESPQFRPGLPFKCALQFTHHDGTPAKGITGKVEVTDVEFETTATSDNDGLIKLELHPSEGTEYLGVNFDSIDGFYYYEG<br>VSQIQTDAFIKLELKSPINLNKLMRFIVTCTERMTFFVYYVVSKSNIVDAGFVRPKNETTFLLQLYATEKLFPKAKMLVATVT<br>GRTVVYDYINLDFQVFHNNFTLSVDEQEIKPGRQIELSMSGRPGAYVGLAAYDKALLLFNQNHDLILDDFLKVFDGFHVHHE<br>GEFDQLHTMGLFARTLDDFLFQNYNHKSERNGQQMEQTVVRKQFVESWLWKNATIGSSGSLKLTEVVPDTTTSWYLTAFSI<br>DPVVGLGIIKKPIEFTTVQPFVIMESLPYSIKRGEAIEIQFILISSLQEEHTADVTLYNENNEMEFIGRSIANASYTKSVRVLPKVG<br>KPISFLVKAKKLGEMMVRVKASIANGIAADALEKVIQVTPESLVQSGVESFGFFMNTYQNRTFLVNPNIDKKADNGSVEIKLR<br>FNPNLLITVKDNLNDIRTDWSRCNEGIRGTLNFVVHDYLNTIGSSDQISSDDSAKIIIHFVRLQKCFISKAPWRNKVFDTAFLVN

	ALHNAMKYVYWNDKHKLEKIFAWLASQQHHSGSFKETESDLHYKRSEVALTSYVLAVMLENESAKVEHAVVIEKGMSFLS<br>NQLDLITSANDLAIVTYAMMLYGHRLRDAAFEKLIDMSTITNNGTERYWNTSNSVEATSFALLSYVVPNKLLEALPIMRWLV<br>NQKSELNSVSGQQNTYLRLKALSSIAKKISPSRNDLVAKLKYKQSTRLLRFDKYSNMIQNITTPQGVRKIEITVMGIGAGLLEV<br>IYQYSLNLMNFEHRFQLDVQKQNTSSNHELRLKVCASFIPTVSESRSNMALIEVTLPSGYEVDHNPISEQTTVNPIYHIEIRYGG<br>TSVVVYYKNMSNIRNCFTVSAYRRLKVALKRPAYVVVYDYYDTNLNAIKVYEVDKQNVCEICEEENCPAECKI
**AgTEP10**	MWQFISSRILTVIVFIGAANGLLFVGPKYIRDNQNYTLTISNFYSNPSKMDLMVTLEGQIDNGLSVLNVTRMIDVQRNSIRMIS<br>FSIPDNLSSGNYKITIDGRQGFNFHMETELLYLKKSVAGLIQIDKPIFNPGDKVNFRVIVLDTELKPPARVKSVHVTIRDPQRNV<br>IRKWSTAKLYAGVFEGDLQIAPTPMFGVWNILVQVEGEELVSKTFEVKEYVLSTFAVQVMPSVIPLEEHQALNLTIEANDYFG<br>KPVQGVAKVELYLDDDMIDQKKELTVYGTGQVELLFFDKFEMYVDQQDVRVKVTFIEHYTNRTVVKQSQITVYKHAYRVQ<br>LIKGNPHFHPGLPFKCVLQFTHHDGTPATGITGKVEVSGIGFGTTVTSDNDGLVKLELQPSEGIESIDVSFLNNQNGLIFEETMY<br>KEESYTNAYIKLELKSSINLYKLMRFMVTCTERTTFFVYYVVSKGNIIDADFIRLNKEMTYHLQLNATEKMIPKAKILIATVVG<br>RTVVYDCMDLDFQEFRNNFDLSVDEQEIKPGRQIELSMSGRPGAYVGLAAYDKALLLFNQNHDLFWEDFLKVFDGFHSYRE<br>NDYDLFHSMGLFAKTLDDILFENSNHKSERSGQQMEQTVVRKQFVESWLWKNVTIGSSGSLKLTEVVPDTMTSWYLTGFSI<br>DPVVGLGIINNPIEFTTVQPFFILECLPYSIKRDEASEIQFIVVSNLQEGHTVDVTLYNENNEMEIIGRSIANVNYTKSVHVLPKV<br>GKPISFLVKAKKLGEMMVRVKASISNGIATDALEKVIRVMPESLVQSRVESFGFFMDTHQNQTFLVNPSIDKKANKGSVKFEL<br>RVNPNLLITVQENLDNIRADISGCSESIKDMLHFVVHDYLVAIGSTHPYESNDNKCIKSNGSWRNQVFETAFLVNALQDAAK<br>YIYFTDRLKREKAFDWLASQQHHSGSFKETETDLHYKRSDVALTSYVLAVMLENESAKVQHAVVIEKGMSFLSDQLDLITSA<br>NDLAIVTYAMMLYGHRLKDAAFEKLIDVSTITNNGAERYWNTSNSVEATSYALLSHVVANKLLAALPIMRWLVNQRSDLDS<br>VAGQENTYLRLKALSRMTNKISPSRNDYTVQLKYKQSTRLLRFDKYSNMIQNITTPQGVRKIEITVMGIGAGLLEVIYQYSLN<br>LTNFENRFQLDLQKQKTSSDHELRLKVCVSFIPTVSESRSNMALIEVTLPSGYVVDHNPISEQTTVNPIDHFEIRYGGASVVVY<br>YKNMSNVRNCFTVTAYRRFKVALKRPAYVVVYDYYDTNKNAIKMYEVDKQNVCEICEEDDCPAECQK
**AgTEP13**	MKASKHPTTRGSDARTTPLLLLLCLFIASQANLLVQAQQSQPQSNDVNRYNPQDPFGGRQQYNPQYPSSQYDPNTRTNQGLP<br>PSQSSGTNYQRDYYGNNAVNGNNQDSNSIDSNRFGQDNRNVVLQSTTPRDYFNRQTTPPFRTRTSNRNPFSNNDYAAGNSFS<br>QGITYFIVASKMVRPGQVYKVAVSVLESHLPLTVRTSISRDGVELSSETKPITVGVPETMLMRVPPTSVVGEYKLRVEGSYDK<br>NFGGYVFANETKLIFSQRSMTIFVQTDKPVYMQGETVRFRAIPITTELKGFDKEMNVYMLDPAGHIMRRWLSRQSNQGSVSL<br>QYQLSDQPVFGEWKIRIEAQGQIEEARFNVEEYYQTRFEVNVTMPAFFFNTDRYIYGRIMANFTNGTPVKGNLTLKATIRPIGF<br>FNPEAINQMNRVGNLGRLTNLNQNVPYYLQKTNPDLLNQYNPQNSFSSQIGGRDQGGYDGVGQGYDGRYSSNNFQDSYVIE<br>RHLNFDEEWPFWIKKPVESDSQWDSWTNTYRDSLPYLRFFNGTYNFRFPMAELAQLVPNLSGMEILFTARVGERYYNEIVEG<br>YSLTRVYNSSIRIAFLGDSPQVFKPSMPFTVYLIAEYHDGSPLPLDEFNAGRMEVSGTIDSRASGGRNSFDSRVLHMSQKAGV<br>WELKLDIRHDLNLENTKQTNEFLNEIQSMRLVANYIDPSGERASTELLLLSHFSPNNHNIKVHTSTADAKVGEYITLHVQSNF<br>YIKDFNYLVMSKGIILVTGHENMKGGVKTMAITLSAEMAPAATIVVWHIGRYGKLLTDSLTFPVNGISRNNFTVFINNRKART<br>GEKVEVAIYGEPGSYVGLSAIDNAFYTMQAGNELTYANVIQKMLSFDEHTNGTFKKTWMSHDGDPDELVYYPASTFGIDAN<br>RTFDYAGLVVFTDGVVPMRPTICDAALNYSECLNGRCYRTDKRCDGYMDCEDGTDEAGCSTRNETLLVEFRKFRFNRILRH<br>YQNVWLWKDVNIGPHGRYIFNLDVPQVPALWSVSAFGISGTRGYGMIRKPIEYVGIQPFFINVEMPTACHQGEQVGIRVAVF<br>NYQTVDIEATVVLHSSPDYQFVHVEEDGIVRSYNPRTSFGEHQFYIYLNAQDSTNVYLPIVPTRLGEIEVTIHASTLLGAHQISR<br>KITVEADGLPQYRHQSILLDLRNRAYLVQFMHVNVTETPIIPYEIDRYYVFGSNRARISVVGDVVGAIFPTMPINTTSLLELPM<br>DAAEQNMFSFAANFYTIKYMRASTLRDKRTEKQAFHFMNILYQRQLSYLMEDGGFSLFRADWNQSAPSVWLTAYCAQVFG<br>EMAGLYEYENFIFIDPYMIQKNMHWLLRHQKEDGSFWEETWLPDRKANSSAFFSRNDVVREKNVTLTAHVLITLTTVNLPA<br>GRLAARVALSQQRALQYLQRNLATIKESGSTYEIAIVAYALMIAKAPKAEAAFTMLSAKMRSIGEFNYWGEEEVPLPPTKLE<br>NQRYFSLPRLPYKYDSLNIQTTAYALLTYVSRQEIHVDPIVAWLNAQRLTDGGWASSQDTGMAMKALTEYSTRNRVSNVTQ<br>LAIKVEATSLPGETKQLYITRKSLAQQQFLDVPNAWGTVRVEATGVGYAILQMHVQYSVDTYKFQTQPPVPAFDLTTRTIFH<br>GRNQSHISYVICQRWINTQESIRSGMAVLDVAVPTGYMIQQQKLDSYILSQRVRNLQRARFQERKVLFYFDYLDNDYVCVNF<br>TLERWMPVANMSRYLPIRVYDYYAPERFNETIFDSLQTYLLNICEVCGSSQCPYCSIYNIAVRFPGSIVLMLLTGIVLVARHYR<br>IVNPNGWIFLND
**AgTEP15**	MRRSTMFASKKDPAIRILSTSSSSLLAVCLVLSVALVPAVQCEGHYSIVGAKLLRPNSEYHVAVTNQDVSEPIRFSLAITDASS<br>VIAKQEITLNTGETRLVPFAIGDISESSYKLVAEGLSGLTFKNETDLEYQQKSFSVFVQTDKSIYKPGDTVRFRVLVLDPNTKPL<br>QKADNISVHINDAKANRIKQWKEGKLVKGVFESELTLSTAPVLGAWTINVEVLGSKHNKVFEVDEYVLPKFEVTVESPGITT<br>FKDGKVKAIIRSKYTYGKPVKGEATVSVSPEFQFHYVQPFAKDVITRKVIPIDGKGSVEFDLREDIHLEGDYSRNIVIEAVVEE<br>ELTGRKQNASAKVMIYDRRYKMELVKSDDNFKPGLPYTAWLKVSYQDGAPVQDQTNPVEVKQSSFESTTSVQNYTLDQNG<br>MAKLEINTEVNSSYINVVGVYLGQEFYLHGISKAESDVDSYIRAQVLTEMPLVGKDVLVEVTSTSPMKYFTYQLLGRGDVLL<br>SNTIAVPESKTQSFKFPATFAMVPRAKLVVYYIAPNGDMVSDSKVITFDSELQNFMKVSLSKEQSKPGQDVEISISTNPDSYVG<br>LLGVDQSVLLLKSGNDITKQQVFSELEKYEERSYGFYRRKKRFAWNPHAEHRDFSTVGAFVMSNANDPPQLAFPEAIAFASP<br>SLGASAGIPSADGPTVRKDFPESWIWYSAAEEGFSGEKTLQKKVPDTITSWIITGFSVNPIYGLGLTQQPRKLNVFLPFFVSTNL<br>PYSVKRGEVVAIPIVVFNYMEDDQTAEVVLHNDEQEFEFADVENEVVESNKVELFRQKRLDIASNTGKSVSFMVKPKKLGHI<br>TIKVTAKTKIAGDAVERQLLVEPEGLPQFINKAAFIDLRAAPELTKTFEVEIPKNAVPDSTRIEVAVIGDVMGSTIQNLDSLIRM<br>PYGCGEQNMLNFVPNIVVLDYLKATNKLTANIEAKAKKFMEAGYQRELGYKHRDGSFSAFGENDKSGSTWLTAFVARSFK<br>QAANHITIDEGVIDKSLEWLSDHQAPNGSFPEVGVVSHKDMQGGSGSGVALTAYTLIAFLENINLVDKYKNTINKAIDYVYR<br>NTESLDDTYALALAAYALQLADHSSKGLILSKLDTKATTDSDSKWWHKPIPETEQKNPWYSRPNSVNVEMSAYGMLAFLEA<br>GLDTDALPIMKWLIGQRNDKGGFQSTQDTVVGLQALAKLAAKITSPNNDVTLTAKINENQEKRMTVNAENGMILQKFELPS<br>AARNIEIQATGSGFAVVQLSYKYNMNVTGEWPRFVLDPQVNANTNPDYLHLSVCASFVPSAGQNVSNMAVMEVGFPSGFT<br>ADSDTLPSLENMPFIKKVETKDGDTTVVLYFDSLDQRELCPTISAFRTHKVAKQKPAPVVIYDYYDNSRIARQFYDGPKASLC<br>DICENEDCGEACSIRSQKQRSSDSPSRQPTVEGTMQSGSQTVSVSFFTFLLATLLVRMFH
**AaMCR**	MTRRWHPTWSGVLLLLFGFPSILTWAQQQQNPNDVPRYNPLDPFNRQRYNPTSSNFQPQSNPTASNDPRNPSFGSRDFYGTD<br>SNSIDNRFDPSRGDRDRDRDVLFQSTTPREFPGTRFGTTPRPRTFNPQSNRNVFLDQSNNPLNKEITYFVVASRMVRPGQIYKV<br>SVNLLEAQHPMAVRASISRDGVELSSELKSVRVGIPETLLMRIPPTSVVGDYKLRVEGSYENTFGGYIFVNETKLTFSQRSMTI<br>FIQTDKPVYMQGEMVRFRTIPITTELKGFDNAVDVYMLDPTGHIMRRWLSRQSNLGSVSLEYKLSDQPMFGEWRIRVIAQGQ<br>IEEGKFSVEEYYQTRFEVNVTMPAFFFQTDPFIHGKIMANFTNGTPTRGNLTLKATIRPIGWMNPKAINHMNRVWNTGNRRE<br>LDPNIPYYLQYTNPDLFNAQSTFTSQLNQPDSQYQDQRYQSNYQDSYVIERHFNFDEEWPFWIKKPIDSAEQWDSWTNTYRE<br>SLPYLRYFNGTYHFKFPMSELSQLFQSNLAGMEVLITARVGERFYDEVIEGYAMTRIYNSSIRVAFMGNSPQVFKPSMPFTVY<br>LIAEYHDGSPLPIDPLYPGRMELSGTIDSRSGGGRNTYDVKELQMSDKPGVWELKIDLRNDLNLENSKQTNEFLNQIQSMRLS<br>ANYIHPSGERASAELLLLSHFSPNNHNIKVLTSTRDAKVGEYMIFHVQSNFYIKDFHYIVMSKGIVLVTGQEIISGGVRTLSITL<br>SAEMAPTATIVVWHIGRYGKVIADSLTFPVNGISRNNFTVFINNRKARTGEKVEVAIYGEAGSYVGLSGIDNAFYTMQAGNE<br>LTYANVITKMANFDEQTNGTFKQTWISHEGDPDELVYYPSSTFGIDANRTFEYVGLVVFTDGVIPRRPLSCDPAMNYSECLSG<br>RCYRSDKRCDGYFDCEDGTDEAGCDNRNSTALAEFRKYRFNRILRHYQNVWLWKDINIGPHGRFIFNLEVPKIPALWIVSAF<br>GVSTSQGFGMLRKPLEYVGVQPFFINLEMPTVCRQGEQVGIRVAVFNYQTVDIEVTVVLHSSPDYQFVHVEEDGIVRSYNPR<br>TSFGEHQFYIYIYAQDSSNVYIPIVPTRLGDIEVTIHASTLLGAYQVSRKINVEPDGLVQHRHQSTLLDLSNRAYFFQYMHVNV<br>TETPIIPYEIDRYYVFGSNKARISVVGDVVGAIFPTMPVNATSLLSLPMDSAEQNMFSFAANFYTIQYMRAIKQRNKKTEKLAF<br>HYMNIGYQKQLSYLNDDGSFSLFRADWNQSASSVWLTAYCARIFSEASFYEYENFIYIDPLVIQKNIHYILQHQKDDGSFWEV<br>TWLPDRKVNQSSEHPRNEIVRSKNITLTAHVLITLVSVKDLSGRLGSSVALAEQRASRYIERNIGLIKDYGSPYEIAVVSYALA<br>QAKAPQAEHAFKILASKMRSIADLNYWGNDEVPQPPSKLENQKYFNLPRLPYKYDSVNIETTAYALLTYVSRQETFTVEPIVR<br>WLNSQRLMDGGWASSQDTGIAMKALTEYSTRNRVADVTSLTVTVEATSLPGESKVLHIGQHNLATIQDIEIPNAWGTVKVQ<br>AKGVGYAILQMHVEYSVDTYKFQTMPPVKSFDLTTHTVFHGRNQSHISYVICQRWTNTAESIRSGMAVLDVAVPTGYMIQQ<br>QKLDSYTISQRVRNLQRARYQERKVLFYFDYLDNDFVCVNFTLERWMPVANMSRYLPIRVYDYYAPERFNETIFDSLQTYLL<br>NICEVCGSSQCPYCRIYNTAIRNPVSVLLLLFTSVLIVARHYRIVNPNSWIFWND
**AaTEP3**	MGHLTMSPPWALSLMVLISVFVVSCKCEGYYTVVGSKLLRPNSEYHVSVSNLNVREPLRFRITLNNTRTGTPLASEDLSLGQ<br>GESRLVPFSIGDITEGDYSLTAEGLSGFIFKNESSLTFQSKSFSVFVQTDKAVYKPGDTVRFRVLVLDPNTKPLQKIDTVKVHIT

	DGKNNRIKQWNDAKLVKGVFESELALSSAPVLGNWKINVEALGTKKTQEFEVDEYVLPKFEVSVESPGITTLKDGKVKAIVR<br>AKYTYGKSVKGEATVSAYPDFNFHYVQPFERDVITRKTVPIDGKGSVEFDLRDEIKLQGDYTRNIVIEAVVEEELTGRKQNSS<br>SKVKIYDRAYNMELVKSSEKFKPGLPFTAWLKATFQDGAPLQDDTNSVKVTQEFGWPNQNTTEHTYKLDKNGMAKLIIDTE<br>TSSDSISFRAEYLGATFYLGSISQGWVKYSAYVRAKVLTETPTINKDVTVDVSSTVPMKYFNYQVLGRGDVLIGGTIAVPDR<br>MSHTFRFPASFAMVPRARLLVYFIHSDGQMISDFAEIEFASELQNFLKVQLSKTESKPGQDVDITISTNPDSYVGLLGVDQSVL<br>LLKSGNDLTTGKVFDDLKTYEEAVYLQYRRKRFAPWRRFNYYDDFNDAGAVIMTNANEQPAIPPWASYASFDGGVIDIRTY<br>ENEDPFAVPLAASAGVMPPSGVGGAELSVRKSFPESWIWQTFSNDSFSGEKTITKKVPDTITSWMITGFSVSPVYGLGLTRQP<br>RKLNVFLPFFVSTNLPYSVKRGEVVSIPIVVFNYMDSDQTAEVTFHNTEQEFEFADVENEVHENPKLELFRKKTVQVASNTGK<br>TIPFMIKPSKLGHITIKVTATTQLAGDGVERQLLVEPEGLPQYVNKASFVDLRATPEVNNNFTVEIPKNAVPDSTRIEIAVIGDV<br>LGSTIQNLDSLIRMPYGCGEQNMLNFVPNIVVLDYLKNTNQLTASIEDKAKKYMESGYQRELTYMHDDGSFSAFGKSDPKGS<br>TWLTAFVARSFKQAADHISVDEKIIDKSLEWLSDQQSSNGSFPEVGKVSHTDMQGGSGQGIALTAYTLIAFLENKNLIPKYTN<br>VINKALDYIVRNTEGLDDNYALAIAAYALQLADHSAKDFTLSQLDTKATTDEDQKWWNKPIPEADKKNPWYSKPNSVNVE<br>MSAYGMLAFMEAGLDSDALPIMKWLISQRNDKGGFQSTQDTVVGLQALAKLAAKISSKNNDVTIVVTYNENQQKEMKINS<br>ENNMILQKFELPSSAKDIDIKATGRGFAVVSLGYKYNMNVTGEWPRFVLDPQVNKNSNQDYLHLTVCTNFVPTAGQNKSN<br>MAVMEVGFPSGFTADSDTLPSLENTEYIKKVELKDGDTVVVMYFDSLDRNELCPTVSAFRTHKVAKQKPAPVVIYDYYDNS<br>RIARQFYNGPAAGLCDICENEDCGNSCSIKSQKQRSPKEEATVMAKSGAVGITIGLQLIVAALLVRLFN
**AaTEP20**	MGMEKRSRTVPARRRRRWSIQANMWILPLLICCGMLIERVQSQGFSVIGPKTIRPYSTYSVAFSNSVNKNVHLNVILEDQNNP<br>SEFSLKKLAVVNRRTVKKIDFDIGNITSSEYQLLVQSADRTFSFDQRVELLYEPKTMSVFIQTDKPVYTPGDLLRFRVIVVDAD<br>TRPVTSIKTVNIAIDDSAENSIRKWPYAKLLNGVFESQVQLASSPVLGTWSITVKASDDIIVTKQIEVKEYVLPKFFVKVYPSEV<br>LLVQNEKVSLTLEAYYTFKEPVDGNYKVELFLDHTKRKPDFIKSDRITGKTSLEFQLKNEVDIDGDEQYTDVTVKVEVVETFS<br>NRTVSITEKIPIYRQPYAVTLLPSAPSFRPGVPFDVKIFVKDQLGHPPPEENTASIDLTVEFHLPTDSDTKSLTVDLDEKGTGQFI<br>LLPHPNAQELKVTATYDSQEYEVIHDPIHGFSSQSKQYIKVTLNPKYYNNIKVDKDIVLDISCTETMTHFSYIVVTKGNIVEAS<br>NVPVAKKKKHSLRLKMTSKMSPESRLLVYYTNREYLIFDDIELKFDSFNNDFKFDLNDDEYFPGQSVYIDVYASKDSYVAFS<br>GIDESVLLVGKERHDFNKGDVLKELALYGATNDAEFDLFHKYGLFLKSTATVDTPVTRSQNARFGTLLGRTKQAIEIRTQFLE<br>SWLWKSFSMDGRNNFKAIEDSVPDTITTYHVSGFALSPTLGLGVIQQPVSFTVRKKFYLVANLPYSIKRGEVALIQVTVFNFL<br>GSSVTTDVTLFNKRDEIEFVEKASTNNTHRTKAVIAPNNNGKPVSFMVKAKKLGQIAIKFQAENLLETDALEHMLRVTPESH<br>RYEKNVARFIQLPTHSKVPFDVKLDIPKNIDEGSAQIKFTLDPDILGTTISNLDGLIRKPSGCGEQNMLHFVPNIVVLDYLNETN<br>TAAEDVRTKAINFLSSGYQNQLRYKRSDGAFSVWGQSHAGSTFLTAFVAKSFKIAAKYIQVDKSIVDAAFDWLAKQQQSDG<br>RFPEVGQVIHADMQGGLRNNGFALTAYVLIAFAENEEVYRKYRSQLIKTTSYIANNLDNMENPYDLSLSTYALMLANHGKR<br>TEFLDKLVEISIFDSNQTERYWDSKPVDIEVAGYALLSYVAAGDLLHATPIMRWLNKQRYGLGGFPGTQDTFVGLKALATFA<br>AKVSSGRNDYRVTIIHEPNRRRTFDVDRHNAFNIQELDIPNNIRKMRVEVVGIGNGYFQVAYQYYQNIQVAKPSFSLTIDQLN<br>TTTEHMQQLDVCVKYIPKEAYQKSNMALVEIFLPSGLVADSDAITDKTGGIRRIERRFSDTSVVIYYDNLGPEDECFRVTAYR<br>RYKIALHLPSYIIVYDYYNSERFAIQQYEGKVLQLCDICEDEDCETLSCENSSK
**AaTEP22**	MIMNSVIVLVCSFIVVAKCQSSSVLVIGPKYIRPRHPFNVAFANSLNSNVNLKLTLQCDGDNGFVKNASLRLNYQSAKSFSVD<br>VPNITSGTDCTFSAVNDGGSVMVDHMANLLLPAKTLSVFIITDKPVYKPGDILRFRVVVVDIATKPVKHMESIVIEIIDSDGAF<br>KREWLQARLLNGVFEAAYRLPSVPALGIWNITATANDNGFENTKVQDFEVREYVIPKVVLKVVPSRTLLIAEKEISLDVKAH<br>YTFGEPVEGKLRVDLFTNPLFRRATHSVEKSFNTNVQIKFKLDRELALRDANFCMVSANVSLTEKLSNVTTVVTLEFPVFRHP<br>YKIELINPAMNYRPGVGYTCKLSVKDHFGIPVDAQGNSITVQSDTDSVTGQLDRQGMVTLTLPMPDSEETVLISVVYENVEY<br>NKIIEVEASEDLSTQYLHISTKTRIIVGNSITFTVNSNQHFTHMSYFVTNWGGILLAGHQKFSRKKTNTIRFKLTAAMSPYSRLL<br>VYTISGGQLIMDYFELDFEFFGNEFEFMLDDANYRPDQDIYVDVKAEKNSYLAFQAIDQGALLLGFDEFGLTRKQVQEDLAS<br>YVDADEENRLDLIHSFGLFLRSGFNETKTQSSRAKRFAPERRDRPYRNAIRLRTDFSESWFWKNSTMKNQKTQTFHDVVPDSI<br>TSWYVTGFALSPTLGLGLMHAPRKFTVTKPFYMVANLPYSIKRGEVVRIQIMLFNFLNSDLTTDVTLFNKNDEIDFVDRLSNN<br>PHFRTKAIIAPHNQVKSVSFLIKAKKLGEIAIKIEAVNQLESDGLEHMLRVTPESRLYEKTEARFIDLPTRNKTNFPITCNIPRDA<br>DPGSTKIEVFIDPPLMGFLAQNTTDSLLKIPTGASNLNLLTFVPNIVLLEYLKETGKVTPSIEQARNYVSSGYKNQLKYKHSNG<br>AFGQWNPPRGNPSVFLTALVANALATASKHIDIDKKIVEQAYSWISGKQKPNGCFEDDGEVIYTPLQNNSSSFALTAFIVSAIM<br>ENENIARQFAAVVQKATNCLAGNFDSLNNLHDIALTTYALALVRHEKRQIYLDRLIRDSIFEKGSSTERYWNEELNVEIAAYA<br>LLSYLQIGNVIDTMPIMTWLNKQRYSTGVFSGVQRTFVALKALGKMATYLNTSKNDYSVYISYDKHKSKFDVLSTKSLETFH<br>HELPDSVRNVNFAVEGIGFGYLQLTYQYHRNIQNAKASFMLDVTVLDSSNYNVQDLRVCLSYKPKEKYTVSGVALVEVYLP<br>SGLIVKESAVRDISRQIQRTERAFDNTAMFVYFNGLDTNSVCFDITAHRKFKIAMHRPSYVVVYDTNDMSGFAIKSYEGKVL<br>QICDICDDEDCRSMSC
**AaTEP23**	MCPLLIATLYFILQYTAPANSSEVGHYIILVPTVIHDHSKVSIGLSTVGYEENKQFQVILKKEQYDDKEGEVVNEKHRQSSDTR<br>LVQFTVSSLDNEPYQFLIHDPDGSVQTTHVEVSKRPVFIFIQTDKPLYKPGDTVKFRVLVLNHLTRPIDSLKVINVQLKDSNSD<br>LIRQWHYARLQKGVFQSQIDLANFVPLGNWTLKVSALNGIEETKTFNVAEYVLPLHEIHITASKQVQMNDEILKLIIDAKYTF<br>GKPIIGYVTLAFNELNYPDMYEINGRAVISIPLSDIVDTDVGDDVYHLNVQVKIEEDESEQIYEASETIPIHKRSYKITLRKSSEY<br>LLEEESIWCWLTITNPDGMPLTNRNVMTVKVTEMLGSRSNKHYSFQKEPDDEGVVSLKIDSSNTTDRLELEVTYEGETTKFEI<br>LHGSDNNNTRNDFIKASLLVQKPMLKHPVEVLVQSSFRMNLVIYYVISQGEILASGRISVNFKRTATFSYLATFPMVPEASLV<br>VFTINDAMLWKDIVRFKVHELDNFVDIEISSNSAEPRAQIFLEVKSNPGSTIGLLAVDRSLLQLGTGNDITQQIVLEKLGKPKID<br>EDVELERLGFTVLTNAKSHFMASPLGSSPHLLTQRFGDDEELHLGQDMQADHGTRILRKNFPETWLWTEMIEVDDKGHVDI<br>TDIIPDTMTSWSISAFAINTNHGLGVVKNPVALTVLKPFFVTVNLPYSIVKTEQAVVEVFVHNYLNQAQHVTVRVQNAQKDFI<br>HSDLEVSKTVFAPSNSVKTVTFALKPKRSGNLTVTILADCSLATDAIQRNLRVTPGGLQYFENSARFIEVQNSSMSFDPIKLVIP<br>RTATYGSVSITFSVEGFLLGAALTNLDHIIRLPSGCGEQNMLNLVPSVIALEYMDNTDTLTYGIKAKAIDYLQKGYQNQLKYK<br>LRDGSFSVFGQSDGRGSVFLTALVAKVFTFAKRHITIDNYVIEKAFNWLRERQLSDGRFVESGRIYYKELQSGVQDGTTLTAY<br>TLIAFLEHKSLTQKYLSVVNKGTEYIAKTYRFDGNPYSLALIAYVLQLAGHPRKHYFFDKLVELSKINEDRTMRWWGSGSTSI<br>ETTAYVLLTYMSRGSYIDAKSIMRWMVSQRYDKGGYSNTQNTFVGLQALGKYSRVMSLSNQNYDVFVNYDSERQRLHMN<br>STTSLVGHKFNIPSDVRQVKVDVEGTGAGVFQIAHKYNNIAHDSVPRFKIEKTLFKIRSDGVANMTIQAIYRPKKDFEETNMV<br>MIEVMFPNGYVVTNSQLKQLEKNNQIRRSETADGDTRLILYLDPMRPNNPVYVDVEAFRKSTVLNQAPGWIKVYDYYDPTR<br>EAIEYFDPISN
**AaTEP24**	MVKYGECTFLCALLSVVYTQSQTTGNYVILAPKYLRAKHPYQLSIATHDFNGFTKLHLAIDGYTENDEVANVIKEVHLKRSQ<br>TQLVEIDTTTLPPGSFSLNILSGSGGEVNLTIPLTVLDKAYTVLIQTNKPIYKPGNVIKFRVLLLDEATKPVHKPKAIHVTLADP<br>DGNEIKVWPYAMLQNGVFQSQLEISNEPNLGNWSITANAYGKDHTFSFLVDDYKLPKYELKVSTPQPATVSDREFSVDVEAR<br>YIFGRPVKGNATITVNGPKKQSKTAKINGKIRLSFSMIDLLHQTSIVDEIIPVEVIVVIHDQYTRENIKATNMFRIFNQSYRLSLK<br>KSSKYFIPGHPYRCIVEIKDQNGRRLTSIENHQALVNVVYSGPNGYHNTSEMRLNPESDGTVPLTLEVPEQVTHIELNISYRDT<br>NEHFELQRIHFSTTGQIQASIITEQLTLNTPIMVQVQSSIALNHLTYQIIAKGKIQTVQQMRFDDTDSVQFNITATPEMIPNAKVF<br>VFSMHNGLILKDTVPLMISSLPNWVNVTLPNEKVQPGSRIQLEVESTPNSLVGLLAVDRGAWLLGDGNQITKQSVLDEIGTFS<br>DEIEGDNDDISEMELITNGIIPTALTARFGNFVPDEEDAYIPPRKEFPESWLWLDLQKTGMDGKLHIADIVPDSITNWEITAFSIS<br>PEHGLGVQDEPVALAVSKPFFITINVPNSIKKSEVAIVKVAIFSVLNDTSYVGVTLKNSRQEFEFVDNRGRKDVSYQAKNVIVT<br>ANSATTVLFKIKPKKMGNIVIKVIAETTEASDSTEQLLRVTPESLPYSITEKRLIQLQNNRQSFELELKIPRHIDVNSEEIHFSVQG<br>NLLGDSVDGLDEMIRMPTGSGEQNVLKMVPNLVLLDYMVGVGKVNVPLRNRAIKFLGMGYQNQLKFKRNDGSFSMFGQQ<br>DNAGSVFLTALVAKTLHQASQFITVDEKVIENAYDWLRQQQKMDGSFTELGNIPEYGLQRKHTEKSVLTAYTLVAFLENDRI<br>SEKHKSIIDKGTQYLVSKIQGLESSYALALTAYALQLAQYKQEYAFSKLLENSQSNNGFRWWNDDNTAEATAYALLCYIQR<br>GDFVDPLPILRWLISKRHLFGPDNIETTFIGLQAIAEHSKRISPNRNNYEVSISRESNQLVTLNINPETSLTVQNVSLPSNVRKVR<br>VMVNGTGTGTFSIHYRYKTNILNLRPRFDVKVQTLDTTTSHYLDLKICAKFKPSEAYEISKLALMEITFPSGYIALDESVEELE<br>KMDTIRKITTKYDDASLWLYFESLPEHFLCIPVTSFRQSDVLQQIPGSVRVYDFFDDSRVAITHFDGKQLDKCEICDNDCLPEC<br>GN

**DmTEP1**	MLWLILSSTILHCVLLSNANGLYSVLAPKTLRSNSAYNVVVAIHNTTRTTEVSVSLTGPSLNSRKYVDVQSMSSKSVRFDIPK<br>LTEGDYELKVMGSGGIEFQNSTKLSFAPDLNWLYIQSDKATYKPGDKIQFRVLFLDKNTRPAVIDKPIKIEIRDGDQNLIKSWK<br>DIKPAKGVYSGELQLSDRPVLGNWTVTATVQDEGKVTNVLVVDKYVVPKFEVVVLTAKNVAASAGYIRATIKARYTFKKP<br>VKGHVVATIEGSSTEQSLPIDGEVNVEFPISATAKRLLKITAIVTEELTDIKHNGTAYVTVHQHRHKLEDLFWPTHYRPGVSSE<br>FKTVVRNLDGSPVMDSSKMVNFNVLCCQVSKNFSASLQNSIATEHIMLPETCQSCLVTSTFDTAENIERYIYKLNKPLMIAINT<br>KKPQLRKLLKINIISDTYLPYFILTVVARGNIVLSLFQEMKEKKKSQEIEFEPTFALVPQATIFVHYIIDGVLMSDEKTVDIERDF<br>ENTIEILTTNEALPRDEVSLKVKTNPHSFVGLLGVDQSVLLLRSGNDLNRDLILNNLATYSTDLVILTNANINIYRSSGGCYTNP<br>GYTNCTGSLIGRTMFKNEPTKNSGPVPIVGSTRAQASLPPVRKLFPETWLFSNITDVGANGEYIIKETVPDTLTSWVITGFSLSP<br>QSGLAVTRNPSRIRVFQPFFITTNLPYSVKRGEVIAIPVIVFNYLGMDVKAKVLMDNSDGQYEFIETTNKNVSQYLRGVRRKK<br>TLWIPANTGRGISFMIRPKKVGLTTLKITAISKYAGDRLHQILKVEADGVQKYVNKAVLINVQRLNRRSLAPPEKTIIIEKADN<br>VIEGSETVEFEVCGTSQAPQLEHLDDLVHLPCGCGEQNMFNFVPSILALSYLKAKNRQDQEIENKAKRYVETGYQIELNYKR<br>NDGSFSAWGQHDALGSTWLTAYVIRSFHQAAKYIDIDKNVLVAGLDFLVSRQSTDGKFKELGMVIHNSHGSPLALTSFVLLT<br>FFENEEYMPKYKHVIDRAVEFVVTEVHQSNEPYDLAIAALALSLARNRNAYKVLDKLDKLATRRGDHKWWTGSDKCKSSE<br>VETTSYVLLALLEHNISDEPKPIVDWLISKRNSNGGFVSSQDTVVGIMALTKYELQSHASTEAIDIEFWHLNEDKKHVRVTKE<br>NEFKVQTHQLPENTNEVKLLAKGQGRAQVQLTYRYNVATKEARPSFKLTTTVKKSHKGRLILGICGTYTPIAASERNKTTNM<br>ALMQVQLPSGYVCDIEPFADIEAISDVKRVETKNEDTEVHIYFEKLSPGDRKCLTLEAIYTHAVANLKPSWVRLYDYYATERS<br>ATEFYHVDTSLCDICHGNECGNMC
**DmTEP2**	MFRIFLTGIILQYALLVNATGIYSVVGPGTLRSNSKYNVVVSVHKADGPSQIKVSLNGPSYNETKQIELPPMSTQNVEFEVPKL<br>ATGNYNLSAEGVSGVVFKNSTKLNYADKKPSVFVQTDKATYKPADLVQFRILFLDENTRPAKIEKPISVIIIDGAQNRIKQLSD<br>VKLTKGVFSGELQLSEQPVLGTWKISVSVDGDNRETKSFEVDKYVLPKFEVIVDTPKAVVIADKVIKATIRAKYTYGKPVKG<br>KATVSMERSYGYFGDLNANGNKQEKTIDVDGKGHVEFDIIHWAQRGQYLPPIKLFAVVTEELTGNKQNATATVVLHQQRYS<br>IEPYERPEHFEANKSFIYQVVVKNVDGSPVTNSAKNVKIGFDKSYSYFHEPSPKTRINFEAPVNENGIATFNVRLPDSDSRYYRI<br>FASFDGSENTIGSISKFEPTPMSREPLKIQVNTKKPRLGEQVSFDVVSIEDLPYFVYTIVARGNVILSDYVDVPDGQKTYTVKFT<br>PTFSMVPKATIYVYYVVNNDLQFEEKTIDFEKEFSNSIDVSAPTNAKPSEEVKLRIKTDADSFVGLLGVDQSVLLLKSGNDLSQ<br>DDIFNSLNIYQTSTPWMNGYGRYPGQTSGLVTLTNANYPYNTGPLVMSYVFEGSRHPWITRPRYRVGIRGDSGDRISFLSQSL<br>NDRNLKEILLKQTPQRTTIRKEFPETWFFENVGEEEFTLTKKIPDTITSWVVTGFSLNPTSGIALTKNPSKIRVFQPFFVSTNLPY<br>SVKRGEVIAIPVVIFNYLDKTLDADVVMDNSDQEYEFTEATNEVLEKAIDEVRRVKRVTIPANSGKSVSFMIRPKNVGFTTLKI<br>TATSALAGDAIHQKLKVEPEGVTLFENRAVFINLKDQPEMSQSLDADIPNEVVPQSEFIEFSVVGDLLGPTLQNLDNLVRMPY<br>GCGEQNMVNFVPNILVLKYLEVTGRKLPSVESKARKFLEIGYQRELTYKHDDGSYSAFGKSDASGSTWLTAYVMRSFHQAG<br>TYTDIDPKVITAGLDFLVSKQKESGEFPEVGKLFDNANQNPLALTSFVLLAFFENHELIPKYQSAIKKAVRYVAEEADKTDDQ<br>YSLAIAAVALQLAKHPQSEKVIAKLESVARKENDRMWWSKATESTGEDGRVFHWKPRSNDVEITSYVLLALLEKDPAEKAL<br>PIIKWLISQRNSNGGFSSTQDTVIGLQALTKFAYKTGSGSGTMDIEFSSAGESKNTIKVNPENSLVLQTHDLPKSTRKVDFTAK<br>GTGSAMVQLSYRYNLAEKEKKPSFKVTPTVKDTPNQLLIVDVCAEYVPLEDADKDKDSNMAVMEIALPSGFVGDSTSLGKI<br>QAVDRVKRVETKNSDSTVVVYFDSLTPGDVRCLPLEASKAHAVAKQKPASVSLYDYYDTERKATEYYQVKSSLCDICEGAD<br>CGEGCKKD
**DmTEP3**	MRLQGADMGAIPVLILVTACLLCQTSAQGLYSIIAPNTLRPNSQFHVAVSLHNAPESATFKVGILGSSYTDFKTVELRPFSTQL<br>LHFEIPALRTDRYRLTAEGLGGVQFTNETQLHFESKQHTVLVQTDKSIYKPGDLVHYRVLILDANLKPARGYGRVHVDIKDS<br>GDNIIRSYKDIRLTNSIYSNELRLSDSPRFGTWSIVVDVSDQEHTQTFEILDHILPKFVVDIDTPKHAIYKDGKIAATVRAHYAF<br>GQPIVGEATLSIYPTFFGSLQPFVNDLITRKVVPIDGNAYFEFDIENELHLKQDYERQYLLDALVEEKSTGSVQNYSTVLTLHL<br>NHYRVEAVKVPSYYIPGVPFEATARIARNDGGQLRDFNPQITAYLTNVYGSSEMYNRTAYSLDASGEIKMKFTVPIGDRDEF<br>HSIIVDYQGVISEVGKIPSKHLHSKNYITAKVLNDRPTVNQEISVVVRSFAPIKYFMYQVVGRGDIILSRNVDVAPGTFHTIKFL<br>ASFAMMPRANLLVYTVIDGEFVYDEQVIQLEENLLNAVQVDAPIRAPPGQDIDIGISTKPYSYVGLMLVDQNANDLRSGHDL<br>THKRLMDALRSYELSDVNTPMGSPGKESGVITMSNTDYFIEKEAESNPALDREVSTGPEEDKLTTVRKTDIGPAHKIEVNTLP<br>PGKGRYAFSYTPKPFWHNPRVHVMRDPADTWLFLNISASSDGRNSIHRRIPSEMTSWVVSAFALDPVNGLGLSPPNGKLEAY<br>KEFYISTELPYSIKRDELIAIPFVVHNNRDSDLNVEVTFYNSALDFDFPQLDPKATNQPKVELYRRRSLQVPGRSARSVSFIVTP<br>KRVGPLLVKAMAASSQAGDTVEQNLLVEHPGAMERINRGFLFELNSNAQNRRNVTIAVPRNAIPESTRIEVSAVGDLIGSLVG<br>NLDSLILLPTGCGEQTMVNFVPNLIVLRYLGRLRQLTPEVELRATNNLAIGYQRILYYRHENGAFSAFGLDIKRSSTWLTAYV<br>ARSLRQAAPFTQVDSNVLQKALTYLGSVQSANGGFEERGDVFERFGDDGISLTAFVTLALMENVDLYPEYRNNINKALDFIT<br>RGLDGSSNLHAMAIGTYVLSRANHNAKAAFLQRLDSMATNKDGLKWWNKTAPAGEQQSPWYNATRSVNIEISAYAALALL<br>ENNLVGDALPVLNWLMDQRNPKGGFVASQDTVVGLQALLMFAERFSSQGNNLQIGFHYGEGAETIINVNAENSLALQTVEL<br>PNNLKNLSVSATGRGMALAQVSYTYNTNVTSAWPRFVLDPTVNRNSHADYLHLSACASFVSVVGENEQRSNMAVMEVHLP<br>SGFVVDRDTLPTLESSERIKKVETQNRNTKVVIYFDYLDRREVCPTLHAYKTVKVTKHRPVAVVMYDYYDSARRARQFYRA<br>PKSNICDICEHANCGDLCEKAEKRESKRPDDYTAIAGHSSGSRHTAIPLASVVMVLSMLLKTLSC
**DmMCR**	MMWHLLRALLVVAAVLDALQPAVGQNDNYYNPNQNQQNPQQPLLPNQQWGNNPQTNQYSNNNQNFGQTNPSDRPPYRT<br>DSGSYNDIAGQDDYNKRVGGGYQDNEEPSLTRGKSSYNIKATFLESLHSREPTYFIVASRMVRPGLIYQVSVSILQAQYPITVH<br>ASIACDGVQISGDSKDVKEGIPETLLMRIPPTSVTGSYKLRVEGFYQNVFGGLAFLNETRLDFSQRSMTIFVQTDKPLYMQGE<br>TVRFRTIPITTELKGFDNPVDVYMLDPNRHILKRWLSRQSNLGSVSLEYKLSDQPTFGEWTIRVIAQGQQEESHFTVEEYYQT<br>RFEVNVTMPAYFFTTDPFIYGRVMANFTSGLPVRGNLTIKATIRPIGYFSNQVLNEKYRLGRSPLEQTNLYNERWRYNNPNQN<br>PQVQYNVPGQLPQDGADLSQDILYRNQYVVERHYQFDEEWPFWVRKPEYQDSSYEAWSGTYRKTLPYLRYFNGTFDFKWP<br>LRELELLVPNLANSEVLITATVGEKFYDEIISGYSVARVYNSSLRVVFLGDSPQVFKPAMPFTTYLAVEYHDGSPIDPNLLRQG<br>LMEVSGFVESRNGGRRDWPAQRLPMSQQSDGIWEVKIDIRNDLNLDDRPQARDFLNGVQNMRLQANFVDPRGERIQTELLL<br>VSHYSPRNQHIKVTTSTEKPVVGEYIIFHIRTNFYLEEFNYLIMSKGVILVNDRETITEGIKTIAVVLSSEMAPVATIVVWKINQQ<br>GQVVADSLTFPVNGISRNNFTVYINNRKARTGEKVEVAIFGEPGSYVGLSGIDSAFYTMQAGNELTYAKIITKMSNFDEQTNG<br>TYKHIWYSHEGNPDELVYFPASSFGVDANRTFEYSGLIVFTDGYVPRRQDTCNRTLGFGECLSGRCYRLEKQCDGLFDCDDG<br>TDEINCHARNDTELLNYRKYRFNRVLRHYENVWLWKDVNIGPHGRYIFNVEVPDRPAYWMVSAFSVSPSKGFGMMNKALE<br>YVGVQPFFINVEMPEACRQGEQVGIRVTVFNYMITPIEAIVVLHDSPDYKFVHVEEDGIVRSYNPRTSFGEHQFFIYLEAQGTT<br>VVYVPVVPQRLGNVDVTLHVATLLGTDTITRTLHVESDGLPQYRHQSVLLDLSNRAYVLEYMHVNVTQTPEIPYQVDRYFV<br>YGSNKARISVVGDVVGPIFPTMPVNASSLLSLPMESGEQNAFSFAANLYTIMYMRLINQRNKTLEKNAFYHMNIGYQRQLSF<br>MRPDGSFSLFRSDWNNSDSSVWLTSYCLRVFQEASFYEWENFIWIDATIIEKNMRWLLQHQTPQGSFFEVTWLPDRKMNRT<br>NFDKNITLTSHVLITLATVKDISGTLGSRVALATQRALAYIERNMDFLRHQAQPFDVAITAYALQLCNSPIAEEVFAILRRQAR<br>TIGDFMYWGNQEIPQPPRKLENQKWFSLPRLPYEYDSLNIETTAYALLVYVARREFFVDPIVRWLNSQRLNDGGWASTQDTS<br>AALKALVEYTVRSRLREVSSLTVEIEASSQGGKTQTLYIDDTNLAKLQSIEIPDAWGTIKVQAKGAGYAILQMHVQYNVDIEK<br>FQTKPPVPAFGLHTKAIFHGRNQSHISYVACQNWINQNESERSGMAVLDVAIPTGYWIQQQKLDTYVLSNRVRNLRRARYLE<br>RKIVFYFDYLDHEDICVNFTIERWYPVANMSRYLPVRIYDYYAPERFNESIFDALPTYLLNICEVCGSSQCPYCSIYNMGWRAS<br>MSMSLLFFSVFIYLLRSRTHLVLNMMQLLT
**AaTEP21**	MSVFIQTDKPVYTPGDLIRFRVIVVDADTRPVTSIKTVNIAIDDSAKNSIRKWPYAKLLNGIFESQVQLASSPVLGTWIINVTAS<br>DDIIVTKQIEVKEYVLPKFFVKVYPSEVLLGKNKKVSLTLDAYYTFKEPVDGNYKVELFLDHTKRKPDFIKSDRITGKTSLEFQ<br>LKNEVDIDGDEQYTDVTVEVEVVEAFSNRTVSITENIPIYRQPYTVTLLPSAPSFRPGVPFNVQIVVKDQLGHPPAEEKAASIDL<br>TVEFHLPIDSDTKSITVDLDEKGTGQLTLEPRPDAQELKVNATYDSQQYDVIHDPIHGFSSQSKQYITVTLNPKYYNNIKVDKD<br>IVLDISCTETMTHFSYIVVTRGNIVEASNVPVRIKKKHSLRLKMTSKMSPESRLLVYYTNREYLIFDDIELKFDSFNNDFKFDLN<br>DDEYFPGQSVYIDVYASKDSYVAFSGIDESVLLVGKERHDFNKGDVLKELALYGATNDAEFDLFHVSFMSNGLIIPVNVSVT<br>RSQNARFGTLLGRTRQQAIEIRTQFLESWLWKSFSMDGRNNFKAIEDSVPDTITTYHVSGFALSPTLGLGVIQQPVSFTVRKKF<br>YLVANLPYSIKRGEVALIQVTVFNFLGSSITTDVTLFNKRDEIEFVENASTNNTHRTKAVIVPNNNGKSVSFMVKAKKLGQIAI<br>KFQAVNLLETDALEHMLRVTPESHRYEKNVARFVELPKFETQTFDVKLDIPKNIDEGSAQIKFTLDPDILGTAISNLDGLIRKPF<br>GCGEQNMLHFVPNIVVLDYLNETNTAAEDVRTKAINFLSSGYQNQLRYKRSDGAFSVWGQSYAGSTFLTAFVAKSFKIAAK<br>YIQVDKSIVDAAFDWLVKQQQSDGRFPEVGQVFQADMQGGLRNNGFALTAYVLIAFAENKEVYRKYQSQLNKTTNFIADRL

	ANMENPYDLSLSTYALMLTNHGKRTEFLHKLVEKSIFDRNQTERYWDSKPVDIEVAGYALLSYVAAGKLLDATPIMRWLNK<br>QRYGLGGYPGTQETFVGLKALATFAANVTSRRNEYTVRIFYEPNGRRTFDVHMHNSFNIQELDIPNNIRKMKVEVEGIGRGFF<br>QVAYQYYQNMQVAKPSFSITINQLNTTTEHMQQLDVCVKYIPKEAYQKSNMALVEIFLPSGLVADSDAITDKTGGIRRIERRF<br>SDTSVVIYYDNLDPEDKCFRVTAYRRYKIALHLPSYIIVYDYYNFERFAIQKYEGKVLQLCDICEDEDCETLSCQNSSKLAIM
**AaTEP15**	MIFYSTGKKSVADYEYVAVEESIVPEHQLQEAESVPDTETNEADGSSPVKEGKIRREFPETWIWNNISDECFSGEKTITKKVPD<br>TITSWMITGFSVSPVYGLGLTRQPRKLNVFLPFFVSTNLPYSVKRGEVVSIPIVVFNYMDSDQTAEVTFHNTEQEFEFADVENE<br>VHENPKLELFRKKTVQVASNTGKTIPFMIKPSKLGHITIKVTATTQLAGDGVERQLLVEPEGLPQYVNKASFVDLRATPEVNN<br>NFTVEIPKNAVPDSTRVEIAVIGDVLGSTIQNLDSLIRMPYGCGEQNMLNFVPNIVVLDYLKNTNQLTASIEDKAKKYMESGY<br>QRELTYMHDDGSFSAFGKSDPKGSTWLTAFVARSFKQAADHISVDEKIIDKSLEWLSDQQSSNGSFPEVGKVSHTDMQGGSG<br>QGIALTAYTLIAFLENKNLIPKYTNVINKALDYIVRNTEGLDDNYALAIAAYALQLADHSAKDFTLSQLDTKATTDEDQKWW<br>NKPIPEADKKNPWYSKPNSVNVEMSAYGMLAFMEAGLDSDALPIMKWLISQRNDKGGFQSTQDTVVGLQALAKLAAKISS<br>KNNDVTIVVTYNENQQKEMKINSENNMILQKFELPSSAKDIDIKATGRGFAVVSLGYKYNMNVTGEWPRFVLDPQVNKNSN<br>QDYLHLTVCTNFVPTAGQNKSNMAVMEVGFPSGFTADSDTLPSLENTEYIKKVELKDGDTVVVMYFDSLDRNELCPTVSAF<br>RTHKVAKQKPAPVVIYDYYDNSRIARQFYNGPAAGLCDICENEDCGNSCSIKSQKQRSPKEEATVMAKSGAVGITIGLQLIVA<br>ALLVRLFN
**AsTEP15**	MIVTNISPTKKEFECEIVDSNDVIVGWYPMAVPPYGLKKALMRVEGLEGKGYKLQVWDKHKRTLLNSTALECIRKSYLVLF<br>QTDKPAYKPGDRVQFRVIILYPNAAPVIRIQPDFFITDPDRLRMKQWLNATLTSGVFEGSFQLAEQTTVGLWTISTSLFDQQYK<br>DSFLVEEYTLPLFKIETQSVPKRLLHCKDPKMSLKLRASYVQGGAVRGNATVIVRTHFNNYPSQTKEVARKNLPINGVALVN<br>FPTDLVAKNCDEERTVWFDVKVSESSTGVSYNATSTMTVHNSEGVTMEVLDGKDAFYPGQPMRVKVKVATILDERPLVRR<br>NVRILYRVVDEDHDERDEAMPNVLVLQTNGNGIVHFTVNTTIKTVEVNVEGMYNNQTIPLVFAYPMYEDKSFDYLEMHSRN<br>AYHTLDRNITIDLYSNVMLQRIYYVGYCMSRIAVHGVHEAKNPVKHHRLEIEPIRLMNPRMKLLAYAMKDDGKILSSAIIIRF<br>KPTSSTALNITATLKEPNSGKYEFNVTTEEEAFVGLLGVDERILQRSTIDNNISQRKLDKEMQDFEDPSGVWSPYDSFGSVGM<br>TILTDGYLPDVGFVPHLSELARAGTAPEQDYSTREDFPESWIWESAQAPNGKALFEKSLPDTITTWIVTGFSVSQRNGLQILKE<br>PLKINSKKWIFAQLHMPPSIKRFEEVTVHCLVHNYGAAANVTVEVRPKLKAVTPLFLAQGATETVRIKLKSASVGELAVEVIL<br>RGPTGKVIDALHQTIPVRPEGLIKTVEDVRVLSFPRHGKQNFSLSLPAIEKDRGTSSGEVTLSVIGTILNLNFFDLEHMVKSSHG<br>NGEENLLFLQTTMAVYDYLEKTNRLPPASKEKLLTYMEVAYQQMLSHRLEDGSYSTFKRIHKCGDVWFTASALVALQKLA<br>KYIPVEDSLLTDSLDWLVLNSAEDGGYNESCTIVHPHIQRTGGRELSLASSVLFAFVGGGSSKQYEQLTKKTVSLLLSSPIEDV<br>YLLAKTTYLLSLMGHPESVGMLVTLNTMAVTDGKYRFWRVARRESTSLVDMRDQEATAYALLANLKQNTIDQVEMVRVA<br>QWLQKHSFAGDRCTPSLERVIALEALATVAHLIPSIVPSMYIAVGNVRFEVNATNKELLQTVTLPKGTRSVSVSVRGSGLVLI<br>KLSYRYSLANATTTGKTASNRVPNKGPIGVNIKKKAVKNSMLALHMCFSLIQPLYVVYESDLWKAAIDLPTGYEIDEQQQRYD<br>NTTKSAILLENNSHLELVWDVRHVTEVCYAVTAISRLLPVDLMPGLIYLSSFDTKDIVAKFKQCFLRDVEYHLVVKCSELLPI<br>RKTIKMGKPSHYSIIGSRILRPNSVYCVQVSTFDTKSPVAFRISIVAKEKSIKSEEITLTGNESRLLRFTIDSIPEDEYELMAEGLS<br>GLVFKTKAHIDFDNKFCSVLIQTDKSVYKPGDTVRYRVLVLDRYMKPLPVDDGGMMVYIRDGKGNRVKQWNDASLGECG<br>VFQSELTLSTEPVLGAWDINVDVLGLKHTKSFDVDEYVLPTYEVTLESPGHTFLDDELLKLVINSKYTYGKPVVGELTVSIKV<br>GQSMCFGRGPNTSSICQKVVPTEGRTVVEFNLKEILLNKMYIRELQIEAEVCEALTGRTQQGSTTVTLHENRFEVRFIEESSYF<br>PGLPYNAWIQVTNLDGSPVREGTREVEIALRNYNTDLFKQTGTLDDAGKVKLNVQLDELTFDYVSVEVKFRGKDYYVQGIT<br>KPRADQEAFMKAMPLEKLTIPGTELKFDVACTKPLQLVAYSLLARGELLAGGAVQGADNKQTSITIPSSYCMVPRAKLLVHY<br>ISPTGYIVSTYTEVKFGRLFENQIQLTLSKDEVKPGESLDIDVRTEEGSFVGLLAVDQSVLLLKSGNDIGREQIEQELEMYESSQ<br>SHHRYWNNNSTSDCQSVGAVLLSNRFIPKDIFPRGVMFACRAAPMGGVFGAAPQMMLKRANMPMMEADMMMESAAEPTI<br>RANFPETWIWESIINSKETESIRKIVPDTITSWIITGFSLSKSQGFGLMDSPSKVNVFMPFFVTIDLPYSVKLGETVRIPVIVFNYM<br>DEDQTADVVFFNDNDEFEFFSDDAADQQDKHRQEKLPVSRGGGKTLTFMIKPTKVGHITLKLTAKCALAGDGIERQLLVEPE<br>GLPQYINKALLIDLRSVKEVSQTFEVQVPADAVPDSTKVEVSVIGDVLGSSIENLDSLIRMPFGCGEQNMLNFVPCIVVLDYLK<br>ACRRLTVEIESKAKKCMEVGYQRELTYKHQDGSFSAFGESDKSGSTWLTAFVAKSFQQAAAHITIEEDVIDKALQWLSKIQS<br>ADGAFPEVGSICHKDMQGGAGSGIALTAYTVVAFLENAKLGEKFKSTVDKALAYIQQHIAELDDVYAHALAAYALQIAKHS<br>LKDEVLAGLQSKATKEGDMQWWTKSLPEKTETDCWWHRPCSVNVEMSAYGLLATLETCTGLEGLPIMKWLVSQRNDKGG<br>FESTQDTVVGLQALSKMAAQLSSSEADVSIKVTAPNGQERNISVNKHNTLVLQKHELAVDTKKVDMAATGTGCALFQLSYK<br>YNIKDVDKAPRFMLKSEAKKGSIKSCIDLTVTTSFIPQEDQAVSNMAVMEVDMPSGFIVEADVLKQLKELELVKKVETKRGN<br>TTVVLYFDNVSEDVIKLRMSAFQKHEVENAKPANVIIYDYYDNSNHYSIVGAKLLRPNSEYHVAVTNQDVSEPIRFSLAITDA<br>SNVIEKQEITLNTGETRLVPFVIGDIPESSYKLVAEGLSGLTFKNETDLEYQQKSFSVFVQTDKSIYKPGDTVRFRVLVLDPNTK<br>PLPKADSINVHINDAKANRIKQWKEGKLVKGVFESELTLSTAPVLGAWTINVEVLGTKHNKVFEVDEYVLPKFEVTVESPGIT<br>TFKDGKVKAIIRSKYTYGKPVKGEATVSASPEFQFHYVQPFAKDVITRKVVPIDGKGSVEFDLREELRLEGDYTRNIVIEAVVE<br>EELTGRKQNASAKVMIYDRRYKMELIKSDDNFKPGLPYTAWLKVTYQDGAPVQDQTNPVEVKQSTYESTQFLRNYTLDQN<br>GMAKLEINTDVNSTYINVVGVYLGQEFYLSGISKADSDVDSYIRARVLTEMPLVGSDVVVEVSATAPMKFFTYQLLGRGDVL<br>LSNTVAVPESKTHKFKFPATFAMVPRAKLVVFYIAPNGDMVSDSKVITFDSELQNFMKVSLSKDQTKPGQDVEISISTNPDSY<br>VGLLGVDQSVLLLKSGNDITKEQVFSELEKYEERSYGYYRRKKRFAWNPHIEHQDFNTVGAFVLSNANDPPHDVIDIRVAEE<br>DRIGGPLLQYAPSTSSLGPAGDASSSPTPAVRKSFPESWIWHTIARFEQLQYSHAHVEMSAIKFSASQSQTGFRLCMRPGERAN<br>NTLPEVPIYKFHRAPNGTVLYTTIEKPRSMQNQRQVLKTNTRPPLAGPFAFSRIPRPHRDIPRIFLSQEIQNTWLFDNTYSGFSG<br>EKTLQKKVPDTITSWIITGFSVNPVYGLGLTQQPRKLKVFLPFFVSTNLPYSVKRGEVVAIPIVVFNYMEDDQTAEVVLHNDE<br>QEFEFADVENEVVESSKVELFRQKRLDIASNTGKSVSFMVKPKKLGHITIKVTAKTKIAGDAVERQLLVEPEGLPQFINKAAFI<br>DLRAVPEATKTFEVEIPKNAVPDSTRIEVAVIGDVMGSTIQNLDSLIRMPYGCGEQNMLNFVPNIVVLDYLKATNKLTANIEA<br>KAKKFMEAGYQRELSYKHQDGSFSAFGESDKSGSTWLTAFVARSFKQAANHITIDEKVIDKSLEWLSDHQAPNGSFPEVGVV<br>SHKDMQGGSGSGVALTAYTLIAFLENINLVDKYKNTINKAIDYVYRNTESLDDTYALALAAYALQLADHSSKQLILSKLDAK<br>ATTDSDSKWWHKPIPEVEQKNPWYSRPNSVNVEMSAYGLLAFLEAGLDTDALPIMKWLIGQRNDKGGFQSTQDTVVGLQA<br>LAKLAAKITSPNNDVAIVAKINENQEKRMSVNAENGMILQKFELPSAARNIEIKATGSGFAVVQLSYKYNMNVTGEWPRFVL<br>DPQVNANTNPDHLHLSVCASFVPSAGQNVSNMAVMEVGFPSGFTADSDTLPSLENMPFIKKVETKDGDTTVVLYFDSLDQR<br>ELCPTISAFRTHKVAKQKPAPVVIYDYYDNSRIARQFYEGPKASLCDICENEDCSEACSIKSQKQRSSDSPSREPTVDNGPTLSS<br>SSPTVRISFFTFLFATVLVTIFH
**LlA2M**	MENYHVLYQAVAALCLTCCGLSNASPTLIPPTPPALAQVGGEELGYLLTMPQTLQAGAREQYCVTLHGNAQDTTITLELLEV<br>EKGIADEFTHTYVNGESQCNTFTVPNEPGTYEVNLQNDTHTLASSQVQVLGNNLITFVQTDKPMYKPGQKVKFRILTMTRNL<br>RARVGEIETVFIKDPNSFKVKQYKNVESEGMASLEFQLISDAKLGTWAIEVTMDGEVTTQHFKVEEYVLPRFEVTVEPPSYFL<br>VTSETFDGTICAKYTYGKPVKGFLTVQVCPSGYGAFGQACAIRQTQIDGCHKFSINSSEILSTDRYFYGRSFSIDASVREADTG<br>VQVNGTALGPEVSYDPLKMEFLDDTNGYFKPGMPVYGKVKVSKPDGSPAEGEEVLVIARDHSNSIYLEKVFQVNEMGEIHY<br>SICQGVMENTSSLSMFAEAKNFKVNEEAPYGRRLYTPTAHGSFRQWFSPSLSYIQIPALRAPIQCGEELSLNVPFTHDGSRPYV<br>KFHYQVMSRGRIMHTGTTTAPTLPRPLDFMEPLMENCLEVGEEPTRPPLVTDIPEPPMPEVENMTDSSEMAPRPPVMVGEPE<br>AFDESSLSMVQPPERTAAVAEVYNEPERVPALELPLLEPLEPMRIEPISESLDLGDEQVRRKRSIMRPFGHREEEEIVRSESISDQ<br>LSSVQLDIPVTAEMSPSFSILLFYVRDDGETVADSMEFSVEPCFQNEVKMEFADKTVAPGEQTVIQLEAAAGSVCGVGVVDK<br>SINILGGDHQITPAQVFEKLKEYSLSSRSRYTYFRNDNNYCKEKMEKKAKLEAAASKDGEGGEEEEDAEEEFVRFPDFWYRS<br>NYVDAIEAFKQMGMLVLTNLAVETRPCAQDIPVYYAYVESGIPGPMAAVQVLDSAVIDERRLAPASTKADVRSHFPETWLW<br>DLFTLGESGARNISVTVPDTITQWVGNSLCINEKAGFGLSSVTSLTTFQPFFLSFNLPYNAVRGERLPITVTIYNYLDKCLHMLL<br>RVDGMKGFKVHGARANRDPFCLCGGLSKTTKFFITAQDIGKLPIFAQAEIVPGECGNSVVMDTQYVGMTDAVQREVLVKAE<br>GVTQRYSHSLYVCPTGNEMYRENFRLPLPSDMVPDSARGDVTVIGDIMGPALSNLDGLVRMPTGCGEQNMVGFTPNIYVLK<br>YLTATGRLTEVVQDKAKNFMEIGYQRELKYRHSDGSYSAYGERGHKPGSTWLTAFVVKSFAQARPYIFIDEDDLNLSMDYL<br>KSVQNPEEGPLMGCFFENGEVFSSYMQGGLGRDGNDAALSAYVLIALLEGGLSPEDAAAVDAIDCINRQLSLSPQVDVYSLS<br>LVAYYVNALYNPSSPVTTQVMDRLNNATTVSDDLKYWKRRGEQPKPVNSWYYYSAPSAEVEMTAYGLLATLKFYDDDAIG<br>KAQSIAFWLSSQQSAFGGFSSTQDTVLGLNALSEFAELAFSDAPTDLELMVGVTEGAEFQKIFMVTDDNSLELQRHTFDMTD<br>NAVMLLSATGTGCALLQANVRYNKMPNKLGIDDPKFQLNVDPRLYQHDRNQCGRRTIYVTFGQSNSEQFSTGMTMLTVRM

	VTGWSAIPESLRELRSRFHHLGIERLEEKVEEGVINFYLDQLDNRVRRFALNVEQDRDLLVSSPKPAEVQIYQYYEKDVTVIK<br>SYELRTTCGTKEELPYDEPVDDGADDNGFVVQRRQPLGGAPLRIGASLGQPQANSNNCPVCDLETAPPNYNEIVCNSTVVYK<br>VRAGRAKKYSMKIKANLRPIKKVRLNLFANVRMDSSCKCPLLQKPLKTVLILTQATNLQGKTLTLDARTYVFSVRKDRTLER<br>KARKAQRKCRMP
**AfA2M**	MREVVLLLLSVTSVLATQPGYLFTMPRQLWQGTKEKLCITRFEEGDRLEYNLDLVHTNSNSTTRYIFESGSSQCWNFDVPNA<br>SGEYSATLSGSYGNGEEFTHKTEVTIQESSQITLIQCDKPMYKPGQTVKFRIMTIDALMKPKTETISMVSIENPSGIRVRQWRD<br>VNMSKGIASLEMALSSEPTMGKWTISVEVDGKNSKQTFIVKEYVLPKFEVTVTPPKYLLPDTPFIEGTVCAKYTYGKPVRGM<br>MGIGICYLNEYYYYGLRGNTDVRPCHKSLVEIDGCYEFSVNSSDLAMSSNRYSLWGRLSVKANITEFGTGTTLFGESSGPEMT<br>QTPYTIEITDETKNYFKPAFPYRAKVTAEKPDGSPAAEELIEITARNWEYNFFQRKNFTTDDNGVITFAVTDIPDNVTSISIQAV<br>SPRYQRSYDYYMIMDAHRLYQPQGYLSAQRWYSPSHSYMHIKRVTSTASCGQMLDVNVLYTTEANTTYKFYYMVMSSRH<br>VVYHSHRQHHFLRHDAVETNPVLESMKLEDFTPPPRRPFYRPPRLIPVRIPAVLVNDTNSTSTTESVPTEEPEPEMEVTTPEDV<br>GPEPEPETEEDMEEEPRGHIASFTIHIPIVAEMAPNAKLLVYYIREDTEIVADSITFNIEQCFNNKVDMRFEPKTAYPGSEARIRV<br>KAEAGSYCTIGVVDKSINLLGGNHQLTPEKIYNMVETPYFYHGYWSDDQEYCEKNFPQPEPENTDDTPRYYYGYYAYDTNT<br>VDAIQAFRDLNMVVFTDLTLETRPCHRRRRIPVAIHNRVYAIDGVGGPVDDVNVAYDSEPEVAAKSDKKSSKTRSFFPETFL<br>WDLELIGDEGEVVLTRNLPHTITEWVGNTICANTEVGIGTSPLATITAFQPFFLSFTLPYSAVRGETVPVTVTIFNYLQECLVML<br>VRLEPSDGFELMSPDRVVQRCVCGGDSDSIKYYIKPTQLGEIGILAAAESIQDDGSCGNNAVSDETTGVSDAVKRMLLIEAEG<br>VEKEYTFSSYMCAEGNSQLATIDLLLPPTGVIDDSARGKVSVIGDIMGPALSNLQGLLRMPYGCGEQNMASWSPNIVVLQYL<br>TNTNQLTSKIETEALNYMRIGYQRQLNYRHDDGSYSAFGNSNADGSMWLTAFVVKCFGQSRPFIDIDNDDLVKSLNWFRRR<br>QLENGCFPKIGYTHSYYLKGGISKSSNEAAMTAFVLIAMLEAGVSKEDNAVQSAVRCLXVQDNNDTYTLSMMAYAYTLYD<br>VNWPQRSLVMAELEERAMFRDDGMLKYWTRNDEEEATPEPYSWQWYKAPSAEIEITSYVLLSTIIGEQENAVTSAQPIVMW<br>LTKQRNTLGGFSSTQDTVLGLQALCVYATLVYSGVIHLDITFRGIRNEEKSFSITQDNNLVLQSSPLEVVPDTLEAEVYGVGC<br>AMVQAHMKYNIDEPPAGPAFNLRVNAFRSREMGNDCKRRTLNICASFTGPGGSVTLVDVKMITGRVPVTSTLDELVNDQSL<br>EIQKYEVDGNLVHIYFDKFDSAGQCFYFDVEQDIKVTDPKKAFIKVFDYYETDLQVVIQYNLRTTCGTKEELPEISVDQYLRG<br>IFFTSEEEIVQVRVPAGIDGTPGEPVITCPNCINATMNRKSAEFKNLVCGSQKVYKAVAGRSGLYPMKIYADMRSRKKTRVNS<br>YSNYEMRDSCLCDSIPTSESKVLIFGNDGTFERDMKKLYLDGEVTVMPWTREVEKSIRRLVTRRRRGCN
**LpA2M**	MEEIKWQKMSTLLFLLLLFTHDVYSKSGFILTAPKSLTPGKSNILNLHLFDIKTNGFLRIGVKDQDDGNVVAETEVSFNKDNPS<br>SSIQLTIPSGVEVKRPKLYANGSYSSPSSNDFFFEKDINMHKDKLIVFVQTDKPLYKPGQTVKVRILPTTPDLKLVPKETIGSFQI<br>ENPDGIVLGYWPMLSFAEGIAQFELALPDEPTYGMWRIKGNIEDTEIYENFEVKEYVLPKFEVKITPPSYLLTNADSITWKICA<br>QYTYGQPVEGTFVAETNVVKYNWEKEGVPVIHKEGLIDGCLDVTVNSSALGFNEQRLSYRAVNMFAEVTEKGTGIKMNAT<br>DSIYRTSNPLNIMYLEPTSGKGYLKPGLPFYGKLKVEKPDGTPAPGEQIELCRFADRERWNRKRWLEEKIRACKEFTSDEAGII<br>KFTVPPQTPDITSFRFKAKALQYGKKDGDNKLNQPQHSFTVSSWYSPSGSHLQLEPITEEIECGKPLTVKFKYTTGEEKKQKF<br>YYQIMARNFIVDTGSFEHEFLLSEDKSGLTDETYLPIDVTALSLNPPNEPEWENNVIVPPHIGETSLTLIPSFEMNPSAKILVFYV<br>REDGETVADSTKITVKKCLRNKVGLKFGEEKVLPGASSTLQLTASPYSICGIGAVDKSVHILSSDNRITEEEVFNKLGGHDYY<br>WPKQATSDYKYCEDYKFKQTEGEHEGSFSSGFTSTNYLDSITAFDEAGLVVISDMELETRPCKPSGFEDGGRPCPQYDVAFA<br>APQAANRIGGGGEAGGFGGGIRKKTNKPVVEIRTYFPETWLWELQNIGATGELSLKRDIPHTITEWVGSAICISEETGLGVSEA<br>ATVKGFQPFFVSFTLPYSVIRGEKVPIIVTVFNYLSECLPIKLSLEQSDKFEMQNDTNSYTSCVCGGKSDTTRWMIKPRSLGQV<br>NLTVYGASLPNEAICGNQDYSTVTARDAATRQLLVEPEGFPKEDTWSTFACPKDQNGKFTATSDLLLPEDLVEDSARGYVSI<br>TGDLMGPAIKNLDHLVRLPTGCGEQNMVKFVPNIFVLDYLTATGSITDSIKEKALNNMRKGYARQQNYRHPDGSYSAFGNR<br>DKQGNLFLTAFVYRSFAQAERFILINKNKLNETENWILNRQRSNGCFRKIGKLFNSALKGGISSNDETPAPLTAYVLISLLEAG<br>YKNETVIDQGISCLEALSNPSTYSLALFAYATSLAGHPSAKDYLAKLEERAITEGGKTFWKSPSSGRYYWGNSIGVEIAGYAV<br>LTLLQHGGASNLAKVTPIIRWLAKQQNYRGGFYSTQDTVIALQAMSKFATIIYKDELDLEVGVESSGFEKKIMLTKDNSILMQ<br>TFRLQTVPSPVDFEATGSGCGLVQTSLRYNVNTPPPRKGFHLEVTVKRGLYRDCINAHIATCVKYDGKGGVSNMAVLEMKM<br>VSGWIPDEESIKNIVDREELNLRRYEVDGNQLNLYFSELTDQNLCFNFWLEQDIEVQETKPATIRLYDYYELEQEVVTSYSIDE<br>NCEKLPPLP
**IrA2M**	MFLASVLLISAAVAASYVEGGFIFTAPKILRSETEALFRLTLTDVKDDGKVSVRLLKYNNDSIVLAEQEYDIKNGESTFLPFKV<br>PKHIDSQAKIEVNGTFGSYVFGDKKEIDFQKSKTNVLVQTDKALYKPGQKVQFRVLPINNELKPVTDVQATIYVTSPGDVRIA<br>QWNNVTFEKGIVQRDFKLSEEPELGLWQIVVELPSQTVRQHFEVNEYVLPKFEVTIKPPSYVLADAKEITWKICAHYTFGQPV<br>DGTLKVNVTYERYSWEKDDYPRVSHEGPINGCFDMTVNTTALRFNENYEIYKRLLLIASVNETGTGITMNKTSYVSRSFNPL<br>ELTFLEGEHGKNYFKPAMPFYGSLLVKKPDGVPVGGERIQLCLLSQSEIIKPLWWRTDRRLSCKNYTSDENGLVKFTIPPMKT<br>TVVTISVEAVAVNYDTVKYDTYGVKINQPKSTLYLQAWYSSSNNFIQVEPSKVPVSCTSRHPIRVRYTAEADKEIQFHFQVMS<br>RGKILKDAVVPVTFKADQAVVTEVDETYLAEEERNETLPSNVAEEQAATGSLEYELTPDFNYAPQVKVLVFYVRPDGEVIAD<br>AEQFEVEKCLQNNVTMTFGSDTVQPATSAAIHLNGSPHSYCGVGVVDKSVHLLKQDNQLTKEKIYDILKRLDISRYTWPKQA<br>SYDYCRKQLAKKPQQYKRTIWNGPRTSNVEYVDSITAFDESGMVVMSDLTLETRPCRKAIYDRPPYALAAPAMRVSYSSTT<br>YTGGAPGVATLDSLPEAIAVNREIPAKSAVEVRNYFPETWLWDLKELDENGQLSFKEKIPHTITEWVGSTVCINSQDGVGVSD<br>PAKIKAFQPFFASFSLPYSVVRGELVPVKVSVFNYLEKCLPIGLALAESEDYTIQDSASTTLCVCGSKSLTHKFLVRPTTIGEVN<br>FTVSAAGSTSDTVCGDQKVEKVVARDAVTRPLIIEAEGFPKEETKSVFVCPKDVTEGDGKNEFDLVLPDDLVEGSARAYVSV<br>TGDIMGPAVQNLDSLVRVPTGCGEQNMIKFTPNVYVLDYLKATGKHEEDIEKKAVENLKTGYQRQMKYRHPDGSYSAFGT<br>SDSTGSMFLTSFVVKSFKQAEKYVPIDAANLKESIKWVTTKQKTNGCFQNVGRVLSSGLRGKVNETAPGALTAYVLVALLE<br>GGLAEPKVVESALNCIAAQKNPSPHNLAVSAYAAALAGHSSAKDYVEKLEAVATHKGGLTYWSNAARKGSSASADIETAA<br>YAVLTYVKLGDKENLGKAQPIVRWMATQRNSRGGFSSTQDTVLGLQALSAFATHVSKDPVDISVKVAGTDVDESYDLKEPT<br>KLVIQEKKVTNLPNKLAVETTGSGCALISTTLKYNVHTPPKSEGFELTVTPTQEPNKCNTADVKVCLRFDGEQPSNMAVVEL<br>KLVSGFTVDEDHVYSLYRKEGVSLKRHELEKNQLNLYFEEISSTEKCFDIRVNRDVEVEDAKPANVKVYDYYEQENSKSVSY<br>TLNTSCQ
**HaA2M**	MDNLTRFLVLCCTLCSLTIAVRGSKGNFILTAPRAIDAGSIVYFTLTVFDIPQGGTVTLRLTHLISNVLIAESRVSVHNNYNTW<br>VEMNVPPMSSDISATLHIIGSFSSDYHIEASQNIHIRHNTILTFIQTDKPLYKPGQTIRFRVLPMDNQLKPLDANTMGDIWIEDPS<br>GIRVAQWNHEQFTEGIKQFELPLSGEPPLGTWNIHAFINQVTTSQTFIVKKYVLPKFDVSIKPPAVIMADAQTIPIEVCAKYTYG<br>KYVEGSLKAKVTYKKLWTFMYRDQRTIPSVEHQAELPGCHTFQVNTDDLLMQTEEFGGKELEIFAEVTENVTGIVRNATTSF<br>EISHQKVFLEFLRDNDYYKPKMPYVGQLEAKNPDGTPALSEKIQICVTLEGKQCLIFTSDKNGLIGFAIKPSPVSRDIRVEATTI<br>NYEDVHYSSTFWTRKLRKPTATMILSPWYSPSWSYLQIQPTTEEFECDKAQKVTVQYTAERGSTIKFYHQVLSKGRIVQQGS<br>HQRTFYSKVEEIQYDFEEQIVKKGSSSGNSSETEIGEFILAFDTRATMSPISRLLIFYVRDDKEVVADSRKFRIKKCLQNKVSLH<br>FRHEQQYPNTEATMLLSASPSSLCGIHMVDKSIRLLEDDTTFNTDKLFKIMESYDTGKDPTEFPGICIEDSKEDKPRMPRNMIF<br>QDTFPRSGRPYVDARQAFEEAGMTVITDLKLKSYHCTYYELPIPLMLPDSRYEDTEPQFITRVEALPSNSEEIRSFFPETWLWE<br>LHSVDSTGETAIKRQLPHTITEWVGGAVCVHPKTGLGIWDISSVTTFQPFFIDFHLPYSVIRGESFPLVVTVFNYLSECLPIKLSL<br>EPSDDYTLLTELRFQKTCVCGGQSSSVSFPVRPATLGMVNFTVYGYSIEQDDEACGNEITARLSARDAITKEILVEAEGFPKED<br>VFNYFICPENTNGSFATEIPLLLPDDVIMDSARAYMTITGDVMGPSIKGLKKLVSLPFGCGEQNMVLFVPNIFVLDYLTSTEKL<br>TDDIKEECLHNMKTGYQRELQYKHSDGSYSAFGASDKEGSLWLTAFVLRSFGQARRFMNVDENDLSATRSWILKKQFENGC<br>FIPSGTVLNKEMKGGLSSSEQSLAPLTAYVLISLLESDMEKHDTLVVKNALKCLESEKQPNIYVLSLFAYASALAKENETYGR<br>YLDELDKRAITKDYMKYWEPSSNSKSVAVEIASYYMLARFEMEEAKALKSVLPVVRWITHQRNSYGGFISTQDTVVALQAL<br>AKYASYISKNPVDIALAVETDDMTQGFKLDESNKLVTQQLKIVDLPTTVDIDAYGDGCAVVQFSLRYNVEKVSNTGGLELN<br>VNARRRGSNECNLPSLGICMRYAVHKEKTNMAVLSVKLPSGYVADEWSLLLLENDKEVQLMRHEIEENVVNLYFEEITNDA<br>RCFEFHVKSEFEVENVMPSIIRLYDYYQPDRQVTKDYSIPSTCNSTFLPDLTRFPLFKSSEPLHSDFDEFQEFSDTLNGELPEIITP<br>PETTDYQTESRNVSGSDWNSEETPDHLNDSLYPLEVLETSNSIDQREGNISQISTFVDVDHDLDFPDGLEGNMPVSVLPPPDFV<br>QPDCPVCSDSFPSNFSAVYCNSAFALKVMKRENNMKTVKIIQDVSFYIDSPKAIKKFGELEYEEECTCTELAEDGKILFIVGSPL<br>SLWNSNGKKHRIHLTSSVHVLLVPPKQIYSFITEAKSSCANDP

**HsA2M**	MGKNKLLHPSLVLLLLVLLPTDASVSGKPQYMVLVPSLLHTETTEKGCVLLSYLNETVTVSASLESVRGNRSLFTDLEAEND<br>VLHCVAFAVPKSSSNEEVMFLTVQVKGPTQEFKKRTTVMVKNEDSLVFVQTDKSIYKPGQTVKFRVVSMDENFHPLNELIPL<br>VYIQDPKGNRIAQWQSFQLEGGLKQFSFPLSSEPFQGSYKVVVQKKSGGRTEHPFTVEEFVLPKFEVQVTVPKIITILEEEMNV<br>SVCGLYTYGKPVPGHVTVSICRKYSDASDCHGEDSQAFCEKFSGQLNSHGCFYQQVKTKVFQLKRKEYEMKLHTEAQIQEE<br>GTVVELTGRQSSEITRTITKLSFVKVDSHFRQGIPFFGQVRLVDGKGVPIPNKVIFIRGNEANYYSNATTDEHGLVQFSINTTNV<br>MGTSLTVRVNYKDRSPCYGYQWVSEEHEEAHHTAYLVFSPSKSFVHLEPMSHELPCGHTQTVQAHYILNGGTLLGLKKLSF<br>YYLIMAKGGIVRTGTHGLLVKQEDMKGHFSISIPVKSDIAPVARLLIYAVLPTGDVIGDSAKYDVENCLANKVDLSFSPSQSLP<br>ASHAHLRVTAAPQSVCALRAVDQSVLLMKPDAELSASSVYNLLPEKDLTGFPGPLNDQDNEDCINRHNVYINGITYTPVSST<br>NEKDMYSFLEDMGLKAFTNSKIRKPKMCPQLQQYEMHGPEGLRVGFYESDVMGRGHARLVHVEEPHTETVRKYFPETWIW<br>DLVVVNSAGVAEVGVTVPDTITEWKAGAFCLSEDAGLGISSTASLRAFQPFFVELTMPYSVIRGEAFTLKATVLNYLPKCIRV<br>SVQLEASPAFLAVPVEKEQAPHCICANGRQTVSWAVTPKSLGNVNFTVSAEALESQELCGTEVPSVPEHGRKDTVIKPLLVEP<br>EGLEKETTFNSLLCPSGGEVSEELSLKLPPNVVEESARASVSVLGDILGSAMQNTQNLLQMPYGCGEQNMVLFAPNIYVLDY<br>LNETQQLTPEIKSKAIGYLNTGYQRQLNYKHYDGSYSTFGERYGRNQGNTWLTAFVLKTFAQARAYIFIDEAHITQALIWLSQ<br>RQKDNGCFRSSGSLLNNAIKGGVEDEVTLSAYITIALLEIPLTVTHPVVRNALFCLESAWKTAQEGDHGSHVYTKALLAYAF<br>ALAGNQDKRKEVLKSLNEEAVKKDNSVHWERPQKPKAPVGHFYEPQAPSAEVEMTSYVLLAYLTAQPAPTSEDLTSATNIV<br>KWITKQQNAQGGFSSTQDTVVALHALSKYGAATFTRTGKAAQVTIQSSGTFSSKFQVDNNNRLLLQQVSLPELPGEYSMKV<br>TGEGCVYLQTSLKYNILPEKEEFPFALGVQTLPQTCDEPKAHTSFQISLSVSYTGSRSASNMAIVDVKMVSGFIPLKPTVKMLE<br>RSNHVSRTEVSSNHVLIYLDKVSNQTLSLFFTVLQDVPVRDLKPAIVKVYDYYETDEFAIAEYNAPCSKDLGNA
**MmA2M**	MRRNQLPTPAFLLLFLLLPRDATTATAKPQYVVLVPSEVYQESLKRPCVSLNHVNETVMLSLTLEYAMQQTKLLTDQAVDK<br>DSFYCSPFTISGSPLPYTFITVEIKGPTQRFIKKKSIQIIKAESPVFVQTDKPIYKPGQIVKFRVVSVDISFRPLNETFPVVYIETPKR<br>NRIFQWQNIHLAGGLHQLSFPLSVEPALGIYKVVVQKDSGKKIEHSFEVKEYVLPKFEVIIKMQKTMAFLEEELPITACGVYTY<br>GKPVPGLVTLRVCRKYSRYRSTCHNQNSMSICAEFSQQADDKGCFSQVVKTKVFQLSQKGHDMKIEVEAKIKEEGTGIELTG<br>IGSCEIANALSKLKFTKVNTNYRPGLPFSGQVLLVDEKGKPIPNKNITSVVSPLGYLSIFTTDEHGLANISIDTSNFTAPFLRVVV<br>TYKQNHVCYDNWWLDEFHTQADHSATLVFSPSQSYIQLELVFGTLACGQTQEIRIHYLLNEDIMKNEKTLTFYYLIKARGSIG<br>NLGSHVLSLEQGNMKGVFSLPIQVEPGMAPEAQLLIYAILPNEELVADAQNFEIEKCFANKVNLSFPSAQSLPASDTHLKVKA<br>APLSLCALTAVDQSVLLLKPEAKLSPQSIYNLLPGKTVQGAFFGVPVYKDHENCISGEDITHNGIVYTPKHSLGDNDAHSIFQS<br>VGINIFTNSKIHKPRFCQEFQHYPAMGGVAPQALAVAASGPGSSFRAMGVPMMGLDYSDEINQVVEVRETVRKYFPETWIW<br>DLVPLDVSGDGELAVKVPDTITEWKASAFCLSGTTGLGSSSTISLQAFQPFFLELTLPYSVVRGEAFTLKATVLNYMSHCIQIR<br>VDLEISPDFLAVPVGGHENSHCICGNERKTVSWAVTPKSLGEVNFTRTAEALESQELCGNKLTEVPALVHKDTVVKSVIVEPE<br>GIEKEQTYNTLLCPQDTELQDNSSLELPPNVVEGSARATHSVLGDILGSAMQNLQNLLQMPYGCGEQNMVLFVPNIYVLNYL<br>NETQQLTEAIKSKAINYLISGYQRQLNYQHSDGSYSTFGNHGGGNTPGNTWLTAFVLKAFAQAQSHIFIEKTHITNAFNWLSM<br>KQKENGCFQQSGYLLNNAMKGGVDDEVTLSAYITIALLEMPLPVTHSAVRNALFCLETAWASISQSQESHVYTKALLAYAF<br>ALAGNKAKRSELLESLNKDAVKEEDSLHWQRPGDVQKVKALSFYQPRAPSAEVEMTAYVLLAYLTSESSRPTRDLSSSDLST<br>ASKIVKWISKQQNSDGGLLLTQDTVVALQALSKYGSATFTRSQKEVLVTSRSSGTFSKTFHVNSGNRLLLQEVRLPDLPGNY<br>VTKGSGSGCVYLQTSLKYNILPVADGKAPFALQVNTLPLNFDKAEDHRTFQIRINVSYTGERPSSNMVIVDVKMVSGFIPMKP<br>SVKRLQDQPNIQRTEVNTNHVLIYIEKLTNQTLGFSFAVEQDIPVKNLKPAPIKVYDYYETDEFTVEEYSAPFSDGSEQGNA
**CcA2M-1**	MDLNVSCCWKGLLLFSLLLVCVNGQTSGPYFMVTFPAVIESGSEAKLCASLLKPNESLVMNIYLVHGDQSTLLLQEKAEEEF<br>HRCFNFKAPLVEAESVQTMKVELQGESFKMTEERKVIFRSYHPLTFIQTDKPIYIPGQTVNFRVVTMDTHFAPLDQQYGSVVL<br>EDSQGNRIGQWTNVSSTRWILQRSYELNPEARQGVYRLKTYIGERMISHDFEVKKYVLPKYEVTVKRPNEVSVDEEELSIEVC<br>GKYTYGQPVPGKSWVKVCRNILPYLQARDRNPLCLEETTEIKKTGCAIHTIDVSVFLNSTLKSSLQDSLRVEAMVTEEGTEIT<br>MTKSETISLTYEIGKVTLTDLPKTYEHGSVIEGKIKLSNFKDAPIQNKEVYLFEVVNWSSKLLLNLTTDSDGLASFSLNTSSLPE<br>KDINLMASVYTEFHHRGYKTPYFSTDRKTVQLLRPVTPYTPTLSELIIENIEQPLKCDAEFTVTIKYYFIGETVEDFKTDIVYMV<br>LSRGLIVHHGYEKVEVKSSNGAANGTVSFKLSVGADLAPAVQILTYCVLPSENVAAGSTQFDVEKCFSNKVSLQFSPAKAVP<br>GEKNTLQLSAQPGSLCGLSAVDQSVLILESGKRLDTDKIFNLLPVQSVSSYPYTVEDQQECLHVRPRRALSTDNAYEALKRVG<br>LKMATNLAVRVPQCLSYRGLTYHRYSDIVMYRQHAPVSVLRMSSLEDADFATSSRDSPAVTIRTVFPETWIWELAEVGDSGS<br>AQVPVTVPDTITSWETEAFCMSSKGLGLAPPAQLTVFQPFFLELSLPYSIIRGEIFELKATVFNYLSKCIMVKVTPAPSSDYTLK<br>ASSDDQYSSCLCANERKTFKWILTPSVLGVLNITVSAEAESSQTVCDNEIVSVPERGRIDTVTRNLLVQAEGTEKTETYSWLL<br>CPKVDSLSEEVDLNLPKDVIEGSARSSVSVIGDILGRALRNLHGLLQMPYGCGEQNMAVLSPNIYILQYLENTEQLTSAIRERA<br>TGFLKSGYQRQLNYKHSDGAYSTFGYGDGNTWLTAFVLRSFGKAQKYIFIDPQIIQSAKEWLISRRDSDGCFIQQGRLFNNRM<br>KGGVNDNVTMTAYITASLLELETPVTDPVVSKGLSCLRSVIKDVKNTYTTALLAYTFSLAKDTDTRQQLFKKLEDVAISGGS<br>LLHWSQSASADDSDSLAVEISSYVLLAVLTADSLTTADLGFANRIVSWLVKQQNAYGGFSSTQDTVVALQALSLYATKVFNS<br>DGSSTVTVQSVGDTHHFDVNQDNKLLYQEKQLQNVPAKYSIEVKGSTCVSVQVAQFYNIPTPTEAKTLSIDAKIEEDCKTLG<br>QNFILNFTVKYDGLQERTNMVIVDIKLLSGFTADTTMLGTSSGKYAPLVERVDAKDDHVIVYLKEIPNNIPMNYQIQMKQVL<br>PVKNLKPAVVKVYDYYQTSDQSETEYSFHCQ
**HsCPAMD8**	MSGALLWPLLPLLLLLLSARDGVRAAQPQAPGYLIAAPSVFRAGVEEVISVTIFNSPREVTVQAQLVAQGEPVVQSQGAILDK<br>GTIKLKVPTGLRGQALLKVWGRGWQAEEGPLFHNQTSVTVDGRGASVFIQTDKPVYRPQHRVLISIFTVSPNLRPVNEKLEA<br>YILDPRGSRMIEWRHLKPFCCGITNMSFPLSDQPVLGEWFIFVEMQGHAYNKSFEVQKYVLPKFELLIDPPRYIQDLDACETG<br>TVRARYTFGKPVAGALMINMTVNGVGYYSHEVGRPVLRTTKILGSRDFDICVRDMIPADVPEHFRGRVSIWAMVTSVDGSQ<br>QVAFDDSTPVQRQLVDIRYSKDTRKQFKPGLAYVGKVELSYPDGSPAEGVTVQIKAELTPKDNIYTSEVVSQRGLVGFEIPSIP<br>TSAQHVWLETKVMALNGKPVGAQYLPSYLSLGSWYSPSQCYLQLQPPSHPLQVGEEAYFSVKSTCPCNFTLYYEVAARGNI<br>VLSGQQPAHTTQQRSKRAAPALEKPIRLTHLSETEPPPAPEAEVDVCVTSLHLAVTPSMVPLGRLLVFYVRENGEGVADSLQF<br>AVETFFENQVSVTYSANETQPGEVVDLRIRAARGSCVCVAAVDKSVYLLRSGFRLTPAQVFQELEDYDVSDSFGVSREDGPF<br>WWAGLTAQRRRRSSVFPWPWGITKDSGFAFTETGLVVMTDRVSLNHRQDGGLYTDEAVPAFQPHTGSLVAVAPSRHPPRTE<br>KRKRTFFPETWIWHCLNISDPSGEGTLSVKVPDSITSWVGEAVALSTSQGLGIAEPSLLKTFKPFFVDFMLPALIIRGEQVKIPLS<br>VYNYMGTCAEVYMKLSVPKGIQFVGHPGKRHVTKKMCVAPGEAEPIWVVLSFSDLGLNNITAKALAYGDTNCCRDGRSSK<br>HPEENHADRRVPIGVDHVRRSVMVEAEGVPRAYTYSAFFCPSERVHISTPNKYEFQYVQRPLRLTRFDVAVRAHNDARVALS<br>SGPQDTAGMIEIVLGGHQNTRSWISTSKMGEPVASAHTAKILSWDEFRTFWISWRGGLIQVGHGPEPSNESVIVAWTLPRPPE<br>VQFIGFSTGWGSMGEFRIWRKMEVDESYSEAFTLGVPHGAIPGSERATASIIGDVMGPTLNHLNNLLRLPFGCGEQNMIHFAP<br>NVFVLKYLQKTQQLSPEVERETTDYLVQGYQRQLTYKRQDGSYSAFGERDASGSMWLTAFVLKSFAQARSFIFVDPRELAA<br>AKSWIIQQQQADGSFLAVGRVLNKDIQGGIHGTVPLTAYVVVALLETGTASEEERGSTDKARHFLESAAPLAMDPYSCALTT<br>YALTLLRSPAAPEALRKLRSLAIMRDGVTHWSLSNSWDVDKGTFLSFSDRVSQSVVSAEVEMTAYALLTYTLLGDVAAALP<br>VVKWLSQQRNALGGFSSTQDTCVALQALAEYAILSYAGGINLTVSLASTNLDYQETFELHRTNQKVLQTAAIPSLPTGLFVSA<br>KGDGCCLMQIDVTYNVPDPVAKPAFQLLVSLQEPEAQGRPPPMPASAAEGSRGDWPPADDDDPAADQHHQEYKVMLEVCT<br>RWLHAGSSNMAVLEVPLLSGFRADIESLEQLLLDKHMGMKRYEVAGRRVLFYFDEIPSRCLTCVRFRALRECVVGRTSALPV<br>SVYDYYEPAFEATRFYNVSTHSPLARELCAGPACNEVERAPARGPGWFPGESGPAVAPEEGAAIARCGCDHDCGAQGNPVC<br>GSDGVVYASACRLREAACRQAAPLEPAPPSCCALEQRLPASSSSTYGDDLASVAPGPLQQDVKLNGAGLEVEDSDPEPEGEA<br>EDRVTAGPRPPVSSGNLESSTQSASPFHRWGQTPAPQRHSGRVVGAHRPGLLSPVFVYSPAFQSGGEEGLWMSNTCTLR

**HsPZP**	MRKDRLLHLCLVLLLILLSASDSNSTEPQYMVLVPSLLHTEAPKKGCVLLSHLNETVTVSASLESGRENRSLFTDLVAEKDLF<br>HCVSFTLPRISASSEVAFLSIQIKGPTQDFRKRNTVLVLNTQSLVFVQTDKPMYKPGQTVRFRVVSVDENFRPRNELIPLIYLEN<br>PRRNRIAQWQSLKLEAGINQLSFPLSSEPIQGSYRVVVQTESGGRIQHPFTVEEFVLPKFEVKVQVPKIISIMDEKVNITVCGEY<br>TYGKPVPGLATVSLCRKLSRVLNCDKQEVCEEFSQQLNSNGCITQQVHTKMLQITNTGFEMKLRVEARIREEGTDLEVTANR<br>ISEITNIVSKLKFVKVDSHFRQGIPFFAQVLLVDGKGVPIPNKLFFISVNDANYYSNATTNEQGLAQFSINTTSISVNKLFVRVFT<br>VHPNLCFHYSWVAEDHQGAQHTANRVFSLSGSYIHLEPVAGTLPCGHTETITAHYTLNRQAMGELSELSFHYLIMAKGVIVR<br>SGTHTLPVESGDMKGSFALSFPVESDVAPIARMFIFAILPDGEVVGDSEKFEIENCLANKVDLSFSPAQSPPASHAHLQVAAAP<br>QSLCALRAVDQSVLLMKPEAELSVSSVYNLLTVKDLTNFPDNVDQQEEEQGHCPRPFFIHNGAIYVPLSSNEADIYSFLKGMG<br>LKVFTNSKIRKPKSCSVIPSVSAGAVGQGYYGAGLGVVERPYVPQLGTYNVIPLNNEQSSGPVPETVRSYFPETWIWELVAVN<br>SSGVAEVGVTVPDTITEWKAGAFCLSEDAGLGISSTASLRAFQPFFVELTMPYSVIRGEVFTLKATVLNYLPKCIRVSVQLKAS<br>PAFLASQNTKGEESYCICGSERQTLSWTVTPKTLGNVNFSVSAEAMQSLELCGNEVVEVPEIKRKDTVIKTLLVEAEGIEQEK<br>TFSSMTCASGANVSEQLSLKLPSNVVKESARASFSVLGDILGSAMQNIQNLLQMPYGCGEQNMVLFAPNIYVLNYLNETQQL<br>TQEIKAKAVGYLITGYQRQLNYKHQDGSYSTFGERYGRNQGNTWLTAFVLKTFAQARSYIFIDEAHITQSLTWLSQMQKDN<br>GCFRSSGSLLNNAIKGGVEDEATLSAYVTIALLEIPLPVTNPIVRNALFCLESAWNVAKEGTHGSHVYTKALLAYAFSLLGKQ<br>NQNREILNSLDKEAVKEDNLVHWERPQRPKAPVGHLYQPQAPSAEVEMTSYVLLAYLTAQPAPTSGDLTSATNIVKWIMKQ<br>QNAQGGFSSTQDTVVALHALSRYGAATFTRTEKTAQVTVQDSQTFSTNFQVDNNNLLLLQQISLPELPGEYVITVTGERCVY<br>LQTSMKYNILPEKEDSPFALKVQTVPQTCDGHKAHTSFQISLTISYTGNRPASNMVIVDVKMVSGFIPLKPTVKMLERSSSVSR<br>TEVSNNHVLIYVEQVTNQTLSFSFMVLQDIPVGDLKPAIVKVYDYYETDESVVAEYIAPCSTDTEHGNV
**LlC3-1**	MFSHVGLGLVLVSSAVWQCMCEEPVADNSPLYFVATPNVLRLETEETVTVTTFGATSAATFSLYLEDYPERRRQLSHRTLVV<br>EKDEIANVSIKLTTGDLISLASVSSDPAYVYLVCETVNLTQASKTETILPLSLTPGYIYIQTDKPVYTPHQTVYMRVIALNEDYK<br>PANWPLEIDVLNPKDLAISRKQDDGNDVIFTDSLRIPDNPVYGNWTVKVKFANGLTTSSAVRFEVREYVLPTFGVTFQTDAD<br>QKVLLPSDTHLHTTVTARYVFGKPVRGHVTVTFGLIWHGHVFTLGKKRNLELNETGQAVCTIPISELRLPVESVWFPNGGKL<br>HMEAIVTEYASGKVERARDSSVVFAESLHIIKFTRSSRHFRPGLTYSLTIDVIKANGEPGAKLPLLVECNAQLADVTVQLPPLT<br>SSDDDDVLRTDDKGHLVVHYLIASNVKRLTFKVQTINEGGEPEKETDVHFGAVPFYSPSGTFLQVDATATSAHRRGSALEVG<br>DYVTVTTQYTSSEELSHVNLLVLSRGHIVWQTRTHNLGSNSTTFYFPLTRHMLPGARIVAFSIRGSQLGAEVISDSVWVDMKP<br>TCFGEIGLTLTEDNPPKFRPGDIGSVSVKGLPDMRVGLLAVDEAVYLMNKRSLTRRSVFDSLKEHDLGCGDGRGDSAAAVFR<br>GSGLTILSNTGLATLPRSTDNCRAKAVRRRRATSMDDVCCKEGARMNIQDPQLCHKAAKEVKKLTGLSACAMQFFRCCRD<br>MNRTELFTNSSGRIQGGQDQEEGLFDVTLEDDVMMPLSDETVPIRSNFPESWWFEDFTLGPTGEMELDFALPDSITTWVLQA<br>VGVSPREGLCVAPPLNVTAFRPFFVHVDLPYTAVRLEQLEVRATVYNYMQRSLNVRLFLQSVEGVCYSGVSGKNSEAFVVE<br>VAPNDATSVYFPIVPLEMGKFPVRVFAISAWGRDAVEKTLRVEGEGLEKVHTVSVLLDPSGKRFVRDRRGNQTFSLTNTVVA<br>EEQKQEVQLDLDLPSEVIPDTESCTVSAMGDLLGPTVRGIVDGAEEMLRLPTGCGEQNLIYMGPNVYTMRYLKAVGRLTSTI<br>EKKARAYIRQGLMRQMTFRKDDGSYGTWPHADSSTWLTAFAMKVLCQTNNFVPVDMNATCVSLHWLRTRQRTDGSFME<br>DVWVTHREMLGGVNGDMSVTAFVLIAMLECPCMEQDRGDMIARAMQYLEHRLEVKQRPLTAAITAYALALAGSEWSREF<br>NMKLMDQVLSTPQGLKYWSYAGDEDFGEEDKPYWYVKKPGALAVETTAYALLTQLTMNDVTASNPIVAWLLQQREEHGS<br>FVSTQDTVIALQALSEYSIKSFSAILDMTCHVTSEVDDKFQKTFSLTREDALVLKSVPEVPTGGKLLFEASGTGVGVMHVDVR<br>FNVPHDNNVCRFDLTVTSRRLSSMLQHFFWQPQHQQRCEPCSMDCDEREEEEEEEDIENFTFPPIIPRIREKLGLDKDRKRQPP<br>TTHTAGTRVGRPMSRIGRIPRGPRARARRSALIASSARVLCIEVCTRYRGDRETGMSVIDVGLFTGYKPITSDLHKLKEKGKV<br>DHYEMSQRSVILYIDEISHEKRHCVKFRTKQVHVVENLQPAKVQVYDYYNPEERCTVFYKPDNASGHLANFCDEQKRICQC<br>LEGRCGTCEETYAQKTWQELYRTACHNTSNALVVKILDRDLEKVGFERLLGVVEQAMHQPGKLQVSVGDKVVLLKRDSCI<br>CPRVQVGKSYLLLLQLPKKFKDVEGNQVFVFLLDKRAVVSEYQDPKDRSLSKPLRRQARKLARVARRLTRKRCRTKGKGK<br>RRVKVNRKNKIARALRRRRLRN
**LlC3-2**	MAAKCVFPIISLLLFMGVVSPLDGMPTFIVTSPKRAKFDTPTTFAISASNLAQPETIHLLLRTGDASEINLNETTLRFESDGVQT<br>WSVVMDSARMTDLLQRTFQLRAEYGSVVKSMEIPMNPLSGHVMIQTDKLIYTPRQTVMYRIIALDDDQRFAKWPVTVDIKN<br>PDLIILERQHHTSEQAFVGKNFTLPRDATTGTWTISATFEGTKVRTTKTIKFDVQEYVLPTFKVEVSVQEKVIALSTSWLRIKV<br>KAIYTYGEPVQGSVQMRLGFVKDDRIYLFNTSYSSDRGLDDNGEWSMAVPVRDMRSHRLWFPNASPLYIEANVTEAGTGEV<br>GTAVDMKTLFANPFYRISFDRSKKYFKPGFDYTMAIEVEAANGDKAANISVFVSVEMKTENGAFLQEGFGSTFELDAHGRM<br>NHTFPIPGAADGVNIRVVTLDPRAPELSEDTMTVNKFVSPTNTFLQLSQEGNSIRIQYTPAPQIFIGSRERITVVVISRGQLLHTA<br>SVPRDSSGSASLPLSRDLLVASSPAARVVAYFYIGGTNMEIVADSLFVDTEDICREELSLFHGFHGQQMAQLPKQTTELNVEG<br>GGNMRVGLLAVDERVYILRNERSLTRDKIFEMLGEADEGRGIGDGRDTDKIFEYAGLEFIKLTEAYEATVPVFDAMLHLSGL<br>NTYFPQGRRAKEFLSFGNPNIFDDEETIPLDEVRKYFPESWLFKEITLPEDGYGFESITLPDTITTWSLTAVGVGAQGGVCMSR<br>PLHITVFKPFFAQVRLPYKAVRLEQVTVDIGIYNYKTFDINVTVTVQADQGLCFSEGFGGQHRIQYRATLRARQTNSESIRMIP<br>LVAGSVKLIVDVRSTNGRERDIVEKTLHVVAEGRRVQKSISFPLDPSGKHSSHTPANRNGVRLTNTASISNRIVSKSNKQLTQI<br>NLYMPEEVIQGSEKGQISACGDLMGDIISTAVLQGDNLFESSVVDAEEAIGNLAPTVHALLYLNQSNFMTPYVQEKGQAHVT<br>YGVSRLLSYRKTVRDNASFGITPDSPPATWLTALVLKTLCHAKRITFIDTESLITRGFIWMSEQGHRETSGALNERDRRLARPS<br>RDYDLMLSAETLIALHECNSVEPINYEVLPLMGDLEMFLADNLGDVTSPLVMAKTAYALTLSDPRDDITLQAVQRLKDMMR<br>ETREGLHYWSVTSENELPQRPFWYHSGTRASSIEATAYGLLVFSRQAAAPEAGEGLYGFDYGDDEEEEEEEEEVVGAGGVE<br>WNVNLDSIAGWLIQKRNSRGAFIGAMDTAAATQALSEYSLSKQDLNSVDLHCNVTTRPTQNYTHSFDFNEENAITPKSLSNV<br>PVGRQLHIETRGTGLGQMQVRVEYNVPVQANAMCRYDVTITRRTMSYNSRSDDRDEVCRYCNMGCDDENHEVTEPPRTRR<br>SAAQLREARYRYRPQYGADLTARRRVKRQRITASRVSMCIEVCLRSTENNYADRTILSIAMLSGFEPYADDLTRIYEKRDQYS<br>LSSLVWVEEKETVELKFNQVPHDRNTCVSFRAIERRDVTRRNPAIVTVRPHDEQEPTCVHTYDPVVSEVNLAVYCANNQTSN<br>QGQCRCISGMCGACHTRPSDVDLNDMQRLVCDSEFIYKIRLTSTQVSDDWVDITADVIRTEKNGSDEVETEINMVTPRTCSCP<br>HTLGVGSQHYFFGSHYDLFVDRNNDKKKTYLLDQTTAFVSQSLGSSDQNRAGNWIRMAMGGHCGD
**MyC3**	MNSGLFYMCKQTGNTTMELLLVYLSLVLTLFGIGTAGPVYFVSTPSVLRFNSIETVAINIFGISDVTVQIYLHDYPNRTRTFSQT<br>SVVTTQGQTAKALVHVRPSDLPDEPDSQGYVYLVARSDDPRLIFENETLILMERNTGFVFVQTDKPVYTPHQEVKIRVITLDE<br>ERKPSASPVTIDVKNPQGVVLQRWTDSYQGRFILKKFQLSSIPYFGKNWTVTARYQDGVDTSHSVDFEVREYILPRFDVDIDV<br>QDLEIVLPSTEWINVTVSSKYVYGKPVIGGVTLTFGILASGQHETCSKTFNKLLVDGRTDFAVSVDELRDSLESYWFPDGARL<br>LIEAGVTDLSSGRKETAYHRSTAFSYFRYIISVEKSQKYFKPGLLYRLHVETYWVNGKRNNAPNVDIAVQITRYNDAGVLHSL<br>NRTEQTDEDGTFQYLVIMGHTETRMVIKVTALDTEVDEDEQDESVLEVSPLYSPSDSYLSIWTNLHETELMVDTISSGDNNKS<br>LSYLLMSGGRIIDAEQSHEIFHRPVTGKMSPGIRVLVFYTVNQTEGAEVVADSTWIDIKDMCEKELGVSKEKGRLEPGDGDGL<br>TLQVSGAPSSLIGFLAVDRAVYLLNNKHVLRRHTMFKRFASHDIGCGYGGGLTPAQVFEDSGLTVITNADLDTTPRENTGCN<br>RKRRGKRSIDDYETLHTHPCCVAGDMYGREGVEELDCYTEGKVVYHATNNTSCAKIFFRCCNIYRPADMTDIFDYGRVIRRG<br>TVKYTFRDVVVDLENTQVRSYFPESWMFEEDDYLDENGHLEKNFAVPDSITTHIIQAITLSDDYGMCISKPERLQVFRTFFVD<br>LEFPYSIVRLEQAEVRATIYNYSPDMLPCFVYIVTPEEICTPRSKSNYIKVEVHPNSPEPIKFPIVPLKAGEFDFEVRVYSINGTN<br>DVVRKTVYVINEGREEKKTVSFWLDPQAHRDKVGSTGDLTVNIMYPSPYLEIQEATVDLTLPEKAIPGTGMCKVSAMGNIM<br>DATVPVVLEGANALLDNVPHGCGEQTMILLAPVVYTMRYLQRTGQLRTGLEDRGKSRLRLGYQREMTFRKNDGSFAVWA<br>HRPSSTWLTAFVVKVLSEASEFIDIDSEVICSGMRWLFRHQRGDGAFMEIFPLFHKEMMGGMNGEISMTAYVLITLMEAHCR<br>PIDTEQNIVLALQYLESHLDFIDQTYPLALTAYALALGNSPRKMDAINYLKTASTEVDQGNGGQGMRYWSVIDFMGEVPPW<br>MTHDPTAMDVEITAYSVLAFLTVNEVGYVNNVVEWLLTQKTSSGAFKSTQDTVVGLHALSEYNVRSYALKIDLHTKIKVST<br>DDAIEHDIVLSDDDVSVQKAVHDIPVEGKLKVTTEGTGIGRMEVEVRYNVNTTEDERCKFTIDVYDSEVEFNYFQSKGLATD<br>MNCDACGHCEQEYEEDFEDVLDKYNKIELDPRIGVDQPRQDSRKKRAPYVMRMHGASKEQTCLEICVSYTGYEVLDMPVV<br>DIGLPTGFNVEKGDLEMLREKGIVNSYELSKRSVIFYLDQIPATDETCFKFRVVREFEVENLQAAKIEVYDYYKTEERCTKFYS<br>LKYDVANLDIFCSTSKRENCHCVEGKCAESWDTKIRYGRVSKVMSAFYRKICFEFDFALRIEVSNIVQKGNHLVIAAVIKSVT<br>KAGAEDLAVNDEIEFWMNIRCNLLLEIDHNYIIYGMNGIPYIDQHGVNRYRYFLQGNTVILKDYTLRRYSTSTKLEQWRHSFR<br>RMENYIHRWGC

**MgC3**	MRLSVILFFFLDFLMVYSEPIYFITAPSVFRLDAPETVSVSISDIDNISIQLYIQDHPAKEKTLYERTVTISSGEVKNTTFILQASDL<br>QDSDGNTIQYVSLVAKSTNGRFIFEKEKVILLSRQSGYIFIQTDKPVYTPNQEVRIRVIPLDEVRRPSTVPVHIDIKNPQGVIVQR<br>KNATYHGSFITDKFLLPSRPFYGNNWTVEARFTNGLETETVVTFEVKEYVLPKFSITLKSEPDIILPTTETIRINVSANYFHGKAI<br>DGTCVLSYGIITDGQHKISDVDLKQQMTVGSTDFEISKNTLEERFPSYWFPDGARLYLEVTILDKGTGRREQVSDKKTVFSNH<br>PYKISVEKSQKYFKPGIPYDLVIDTFYANGKPAPNCPLSIHCGKRTTNGNVIPIRHVRRLNVESDETGRIVKPIHIENRNTALSCT<br>IKTDADRNDYTYSVQSKVTLVVEAMQSPSNRFLSVKPMLNDGRLVAQVSTRRLRSNEFYHLLISGGRILSVLQSRGSNSFLQA<br>INSDMAPNLRIVTYAFNQRWGGATEVAADSATIEVVPSCHYQELEIMVPNTEVSPKSVLTINVTGPPLSKVGFLAIDKAVYQIS<br>DKNRLKKEKMFSSFGDHDLGCSVGGGINVNKVFEDAGLSVLTNAEIQTSQRDSVKCPTTGRRKRSINPSEETNPCCQAGRRF<br>AFQFGQTGLLRASDCYTEGQKLQKLIKSSKCAKAFYRCCKSYEDRLSDTQSIQPHAMKENRGNFFDFDRTVVMSQEEEYTFR<br>DINMMTEDVPIRSFFPESWLFEENSLDASGALTRSIIMPDSTTTHVLQAVSLSPLYGMCVAQSVDVISTKDFFVDLDLPYSVVR<br>MEQTEIRATLHNYRSIKLPCHVRLQNTEGVCSTEQDTIPIEIEPNNVQLVRFPIVPMRAGEFTIDVIVLCYDVGDRVKKTLRVV<br>NEGREEKKTVSFWLDPKGQRKHYHDESDLTVQVSHPFHSLDTQLTTVDLALPEEAIPGTGQCKVSAIGNIMDAAVQTVLGG<br>VGSLLDNIPHGCGEQTMILMAPLVYAMRYLHGTTGLTPQAENRGKFFMKVGYQRELAFRKQDGSFSVWQHRPSSTWLTAF<br>VIKVFCQAKTYIDIDQNIICTGMQWLFTKQNQNGSFTETSPVYHKEIMGGINGETSLTAFVLISLLECDCRLQGTSENIRKAVG<br>FLENQFLSTHHPYSLSIAAYALALANSNRKHRINRKLQRYSKFVNFPDERNKGYRYWPIEFHDHRNQTDIPYWYRKNPSAVSI<br>ETTAYALLAHLELDKITYSHNIVEWLVEQQQASGAFVSTQDTIVGLQALSMYNVRTYAEDVNMRCTITSGHNSTFREHLNLQ<br>ERESMVEKSVHDIPATGKLHVTTQGRGVARMEVEFRYNVNDTERTGCKFDINIEIDDIDVDILQEKQSQRDQQCDVCGKCED<br>VIDYDVVLDRYNVPDPDPRIGFSNQNVNPPPDRVQGPRPPSGPSGPDLLNVDLSGEVDIETSRGRSGRGRSGRGQSGRGQSGR<br>RRINGRRSNRRRTGRSARRRGASRQQKCMEICVSYMGNEILDMPVIDVGLPTGYVVEEEDLKKVTEEDFIDSYDVSKRAITFY<br>LQKIPQEEICFKLRMIMDFEVENLQSAKVEVYDYSKKDERCVKFYSLQYEVAELDVFCSKENCHCVEGECAEIWDNKLKGSL<br>IDASAVYDMTCHGYDYVLRIKVSGARIAGNHIVISTVIQSVINTVPDRSDLTVDSEVDFWINLRCTIELLESGHYYIIYGNGGY<br>HYIEESGTERYRYYLQGDSIIMKDYTREMYNPEAVAHLSYWKRFFTRMETHIRTNGC
**CrC3**	MESRILLFTLTVIFLCIRIEAANIFVASPNTLRIGEQETISVLLEGNKAETVEVYLQDHPGKTKTFSKTVGRVEPRISTEFKVQVN<br>PEDLPDKDILVATAKHYVSLVAKAGNWFHKETLLLVNPRSGYVFIQTDKPIYTPKQTVHMRIITLNEDLIPQNKMITLQIKNPQ<br>DITVEHHSWKVGKVGRRIEFYTFDYSFPPYPLFGEWSAIVSYGYDLLYNTTVKFEVKEYVLPTFSVDVTAPEIILESTKKIIGSA<br>HAKYVYGEPVHGTANFKFGVKLNTGDIIIIGTLYNKQLQDGKVDYQIDVNEFMKHGKISGFPDLEGNHLFVEVSVLEQATGQ<br>RESGINENGIFTLSPYDVSFKRCLNNFMPGYTTLIPVDINFVSGKPAPGIPAVITVTDEKGRAVVVQKSEDTSDERGRCNFLVN<br>PSKDLKEIKIEVKTNDGRGAQYQRKGQHRMGEQSSEFGGVIAIDRGTTKKDLKVNEEFSASVLTNPAGGISLISYMVISRGKIL<br>IHKFLPKVELIGHKIFFVVNTDMSPSFRLVVYAVYKGHLLTDSILYNVEPTCKESVKFKLETDVIDRPKPGQLVKINIVEATKD<br>TKIGLLAVDEAVYILRNKDRLTREKMFQEMEKHDLGCGPGGGSNIQSVLANAGVAILSSTTLTNYKREDYICAVRIRKKREIM<br>EEIVKKYEKRDKQCCILGMKHDPDQRSCEERHAIFEKYDFDGKETCMAAFLGCCNEKHLYLLKNIEKEGRGRFGFDPADQLI<br>TVGLEEEQELLKQLNVRKDFRETWIFDDVYVGPKGRVEKELSLPHSITTWVVQAVGISNTGGMCIAEPLKITTFKSIFVQLNIP<br>YSVVRNEQVEIQATVFNNHAHQSVRASVYMYGVKGLCSGAEEGQRTERKVLDIGRSSAKSVSFPVVPLKVGEFPVRVVVFT<br>VHGSDFIEKKLNVVPEGAKDSKLISLQLDPTNQQKRQKRSIHEKYYIDSIDPEKKMQISIVELQPPPNYVPDTAKCLVSVIADRF<br>GPVVETALENTEKLIQHPRGCGEQTMLFMAPTLYTVKYLKVTGQLNANTEKNGYKFIRDGYSRELTFRKNDGSYAAWQNRP<br>SSTWLTAFVMRVFCQAQKLIEIDEKVICSGMQWLVQRQKPDGSFVDEKPVIHQEMIGGVKGALPMTAFVLMALHECSCTTV<br>PGLKLAKIRAAAYLEIKVPHIQDPYIMSLVAYSLSLAGNNAKVEANRKLLSMATFQADKNYHYWGDPNSPRAIETAGYGLL<br>VQILNNDIEYANSIVNWLNSKRTLSGAFKSTQDTVIALYALSEYSILAKKPETDLQCNVTLNNDPSFFKELHFKESTANILQQF<br>QISNLGGHLIFNTTGYGMGQLAVELKYNVPVPPEKLCKFDIDVKVNEVKEEIQQVIDPRMPGNDVFDLLPDALLRNLGFPKR<br>KERSLSDNIQDYARVKRDNRGRVGDGGRRGNDGKSKLLLEIQICVKYLSHVDSNMAIIEAGIFTGFKVLIDELKQLVKEKNSK<br>IARFEASDKSVVFYMDSAPHDKPYCFKFRTVRQFIVGNIQSSVVKVYDYYKPNESCSQFYSPDNQSPLIRTICEGSVCQCAEGG<br>CPPRHPFQGVTQIHDISESRKLLLDRACVDHDYVWKGTVESKRKENGFRYISFRVTSVFKEGIEQKQNILHTSKDLMVRDSCS<br>VADLDIQQEYVIMGRDGAQFKDEDTGILLYRYILDQSTSIFKWTRISVAENKQLTKAFRWLEKHMVMGEGGCPQ
**HaC3-1**	MDMWSCAVFLLVVGVFGHTCAQQIYVVAPNTLRLNSDETIAVAIDGNIGAVVSVFVQDHPGKVKNISQTLVAVQPGQPELF<br>KIQLNSQNFPPNFLSGPGFPKYVSLTAVFPDFRKELIIPVSNQSGYIFIQTDKPIYTPKERASIRIIPLNEDSRPSEERFLLQIRNPKN<br>IIVEEKYFNDKNRKLDKAFASHVYRFPTYPVLGEWSATVRYGHDLEQNATVRFELEEYVLPTFTVELKVPDVVLPKDETIQIE<br>VKANYVYGKKVKGIVTFRLGVKGDHSPDAVFFAVITPKELDDGSYVLRIKTDDLRRHKDIGWFPEIEGSHLVVEATVADAAT<br>GNKETTIDSKGRFSKTPFLISFKRCLKDFKPGLTSVFEADVTYVDGSPAGGVRTKIQAVADNGKKLFIAVSSAQSDENGKVSF<br>EIQPELHHKSVTITLETDDPRYEGSQAKGIFEQHAYQSGQDAYIAIARSSPQKLKPGHMYGKEIHFHPAGIQDIYYMVTARGKI<br>LSMNKLPSGAYKNQRVEFQIGHDMVPSFRVVVFAHHKDELIADSLKIDVERECNPEVQVAVTPEFGEKEPGNNGKIVIRGTK<br>GTYVGLLGVDEAVYALSKKDILTKAKVFNKLATHDLGCGPGGGITVNSVLGNAGVTIGTRVFSPTESHSCVEIKRRKREAAM<br>DIVKTYMGTDRYCCSLGLSEDKYRRTCQERSNVVQKYLDGEYPTCAKAFMECCIYGLRNGLADMKVMRQGIALGRMGGE<br>NLEEPEFIDAEFEEDFEKQLTVRKDFRETWIYEDVTIGPDNREELGVSLPHSITTWVLQAVSVSPTHGICVAEPQKLVSFKKIFL<br>HLNLPYSVVRNEQVEIQATVFNYGNTKIGAVVYMYGAKDLCSGTQAGEKSERKRLIIEGQSAATVTFPVIPLKAEDFVIKVVA<br>LTPAGSDVVQRTLHVVAEGVTKEIDIPIKLDPTNQQRRQKRHIETELYSDHIDPTQNLQVTAVKLSAPEGFVPGTASCSITALG<br>DMYGPAVQTSINNPDALFQKPRGCGEQNMMYLAPTLYALRYLKVTGKLTAAAEESGYEFIRHGYGNQLAFRKEDGSYAAY<br>QSKPTSTWLTAFVIKVFCQATELIHIDDDVVCSGVKWLIKNQERDGSYVEKHPMYHVDMMGGVQGKTPMTAFALIALEECK<br>CDEEHLLLSKKRAVAYLENHLGEVHTPLAVAVVAYALSLSDSELRQVANDKLLKLAKYDEDTNRMYWNTENSAQDIETAG<br>YALLNQLLFNDMSRSNSIVNWLNTKQLQSGSFKSTQDTVVALQAMSEYAIRAQMPSINLVANISSSNDRNFHKVMAFRDDN<br>ALVLQDVRIDKIGGTVFINTAGHGMGSLSVKLRYNLNVPVEDICKFDVKVNVTESKPEQQEKDPWDEVLNQKGKDMFERFP<br>EDLIRGIKKELDEVPPELAIDPRRRGHVRASLIASPLIKKDRQKRQKPNENSKVKLNITICVRYLGNKDTEMSIVDAGIFSGFQP<br>VEDDLIWLQDDHSHLIQRYEKSSRGVVFYLQKVPIAGDYCFSFHVVRQYIVGNTQTSVIKVYDYYNPDATCTKFYSPGSNSP<br>MLRTICEGGICECAEGGCPPNNPFSEISSMSTAQRREELKTIFCENYDYVWKGKLNGRLQKDGGFLNISFTIADVIKAGIEKAE<br>DIVDETRTLLARDNCPTANLFPGVTYLILGKDGQNYMNENGETWYRYLLDKTSVIHMWTSAKEAKNKNLQRDLNSVTKAL<br>KENGCEL
**EsC3**	MPFLASRQANIIFACFLLTSCVIQTISSSKVLVVAPKLFRCGEEERILISAFGINSRIPFQVLLEHSTHGNVISDQKIYVSEDQPGM<br>ANLTVTLGDLAHRMSSSSDVGEESHFVNLVITSDIFRQTTEIPVELNTGFIFIQTDKPIYNPSQTVKIRIVSLNEHMLPAKSPITFS<br>IQNPRGLILYSHQFKVIRPFVNTTYELTAHPPLGNWTITAGFANGMMTRTKTKFVVQKYMLPVFEVKIQVKPQFIVRSTQIVT<br>VTTTAKYLYGKPVKGKVKLCYSYGLLALLKREIFQELVDGQIVTNIRLPPNQVPLGTPLNVTAEVVETNSGISETKEDTSAKF<br>VFSPYKFDVSDSQTFFKPGLPYTVNVETLYANGSSASNIPFYLEYIAKTRSNRQEHFKQLYKTITDFKQCVIQTSRFWTQFTITL<br>TTADQRLDSTDQIQEKWLVSASKSETDSYLLLSLNTTSDGFTVGDFVTMQASTSRIFFKTKLYYMIIGKSKILKYGVLPYNRD<br>GWRINFRVTGDMIPVGRVIAYYARRKEVVADSRSFKVVDICGSPELQLATSPKRPLHHPMTNVEIQLKGSPHATVGLSAVDK<br>AVYLINDKNRLTRKLVKDTMKLGDLACSPGGGLNVGQVFYDAGFRIITEGLSRTPVAESNNMLCSLRKLPAEVTFGRYKRGS<br>DFCCMSGELNRGSNCKIAWLQFTYRKTEACRIAFMQCCKQDAVMPPTDRVITEGPVEKPDVGHVPIARSRQQDNSISPVFSL<br>YFPESELFQEFDLNQSGQHNLNFTLPDSLTTWHIQGLSLSDKSPFCVSKPISVTTFKDVITKLNFPHVVKRLEQIHIKAVVYNYR<br>TYPINVKVHFHETKGICSHAAPQKPFTVKLNILAQDIGVAKLVIVPLRAGNFTIHIEVYEVESNTLEIIEKVLHVKYEGVKKRKS<br>LSLMLDPSGIYSRHTRIRRHLFTDVTIPKNEISISQRTQRIIMNLTQPAEAIEGSAKGDIVITGNILGSTIQTTLKEPQKLLEKPYGC<br>GEQTMIHLAPCVYILSYLNSTKQGSAEIYENATRYIRLGVLREQEFRKRDGSYAAFDQNPSSVWLTAFVLKVFCQASEYEHID<br>SHLTKSAMTWLFQQQRLDGSFVEDNPVIHQEMVGGVGDKGMTAFVLISLMECGYRMHPEEIYRRKNEVNLAIRHIENQME<br>MTDNSYDMAIISYALALSESSKKYEAHRKLRSMAVENYGKNYLYWKVDAFSNMTDEKVSAWYHKEESYLSVEATAYALL<br>TELHYGEVESAGPIVKWLIEQRNSAGMFKSTQDTVMALQALAEYGIKTITPDINLFFNLTSSRAHLHKHLEVNKETSLWSHVI<br>SDVPINDEVLIESMGIGSAQMELNMWYYRPPTAREVCHFNITHVITPVRKETRRLIKRGCPLCGESCPKMNGPILRNWNVNGQ<br>TICMKIYLRYLGASNSGMVVLDMDILTGYDLHEELLDQLKQDRVIAKHYKHDNSKLTLYFNQVPNDRELEIKLLLFRSVSTT<br>NRLMPAPIHVFVYYNATESCTKFYQLSREKDLINLLCDSDKTICKCAQRECVACYESVSQLLEINLSFLERKACIKDQIVMIVN<br>MTSSFDDTGTAHGFVLHDLSKTNFQELENHHAIFQRGMCYCPRVNVGEISLVMSNKRVRTMNEFNQFKHKFWVDSSGLMIK<br>KSHEFIKKHTDIFESKAKRISRKDFCGHF

**MmC3**	MGPASGSQLLVLLLLLASSPLALGIPMYSIITPNVLRLESEETIVLEAHDAQGDIPVTVTVQDFLKRQVLTSEKTVLTGASGHL<br>RSVSIKIPASKEFNSDKEGHKYVTVVANFGETVVEKAVMVSFQSGYLFIQTDKTIYTPGSTVLYRIFTVDNNLLPVGKTVVILI<br>ETPDGIPVKRDILSSNNQHGILPLSWNIPELVNMGQWKIRAFYEHAPKQIFSAEFEVKEYVLPSFEVRVEPTETFYYIDDPNGLE<br>VSIIAKFLYGKNVDGTAFVIFGVQDGDKKISLAHSLTRVVIEDGVGDAVLTRKVLMEGVRPSNADALVGKSLYVSVTVILHS<br>GSDMVEAERSGIPIVTSPYQIHFTKTPKFFKPAMPFDLMVFVTNPDGSPASKVLVVTQGSNAKALTQDDGVAKLSINTPNSRQ<br>PLTITVRTKKDTLPESRQATKTMEAHPYSTMHNSNNYLHLSVSRMELKPGDNLNVNFHLRTDPGHEAKIRYYTYLVMNKGK<br>LLKAGRQVREPGQDLVVLSLPITPEFIPSFRLVAYYTLIGASGQREVVADSVWVDVKDSCIGTLVVKGDPRDNHLAPGQQTTL<br>RIEGNQGARVGLVAVDKGVFVLNKKNKLTQSKIWDVVEKADIGCTPGSGKNYAGVFMDAGLAFKTSQGLQTEQRADLECT<br>KPAARRRRSVQLMERRMDKAGQYTDKGLRKCCEDGMRDIPMRYSCQRRARLITQGENCIKAFIDCCNHITKLREQHRRDHV<br>LGLARSELEEDIIPEEDIISRSHFPQSWLWTIEELKEPEKNGISTKVMNIFLKDSITTWEILAVSLSDKKGICVADPYEIRVMQDFF<br>IDLRLPYSVVRNEQVEIRAVLFNYREQEELKVRVELLHNPAFCSMATAKNRYFQTIKIPPKSSVAVPYVIVPLKIGQQEVEVKA<br>AVFNHFISDGVKKTLKVVPEGMRINKTVAIHTLDPEKLGQGGVQKVDVPAADLSDQVPDTDSETRIILQGSPVVQMAEDAVD<br>GERLKHLIVTPAGCGEQNMIGMTPTVIAVHYLDQTEQWEKFGIEKRQEALELIKKGYTQQLAFKQPSSAYAAFNNRPPSTWL<br>TAYVVKVFSLAANLIAIDSHVLCGAVKWLILEKQKPDGVFQEDGPVIHQEMIGGFRNAKEADVSLTAFVLIALQEARDICEGQ<br>VNSLPGSINKAGEYIEASYMNLQRPYTVAIAGYALALMNKLEEPYLGKFLNTAKDRNRWEEPDQQLYNVEATSYALLALLL<br>LKDFDSVPPVVRWLNEQRYYGGGYGSTQATFMVFQALAQYQTDVPDHKDLNMDVSFHLPSRSSATTFRLLWENGNLLRSE<br>ETKQNEAFSLTAKGKGRGTLSVVAVYHAKLKSKVTCKKFDLRVSIRPAPETAKKPEEAKNTMFLEICTKYLGDVDATMSILD<br>ISMMTGFAPDTKDLELLASGVDRYISKYEMNKAFSNKNTLIIYLEKISHTEEDCLTFKVHQYFNVGLIQPGSVKVYSYYNLEE<br>SCTRFYHPEKDDGMLSKLCHSEMCRCAEENCFMQQSQEKINLNVRLDKACEPGVDYVYKTELTNIELLDDFDEYTMTIQQVI<br>KSGSDEVQAGQQRKFISHIKCRNALKLQKGKKYLMWGLSSDLWGEKPNTSYIIGKDTWVEHWPEAEECQDQKYQKQCEEL<br>GAFTESMVVYGCPN
**CcC3 H1**	MEVKLLFLTVVLLSSPLLTLCNPLYVLSAPNLLRVGSSENVFVEAQDYSGAAFDVKIIVKNHPKKDKEILSQSVSLTAANNFQI<br>LKDIKIPDDQNYFSDDPLEKQYVYLQAHFPSVILEKVVLLSFQSGYIFVQTDKPIYTPASTVQYRIFSLTPNLEPLSQSGITVEIM<br>NPQGITVSSEKIFPVKGMKSGKYAIPEMASPGIWKVVTLFSNTPQKTFTADFEVKEYVLPTFEVKLKPSKSFFYVHDESLTVDI<br>EAKYLFGQKVDGNAFVMFGVMEDEKKTSIPASLQKVQIIKGEGTAELTNQMITKTFPNINQLVGRSIYVSVSLLTESGSEMVE<br>AERRGIQIVTSPYTIHFRKTPQFFKPGMPFDVSVYVTNPDQTPAVNVEVEVNPGGLRGQTRANGIAKVTVNTPGGSPTLAITA<br>KTKDPEIKDERQQAEKRMTAQAYIPKGGSNNYLHIGIDAAELQISDPMKVNLNTGQSPGVKDQDYTYMILSKGQIVKVDRFK<br>RKGQSLVTLPVTVTKDMVPSFRFVAYYHVGLEVVSDSVWVDVKDTCMGKLQIKVKNKMNTYGTGDEVKLQITGDPGAKV<br>GLVVVDKAVQVLNKNRLTQTQIWDVIEKHDTGCTAGGGKDSMGVFTDAGLMFESNGAGGTNTRTMPECPKTSKRKRRAES<br>LLQITSTLAGKYTGELKQCCVDGMRDNKLGYTCERRATYIVDGEECAKAFVDCCNKIKDRKNTETEEEEMLLARSDDDDDY<br>YTESEEIVSRTQFPESWLWEEIDLCDKCPTPATEKVIYLKDSITTWEILAVSLSPTLGICVAEPEEMVVFKHLFIDLKMPYSAVR<br>GEQLEIRAIIHNYTPNKQKVRVEFMETEDVCSFASKKGKYRTTVSVEKDSSISVSYVIIPMTLGNHMIEVKASAYDAIYTDGVR<br>KPLKVVSEGVLIPLHRKNVELNPVKTGEKPIVLKSDIPVDRVPGTPANTYISITGEEIAQTVEQAISGDFMGRLIIQPNGCGEQI<br>MIGMTLPVIATHYLDSTNQWETVGMERRNEAINHINAGYQRQLGYRKSDGSYAAWTHRPSSTWLTAYVAKVFSMANDFAA<br>IEENVLCSALKWLVLHKQLPDGSFKEESAVIHGEMVGDVRGKDADASLTAFVVIAMQEAREICGGSVGSLHDSIKKAVVFLE<br>GRLPQLTNPYAVAMTSYAMANAGKLNKDILMKYSSQHEAGRSWTVPGQHHHSLEATAYAVLALVKAKDFDKAGEAVHW<br>LNRQKSHYGGSGTTQATIMVFQAVAEYRTQVKDRQNFNLDVELSVAGRGKPVRYTIKRENAHLTRSDRVDINKEFNVTARG<br>TGTATLSVLTLYYARPVEKKSDCTFFDLTVKMEKDNEANQGAIETYKITMDFYYKSDKTDATMTILDIGIPTGFTVENRDLEE<br>LSRGKERYIQKFEMDKVLSERGSLILYLDKVLRKEPQRLAFRMHKMLNVGVLQPAAVTIYEYYSPNARCTKFFHPERADGAI<br>SRLCKGDLCQCAEENCSYQKKNHIGDEERFNRACEAGMDYVYKVKVVGMDLQQDADIYDLEVEQVLKEGTDVDVEGKRR<br>PFLARPTCRNHLGLVKDKSYLIMGRSVDLPDLGGSLQYIFGEQTWIEYWPTRQESQTREHRDRYIGISDLQNSLLKEGCAT
**HsC4**	MRLLWGLIWASSFFTLSLQKPRLLLFSPSVVHLGVPLSVGVQLQDVPRGQVVKGSVFLRNPSRNNVPCSPKVDFTLSSERDFA<br>LLSLQVPLKDAKSCGLHQLLRGPEVQLVAHSPWLKDSLSRTTNIQGINLLFSSRRGHLFLQTDQPIYNPGQRVRYRVFALDQK<br>MRPSTDTITVMVENSHGLRVRKKEVYMPSSIFQDDFVIPDISEPGTWKISARFSDGLESNSSTQFEVKKYVLPNFEVKITPGKP<br>YILTVPGHLDEMQLDIQARYIYGKPVQGVAYVRFGLLDEDGKKTFFRGLESQTKLVNGQSHISLSKAEFQDALEKLNMGITD<br>LQGLRLYVAAAIIESPGGEMEEAELTSWYFVSSPFSLDLSKTKRHLVPGAPFLLQALVREMSGSPASGIPVKVSATVSSPGSVP<br>EVQDIQQNTDGSGQVSIPIIIPQTISELQLSVSAGSPHPAIARLTVAAPPSGGPGFLSIERPDSRPPRVGDTLNLNLRAVGSGATFS<br>HYYYMILSRGQIVFMNREPKRTLTSVSVFVDHHLAPSFYFVAFYYHGDHPVANSLRVDVQAGACEGKLELSVDGAKQYRN<br>GESVKLHLETDSLALVALGALDTALYAAGSKSHKPLNMGKVFEAMNSYDLGCGPGGGDSALQVFQAAGLAFSDGDQWTLS<br>RKRLSCPKEKTTRKKRNVNFQKAINEKLGQYASPTAKRCCQDGVTRLPMMRSCEQRAARVQQPDCREPFLSCCQFAESLRK<br>KSRDKGQAGLQRALEILQEEDLIDEDDIPVRSFFPENWLWRVETVDRFQILTLWLPDSLTTWEIHGLSLSKTKGLCVATPVQL<br>RVFREFHLHLRLPMSVRRFEQLELRPVLYNYLDKNLTVSVHVSPVEGLCLAGGGGLAQQVLVPAGSARPVAFSVVPTAATA<br>VSLKVVARGSFEFPVGDAVSKVLQIEKEGAIHREELVYELNPLDHRGRTLEIPGNSDPNMIPDGDFNSYVRVTASDPLDTLGS<br>EGALSPGGVASLLRLPRGCGEQTMIYLAPTLAASRYLDKTEQWSTLPPETKDHAVDLIQKGYMRIQQFRKADGSYAAWLSR<br>GSSTWLTAFVLKVLSLAQEQVGGSPEKLQETSNWLLSQQQADGSFQDLSPVIHRSMQGGLVGNDETVALTAFVTIALHHGL<br>AVFQDEGAEPLKQRVEASISKASSFLGEKASAGLLGAHAAAITAYALTLTKAPADLRGVAHNNLMAMAQETGDNLYWGSV<br>TGSQSNAVSPTPAPRNPSDPMPQAPALWIETTAYALLHLLLHEGKAEMADQAAAWLTRQGSFQGGFRSTQDTVIALDALSA<br>YWIASHTTEERGLNVTLSSTGRNGFKSHALQLNNRQIRGLEEELQFSLGSKINVKVGGNSKGTLKVLRTYNVLDMKNTTCQD<br>LQIEVTVKGHVEYTMEANEDYEDYEYDELPAKDDPDAPLQPVTPLQLFEGRRNRRRREAPKVVEEQESRVHYTVCIWRNGK<br>VGLSGMAIADVTLLSGFHALRADLEKLTSLSDRYVSHFETEGPHVLLYFDSVPTSRECVGFEAVQEVPVGLVQPASATLYDY<br>YNPERRCSVFYGAPSKSRLLATLCSAEVCQCAEGKCPRQRRALERGLQDEDGYRMKFACYYPRVEYGFQVKVLREDSRAAF<br>RLFETKITQVLHFTKDVKAAANQMRNFLVRASCRLRLEPGKEYLIMGLDGATYDLEGHPQYLLDSNSWIEEMPSERLCRSTR<br>QRAACAQLNDFLQEYGTQGCQV
**HsC5**	MGLLGILCFLIFLGKTWGQEQTYVISAPKIFRVGASENIVIQVYGYTEAFDATISIKSYPDKKFSYSSGHVHLSSENKFQNSAILT<br>IQPKQLPGGQNPVSYVYLEVVSKHFSKSKRMPITYDNGFLFIHTDKPVYTPDQSVKVRVYSLNDDLKPAKRETVLTFIDPEGS<br>EVDMVEEIDHIGIISFPDFKIPSNPRYGMWTIKAKYKEDFSTTGTAYFEVKEYVLPHFSVSIEPEYNFIGYKNFKNFEITIKARYF<br>YNKVVTEADVYITFGIREDLKDDQKEMMQTAMQNTMLINGIAQVTFDSETAVKELSYYSLEDLNNKYLYIAVTVIESTGGFS<br>EEAEIPGIKYVLSPYKLNLVATPLFLKPGIPYPIKVQVKDSLDQLVGGVPVILNAQTIDVNQETSDLDPSKSVTRVDDGVASFV<br>LNLPSGVTVLEFNVKTDAPDLPEENQAREGYRAIAYSSLSQSYLYIDWTDNHKALLVGEHLNIIVTPKSPYIDKITHYNYLILS<br>KGKIIHFGTREKFSDASYQSINIPVTQNMVPSSRLLVYYIVTGEQTAELVSDSVWLNIEEKCGNQLQVHLSPDADAYSPGQTVS<br>LNMATGMDSWVALAAVDSAVYGVQRGAKKPLERVFQFLEKSDLGCGAGGGLNNANVFHLAGLTFLTNANADDSQENDEP<br>CKEILRPRRTLQKKIEEIAAKYKHSVVKKCCYDGACVNNDETCEQRAARISLGPRCIKAFTECCVVASQLRANISHKDMQLG<br>RLHMKTLLPVSKPEIRSYFPESWLWEVHLVPRRKQLQFALPDSLTTWEIQGIGISNTGICVADTVKAKVFKDVFLEMNIPYSV<br>VRGEQIQLKGTVYNYRTSGMQFCVKMSAVEGICTSESPVIDHQGTKSSKCVRQKVEGSSSHLVTFTVLPLEIGLHNINFSLET<br>WFGKEILVKTLRVVPEGVKRESYSGVTLDPRGIYGTISRRKEFPYRIPLDLVPKTEIKRILSVKGLLVGEILSAVLSQEGINILTH<br>LPKGSAEAELMSVVPVFYVFHYLETGNHWNIFHSDPLIEKQKLKKKLKEGMLSIMSYRNADYSYSVWKGGSASTWLTAFAL<br>RVLGQVNKYVEQNQNSICNSLLWLVENYQLDNGSFKENSQYQPIKLQGTLPVEARENSLYLTAFTVIGIRKAFDICPLVKIDT<br>ALIKADNFLLENTLPAQSTFTLAISAYALSLGDKTHPQFRSIVSALKREALVKGNPPIYRFWKDNLQHKDSSVPNTGTARMVE<br>TTAYALLTSLNLKDINYVNPVIKWLSEEQRYGGGFYSTQDTINAIEGLTEYSLLVKQLRLSMDIDVSYKHKGALHNYKMTDK<br>NFLGRPVEVLLNDDLIVSTGFGSGLATVHVTTVVHKTSTSEEVCSFYLKIDTQDIEASHYRGYGNSDYKRIVACASYKPSREE<br>SSSGSSHAVMDISLPTGISANEEDLKALVEGVDQLFTDYQIKDGHVILQLNSIPSSDFLCVRFRIFELFEVGFLSPATFTVYEYHR<br>PDKQCTMFYSTSNIKIQKVCEGAACKCVEADCGQMQEELDLTISAETRKQTACKPEIAYAYKVSITSITVENVFVKYKATLLD<br>IYKTGEAVAEKDSEITFIKKVTCTNAELVKGRQYLIMGKEALQIKYNFSFRYIYPLDSLTWIEYWPRDTTCSSCQAFLANLDEF<br>AEDIFLNGC

**MmC5**	MGLWGILCLLIFLDKTWGQEQTYVISAPKILRVGSSENVVIQVHGYTEAFDATLSLKSYPDKKVTFSSGYVNLSPENKFQNAA<br>LLTLQPNQVPREESPVSHVYLEVVSKHFSKSKKIPITYNNGILFIHTDKPVYTPDQSVKIRVYSLGDDLKPAKRETVLTFIDPEG<br>SEVDIVEENDYTGIISFPDFKIPSNPKYGVWTIKANYKKDFTTTGTAYFEIKEYVLPRFSVSIELERTFIGYKNFKNFEITVKARY<br>FYNKVVPDAEVYAFFGLREDIKDEEKQMMHKATQAAKLVDGVAQISFDSETAVKELSYNSLEDLNNKYLYIAVTVTESSGG<br>FSEEAEIPGVKYVLSPYTLNLVATPLFVKPGIPFSIKAQVKDSLEQAVGGVPVTLMAQTVDVNQETSDLETKRSITHDTDGVA<br>VFVLNLPSNVTVLKFEIRTDDPELPEENQASKEYEAVAYSSLSQSYIYIAWTENYKPMLVGEYLNIMVTPKSPYIDKITHYNYL<br>ILSKGKIVQYGTREKLFSSTYQNINIPVTQNMVPSARLLVYYIVTGEQTAELVADAVWINIEEKCGNQLQVHLSPDEYVYSPG<br>QTVSLDMVTEADSWVALSAVDRAVYKVQGNAKRAMQRVFQALDEKSDLGCGAGGGHDNADVFHLAGLTFLTNANADDS<br>HYRDDSCKEILRSKRNLHLLRQKIEEQAAKYKHSVPKKCCYDGARVNFYETCEERVARVTIGPLCIRAFNECCTIANKIRKES<br>PHKPVQLGRIHIKTLLPVMKADIRSYFPESWLWEIHRVPKRKQLQVTLPDSLTTWEIQGIGISDNGICVADTLKAKVFKEVFLE<br>MNIPYSVVRGEQIQLKGTVYNYMTSGTKFCVKMSAVEGICTSGSSAASLHTSRPSRCVFQRIEGSSSHLVTFTLLPLEIGLHSIN<br>FSLETSFGKDILVKTLRVVPEGVKRESYAGVILDPKGIRGIVNRRKEFPYRIPLDLVPKTKVERILSVKGLLVGEFLSTVLSKEGI<br>NILTHLPKGSAEAELMSIAPVFYVFHYLEAGNHWNIFYPDTLSKRQSLEKKIKQGVVSVMSYRNADYSYSMWKGASASTWL<br>TAFALRVLGQVAKYVKQDENSICNSLLWLVEKCQLENGSFKENSQYLPIKLQGTLPAEAQEKTLYLTAFSVIGIRKAVDICPT<br>MKIHTALDKADSFLLENTLPSKSTFTLAIVAYALSLGDRTHPRFRLIVSALRKEAFVKGDPPIYRYWRDTLKRPDSSVPSSGTA<br>GMVETTAYALLASLKLKDMNYANPIIKWLSEEQRYGGGFYSTQDTINAIEGLTEYSLLLKQIHLDMDINVAYKHEGDFHKYK<br>VTEKHFLGRPVEVSLNDDLVVSTGYSSGLATVYVKTVVHKISVSEEFCSFYLKIDTQDIEASSHFRLSDSGFKRIIACASYKPSK<br>EESTSGSSHAVMDISLPTGIGANEEDLRALVEGVDQLLTDYQIKDGHVILQLNSIPSRDFLCVRFRIFELFQVGFLNPATFTVYE<br>YHRPDKQCTMIYSISDTRLQKVCEGAACTCVEADCAQLQAEVDLAISADSRKEKACKPETAYAYKVRITSATEENVFVKYTA<br>TLLVTYKTGEAADENSEVTFIKKMSCTNANLVKGKQYLIMGKEVLQIKHNFSFKYIYPLDSSTWIEYWPTDTTCPSCQAFVEN<br>LNNFAEDLFLNSCE
**EtTEP**	MRALLVLAVAIAAAWADNSYVVISPSKIRANMDLSLSVNILKATADVTVTASILRGTTSVATGTGVFKAGTPDSLTLKIPQGL<br>PNAGYTLSVVGTGGLIFNNSTPLTFNSKELSLFIQSDKAIYKPGDTVDFRAFAVYADLKSYTGPIDISVFDAAANKIKQWLQVK<br>PTDGVITQNLTLSSQPVLGDWRIHVETGDSNVEKVFTVAEYVLPKFEVDVVMPSYSLTTDTDITFTVKAKYTYGKPVTGTAD<br>VSVKLNYDSTLYDYSRALPVSAVHVPVDGEAKVTIPMADVKRIRTSLNGNVLIVTANVSESLTGNTMSGNGTVKLYDKGVT<br>LEFPKTNPNTFKPGLKYTAYLKVSQPDGLPVSTSTQTVNVNTDVVIELPGITPTPYFYYQDPTESRKLPVALYDVPDNGLVEV<br>PVDIPNDAKSATITATFQGVSATLSLGKSHSPSNSYIQLFLETTSAIQAGDSIPFRVKATQPITSLVYQVLSRGGIVRTGSFNTNG<br>QSEYTFSVKSDSSMAPNARIVVYYVRTDGEIVTDSISFDVGGAFRNKVSISVNTTDAEPGDKVTVTVQADPPSTAHLLAIDQS<br>VLLLKSGNDVSADDVYNELKAYDTIDSNSNVFPIFDDCPVCRRKRLLIWWPRPIFFGGNDATQIFQNSGVKVITDALVYHYQE<br>PYPDYIFDKGGLIFEMAPGMPVPTAASGHGTETSNGDLQEPARTRSNFAETWLWLETSIGTNGTASISTQVPDTITPWVAGAF<br>AVNSVTGLGVVPTQTHLRVFRPFFVSLNLPYSVTRGEQLALQAIVFNYMSDDMQVRVTLAKSSSFFNIIINANSQQELKQEDA<br>VQDILVAAGDAKSVYFPIVPSDLGRIDVEVKAQSTRAADAVRRQLLVEAEGVPKEYNVPMLIDLTNGKNTFTETASLTLPAT<br>TVKGSELARVTAVGDMMGPTISGLDSLLQMPTGCGEQTMLGLAPDVYVTDYLKSVNQLTGDIKSKALGYMESGYQRELTY<br>KHKDGSFSAFGDSDKSGSMWLTAFVTRVFHQAKAHIYIDDSIIITALQWMIGHQNSDGSFPEPGNVIHKNMQGNAANGTALT<br>IFVLISLLENHDLLVNTNAAGYLLDQAAAKALAYSEKAVANTDDLYILAMAAYAFQLAGSTQTQAVLTKLEQKASVKDGSK<br>YWHQVEAPKTTNLGWESPNPTQAVGTEITSYVLLTYAAQGDLVKAKNVIQWITKQRNPHGGFQSTQDTVVALFAMSQFAK<br>QVYSNNFNVHVTATLSPTLTYNFNIDQTNALLLQSRETNTVPAQVKVDASGSGMALVEVAVFFNVESEIEETKFELKVTLLEE<br>TINHLVVETCTRWLGEGPSSGMAVQEIGIPSGFEVDLESMTQLPTLKRTETQNKKVILYFDQISTTPVCLNFKAERTGLVAKSQ<br>PVAVRVYDYYAPHNQVTAFYQSQILKDSSICDVCKDCEQCV
**AfTEP-D**	MLWVGLTTLLLGLAAAKDSYVVITPKDVRPGVSLNISVNILQAAGDVHVTAKLIHVADKSVKAFSTGTFQQHVPDTMQIMIP<br>DMIPSGTNQLTVEGSNGLTFSGKTNLHYASKGMSVFIQTDKAMYKPGQTVNFRAFAIFPNLTVYSGPLDIEIYDPNSNKIKQW<br>FGMKDSSGVITNFMAMDTKPVLGDWKIRVKTYGGLTKDKMFTVARYVLPKFEVTVDLPSYDWTTATSILGAVKAKYTYGK<br>PVNGTVKIRAHADFYHYNYYHPAPIPTIELTMDINGETKFTLPVSGLTSHTYYTSLNSRNVVVEANVTESLTQITLNGTGKMH<br>FYTHAEKIELLPSNPTTFKPGLQYIAYAKVVQQDDMPLAAGSSKSLTVHTSVTANLPETTTPLYYYGPRTMNYQLPDQSFTIT<br>DTGLVQAKIDIPDNATSISLQFKYGQITQYHSVQRSYSPSDSFIQIFLESNNLQAGHDKVVDFRVVSTSPIDKLVYQVLGRGSIA<br>VSGAINGNNAKAFPFNVPLNAKMAPNARIVAYYVRADGEIVTDSISFDVSGTFENDVSIRFDKSKAQPGDGINVDVSADPNSI<br>VNLLAVDQSVLLLKSGNDITPAEVVDELKSYDTIVHSNNNGPIFFGGGGIMPEPMPVGRRKRMIWWPFTTYYGGSDAEQIFQ<br>NAGVNVMTDALVYHHVEPHIYYPELLPTAAPAFGIMSVPGMAAATIAGGMVGTSVLKEVETIRSDFPETWLWLNKTVGAD<br>GHVTIAATVPDTITSWVASAFAVHPTSGLGIAPTSAKVEAFSPFFVSLTLPYSVVRGEQLVLQANVFNYMTTDMDVVVTLEK<br>NDDLVNVVFDTQGAESYIAQTTAKTVHVTAGGSKSVFFPVVPAGLGSVSINVKAQSTLAADAVRRQLLIEAEGVPKEYNIPM<br>LVDLKHNTNFAETVDVTLPAGVVAGSQRVRISAIGDLMGPTVNGLDKLLRMPTGCGEQTMLGFAPDVFVTNYLTDTHQLTS<br>SVEEKAINFMEKGYQRELTFQHKDGSFSAFGDNDPSGSMWLTAFVAKSFHQAKRHVFIDDETLTRAIDWMINRQAANGSFPE<br>PGRIIHKNMQGGSASGASLTAFVLIALLENSDLQGGVHMRIQSAASKAQAYLEGEVSAMTDPYGLSICSYALTLASSQSSATT<br>FQKLMAKAVTKDGMTHWHEPESAAPSTGHYWSPPHQQSKPVDIEMTSYGLMVFAHNSQFTEGLPFMKWITKQRNPNGGFS<br>STQDTVLALQSLSEFARIGYSEHFDMQIGIVAGQTTHTFSVTRQNALLLQSLELPSIPSHVTVTGTGSGMGLVEVSVFFNVEQE<br>VEQPSFEVDVTIMEETINSLKVRSCTKWLKTGASGMTVQEVGVPTGFAPDVESIGKIATLKKTETENRKVILYFDEITTTPLCV<br>TMNAYRTDQVAKSQPAPIRVYDYYEPSNQVTKFYQSTVLKNSGVCDLCKECGCHGQH
**AgTEP1**	MWQFIRSRILTVIIFIGAAHGLLVVGPKFIRANQEYTLVISNFNSQLSKVDLLLKLEGETDNGLSVLNVTKMVDVRRNMNRMI<br>NFNMPEELTAGNYKITIDGQRGFSFHKEAELVYLSKSISGLIQVDKPVFKPGDTVNFRVILLDTELKPPARVKSVYVTIRDPQR<br>NVIRKWSTAKLYAGVFESDLQIVPTPMLGVWNISVEVEGEELVSKTFEVKEYVLSTFDVQVMPSVIPLEEHQAVNLTIEANYH<br>FGKPVQGVAKVELYLDDDKLNQKKELTVYGKGQVELRFDNFAMDADQQDVRVKVSFIEQYTNRTVVKQSQITVYRYAYR<br>VELIKESPQFRPGLPFKCALQFTHHDGTPAKGITGKVEVSDVGFETTTTSDNDGLIKLELQPSEGTEQLGINFNAVDGFFFYED<br>VNKVETVTDAYIKLELKSPIKRNKLMRFMVTCTERMTFFVYYVMSKGNIIDAGFMRPNKQTKYLLQLNATEKMIPKAKILIA<br>TVAGRTVVYDYADLDFQELRNNFDLSIDEQEIKPGRQIELSMSGRPGAYVGLAAYDKALLLFNKNHDLFWEDIGQVFDGFH<br>AINENEFDIFHSLGLFARTLDDILFDSANEKTGRNALQSGKPIGKLVSYRTNFQESWLWKNVSIGRSGSRKLIEVVPDTTTSWY<br>LTGFSIDPVYGLGIIKKPIQFTTVQPFYIVENLPYSIKRGEAVVLQFTLFNNLGAEYIADVTLYNVANQTEFVGRPDTDLSYTKS<br>VSVPPKVGVPISFLIKARKLGEMAVRVKASIMLGHETDALEKVIRVMPESLAQPKMDTSFFCFDDYKNQTFPFNLDINKKAD<br>NGSKKIEFRLNPNLLTMVIKNLDNLLAVPTGCGEQNMVKFVPNILVLDYLYATGSKEQHLIDKATNLLRQGYQNQMRYRQT<br>DGSFGVWEKSGSSVFLTAFVATSMQTASKYMNDIDAAMVEKALDWLASKQHSSGRFDETGKVWHKDMQGGLRNGVALTS<br>YVLTALLENDIAKVKHAVVIQNGMNYLSNQLAFINNPYDLSIATYAMMLNGHTMKKEALDKLIDMSISDNNKKERYWGTT<br>NQIETTAYALLSFVMAEKYLDGIPVMNWLVNQRYVTGSFPRTQDTFVGLKALTKLAEKISPSRNDYTVQLKYKKNTKYFNIN<br>SEQIDVQNFLEIPEDTKKLEINVGGIGFGLLEVIYQFDLNLVNFEHRFKLDLEKQNTGSDYELRLRVCANYIPELTDSQSNMALI<br>EVTLPSGYVVDRNPISEQTTVNPIQNMEIRYGGTSVVLYYYKMGTERNCFTVTAYRRFKVALKRPAYVVVYDYYNTNLNAI<br>KVYEVDKQNVCEICEEEDCPAECKK
**DmTEP4**	MRRADAFAVSLCVILALLQTMEPVKAEGKYTIVGPGTIHSHRDYNVAVAVHQTKEPVTLKVGITGPSYNKTETVELATAGEF<br>KQITFKLPPLEAGEYNLTAEGVKGLEFKNSTKLNWENFKPYIKIQTDKGKYKPGDTINYRVIFLDENLRPDTAKDEVVVWFE<br>DSKRNRIKQEKHIKTTGGVYTGKFELSEFATLGSWSLHVQNGDQHHDGGIYFGGRKQFGGFGHRWHRSDELVNFEVEKYVL<br>PKYSVKMDATQQVSVRDGEFNVVLKANYTYGKPVNGKVLVNVHLDSTSSWENVDGKTVQTDYPGHSVVGTADMVGGKA<br>KLTMDLKDFASYLPHKTSSSYAQITATVEEDFTGVKLNETGGVQLYPYRYEMSCTDYSSCFSFKPDKEHELNFKITYVDGSLI<br>TDTKSVVKAKFTEGIRRNYAFYAFGTDHQEPELPTIEKKTFVFESHLNASGVAPFKVVLPDLPDIANFTRYYSIELEFVDEKRD<br>LYTTYPYREPKQIENPSSEEEKEWFRAEVQRPKDVWNLKIGQEYQVILNSSRPLKYFVYNIVGRGNILETKRVDLAEPQTTVN<br>VTIKPTFLTTPYGRVYFYYVDETGEFRYTEETFSVEVELQNQIEIKAPAEVKPGADVALEIKTSPKSFVGLLAVDQSVLLLGSN<br>NDLNKESFNWRLNGYDTSTPWQGGYSYYPGERTGVVTMTNAYFFYNRTAPDYNIQGFGGSSFAMRKTTVAHDSHVFHSGA<br>GGPTQAVGFSAESASASAAPVVRKNFAETWIFADIESTEEEVFKWVKTIPDTITNWVVTGFSLHPQKGLGVTNDQTNIKTFQP<br>FFVSVRLPYSVKRGEVINVPALVFNYLPKTLDVELTLDNEDQEYDFVDASNEVIGDQKRTQNIRVGANEAAGASFLIRPKVIG<br>NILLKFKAISPLAGDAIHKPLKVVPEGITQYQNRAFFINLKDTGEFKNTFELEVPEDVVPDSERVEFGLVGDLLGPVVKNLENL

	LRLPSGCGEQTMSKLVPNYLVRDYLKSIKKLTPALDTRIKRNLQDGYQHMLHYRHDDGSFSSFGPTKWRQEDPVRNGSTWL<br>TAYVLRSFSKIKDIIDLDEHILAKGYEFLLTRQAENGSFTEHGEYFYSSQRSLLTLTANSLLALLEEEKPNQAAIDKAVAYLSA<br>NTAESIELLPKSIAIYALQKAKAPEAAKQVASLKSLAKHEDDRTWWTEDLDKLRASKNCGRWWCWIWSQDVEITSYALLSL<br>LDSDQETADSVLNTVRWLIAQRNGFGGFASSQDTVVGLTALIKFAEKSGYEAAKWEVTVSNKGKREKTEKLNTSEENDLLL<br>QTVEFPQGTKSLEFEAKGTGAAMVQISYQYNLVEKEPKPSFKIQTTVLPESSPANLELSVCVDYVEEGESKESNMAILEVSLPS<br>GYTADEDSFADIRNIERVRLVETKNGDSVVVIYFENLAKNEEKCIRIEAYRTHAVANQKPSSVVLYDYYDTNKKATECYSIKS<br>KLCDICEGDDCKSKC
**HaTEP**	MLLKLWAFIALIALVASQESESSVYTVIAPSKLRPNIPFHVSASAHNVQKPLDMKIAIEGPADSGQYNNVEKRITLNSGETQIL<br>NFEIGEWSSGNYSLIVTGEGGMTFRNQTILTYEHKSYSVFIQTDKAIYKPGQLVQFRVLVVTPHLLPSVTGAINIFITDASGNRI<br>KQWNRLFTTKGISSGELLLSEQPVLGDWTINVDVLGQMFKKSFTVAEYVLPNFEVQVSLPPYITYSKPEFVATVNAKYTYGK<br>NVKGRVKLIVKPSLRYGYLANENKPVVTEAEIDGSIDIPVNVVRDLGLKEDTLTLEIDVIAEVEEYLTKRKYNASSVIKVYDK<br>EIKIDVVKSAESFKPGLKYTAHLKVCYQDDTPVTVTNGDQIVLKYGYSYDERNWDSRRIAVPNNGLLSVDFFPPLNPNITSFG<br>MSAEFRGYQYHLGNIEAAMSPSNSFIQVSLRTDNPTVDKEVELEINATEPLNQLVYEVLGRGDIVLAGAIDIPNVKSYRFSLPV<br>SYKMAPKARVVVFYVRKENNEIVADAINFDVAGVFRTPVSVKADVKDTKPGGLVNVSVETKPNAVVGLLGIDQSVLLLKSG<br>NDITQNDVITELETYDGGKKKKYWPYYRRKRSLWWPGSATAHDVFDDSGVVVLSNGLLYRFMPMIMYRSFRPEEDILEEHQ<br>QLDGDSMAYNSGSYGGKPRVRKHFPETWIWDLSPAGPDGKMLLSKKAPDTITSWIISAFAVDPVTGLGIAPDTTKVTVFRPFF<br>VKLNLPYAIIRSESLYVEVIVFNYMKKTMKADVTLENRKGEFEFASTSNEVSVPKEMTKTVDVPAQDGVAVKFLITPKKLGYI<br>DLKVTAQAENVGDAIVKKLLVKPEGSPQFFNKALLVDLRDPSSKKIDQTVKINVPGDAVPGSTSVMLSAIGDLLGPTVNNLD<br>KLLRMPQGCGEQNMLNFVPNIVISKYLKRVNRLTPAIEKRSLHFMESGYQRELTYRRNDGSFSAFGNSDKNGSTWLTAFVVR<br>SFHQAKEFMEIDEDIMSNSLEWLAKQQRADGSFGEPGEVHHKAMQGGSGSKSALTAYVLLAFLENQAQKTFGQEMDKASK<br>YLSKELKNSKDPYFVSIVTYAFHLSEHSEKDLALQKLLSLSTRGVDTIHWQRKKDNSVDSYYTPQSQDVEMTAYALLTYSLR<br>GDVAGSLPILRWLISRQNENGGYSSTQDTVVGIQALASLAFRLASTSISLNVSYSTVDTSNVLAINSENAMILQKVMLPPDTRS<br>VKVQATGFGVGIIQVTWQYNIESTGKVPSFALKPVLGKASTDDYIELDICTKYTQEGASNMAVMEVGLPSGFQADSETFPAIK<br>KLDKIKRIETQNGDTNVVIYFDRIDGNEMCVNVPAFRNHKVANQKPVPVKVYDYYDLAKSARMFYEPRVVDVCSLCESTDC<br>PDTCNKVPGVSSSSIAMYSVALITFATIAVSFFKSIH
**E TEP1**	MNTPNGLLAVLLLGICAATSHAERTYTVTAPKNVRVGTPYQVVVSIHNSPEDVEIFANLSCSSDDNSKPQLVTGSITAPNEITK<br>LLTLPIPDWWKPGNCELTVTGDKGIVFKRSASLGFNSKTSSVFIQTDKAIYQPGQLVQFRVLVVDPNLKPYASDELTVFITDA<br>QGNRIKQWNNATLKSGIFSGELQLSDQPVLGDWSINAQLNDARANKQVSVAEYVLPKFEVTVRPPTLAIFNDTELIVGVEAK<br>YTYGKPVKGKLVLNTTESYCRSPYSNYCGSTPVVVRTNIDGIANVKIPLSQFNFPDYYRNSLANLDFLAVVTEDLTGRMMNA<br>SAAGNIYSKREKIDVVQSSNSFKPGLPHTYKIKLQLQDGTPVTKADSLLTVKTSSSHGKPEVVTNYTVPASGIVSVEAFPDEDA<br>EFLRLNADYKDVSGSAYANKAQSISSKYLQLSLHNENEIEPKVGDVVQLDVNGTFYISRLDYEVVARGKIITSGSLKFDKDAK<br>SHSFPLNITQDMAPRARVVAYYVSSCGEVVADSLDFTVNGVFQTPVGLHTSENKTKPGAPIEVTVDTLPNSTVGLLAIDQSVL<br>LLKSGNDLNRNEIINDLGDYESGWRPSAFDRKKRSIWRPPGDTVTQLFDTAGLVFFSNGLFQKQPDFNYGPYPVRFNSFGGG<br>AGASFAESDSVQFQASTAPASFSGAGANNDKPKLRQSFPETWIWTMPIAGPDGKAVLKTTVPDTITSWQLSAFAMDDENGLG<br>MADGPSKVEVFRPFFVTLNLPYSVVRGESVAVQALVFNYMKEDVEAEVTLENLNDQFELTGLLNRVDVDHNATSEKKTVK<br>VKAGDGSSVSFLITPKVVGPIDLTVSAVSSKAGDALNKKLLVKAEGSPQYFNKAVLVDLRNTSNFKSQVEVNIPPFAVKDSEH<br>VEVSAIGDIMGPTVNNLDKLIKMPYGCGEQNMINFVPNIAVSDYLNTTNQFSDKLRTKAIKFMEAGYQRELTYKRPDGSFSA<br>FGTSDKNGSTWLTAFVVRSFKQAKPYISVDDNVIDASLRYLKSTQKENGSFVENGEVHNKRLQGGAAGGLSLTAYVTLAFL<br>ENSDKTEYSNVTKRAVKFLEDEVDTVEDPYELAIVSYALHKAGSPAKDAAFQRFLKKAEKKGDNVFWSKPLEKPPTNSSYF<br>YYPPPSVDIEMTAYALLTHLERNLITEAVPIMKWLITQRNENGGFSSTQDTVVGIQSLAGIASHITSPDGAKMELDFEYDGRHK<br>KLTLDKGNAMVLQREELPSETREVNVGAEGKGFGIVQVTWSYNLDNKTQSPVFEISPKVKQIDKDSFDLTVCAIYEDVDKSS<br>NMAVIEMNLPSGYVVEGESLPQVGQHDRLKRVETVDGGTKVQLYYDEMNDVEVCPHITAYRTFPVANVKPAAVSVYDYY<br>DNDQRAETFYNAPSSDLCDICTGSECSSKCKKS
**LlCD109-1**	MKLLLLAALVGAAFANDSYVVVAPSKVRPGMEFSVSVNILKATGDVTVTASMVRTNNQSTVSTNVATFQEGAPGTIDLQMP<br>KVLQSGTYELQVSGAGGLTFSNKTHVTFSAKSKSVLIQTDKAMYKPGQTVNFRVLAMYPDLSVYKGNLDIEIYDSKSNKIKQ<br>WKNVSAAEGVVTKDISLSSQPVLGDWKIKVTAGNSAEEKAFTIAEYVLPKFEVTVELPSYVLESATSVPVTVKAKYTYGKPV<br>AGPVVVESKLVQSYYHSTTPSVQRTGTLDNQGRAVIIFPLTELRQLRVSATSSLSNQKIKFSANVTESLTGITLNGSSIVQVYN<br>KAVKLEFPSSNPKNFKPGLHYNGYLQVSQPDGLPVASAVGQSVTIKTTVTAELPAPTTTHYYYSPRTMSFHLADQRLTLNDN<br>GLIPLQLSIPSNATNINVHAEFQGASAYSSITKSYSPSNSYIQLFLRSTNQLTAGGMVNFEFKATEPVQTLVYQVMSRGSIVSAG<br>KVAAAGASSKLFSLSVTPAMAPNARIVLYYVRTDGEIVTDSISFDVEGAFQNQVSVSIDKTQAEPADAVRVTVNADPQSKAY<br>LLAVDQSVLLLKSGNDITAGQVIDELKGYDTTQTKSNIVPFGGGFGGIRPLGLARAKRMIWWPYPVYYGGSDANQIFKNAGV<br>VVMTDALVYKYVKPYRPRHFRRMMAFSRPNLGLRMSFAMASPMMGSIATDSSRLRVADSNAQPLKEVEAVRMLFPETWL<br>WSNVSVGADGTATLTTTVPDTITSWVTSAFAINPTTGLGVAPTAAKLRVFRPFFVTLNLPYSVVRGEQVVIQAIVFNYLPTDQ<br>DVRVSMAQSQDFFNLVHDANGQEDLRHEDQVQNVRVPAGEGKSVFFPIVPARLGNVEAVVSAQSTQAADAVRRQLLVEAE<br>GVPKEFGVSVLVDLKNTTTFTKTVPISLPPNVVVDSSRARVTAIGDLMGPTVNGLDSLLKMPTGCGEQNMLGMAPDVFVSA<br>YLKATNQLSGDIAEKAIRFMEHGYQRELTYQHKDGSFSAFGDRDPSGSMWLTAFVAKTFHQAKPYIFIDDDVISRSIDWMLA<br>KQKPDGSFPEPGRVIHKNMQGGASNGTGLTAFVLIALLENNDLQGSISQRVSAATTEAIKYLMGKLPTVTDDYILAMTTYAL<br>TLAGDTGAASSFSTLVSHAVVKDGLRYWHKVTTKPTSTSRYWHSPAAVSSDVEMTSYALLTYAAKNDFIGGLDVMKWVAS<br>QRNPHGGYASTQDTILALQALSEFARLAYSKSFNIEVSVTAASFAKQFSVNQNNALVLQSADLPSVPSSVTVSATGSGIALVEI<br>GVFFNVEAEVEEPTFEVKVNLVKDTLNLLEIETCAKWLSSDSSGMAVQEVGIPSGFEVDLESIKQPDVLKRTELQDRKVILYF<br>DEIGTIPVCTTMTAQRTGLVAKSKPVGVRVYDYYEPENQATTFYESTSLKNSSVCDVCADCDCPAVKGR
**LlCD109-2**	MWIIHTLIGAWLLSLATSQDSNVAQSCLLDNTNGCALARPPQYMVLVPRRVRPNQVIQAFVSILRMEYDSEFVNVRISIVRDN<br>VEYTGASLRFDRASSRLMQLQMPSNAQFGNYRLRIEGTLDKENTGNIFQNETDIEFSAKQASLFIQMSQPIYRQEQTVNFRVV<br>PIQPNMMPNYGSMTIYVEDPTGIPVRRWLGLQTNAGGFVSQKFELADQPNFGTWYIRVDAFGHTYRQPFQVEDFWEPRFDV<br>NVTVPPYLLENSTTIIRGVMLANHTSGRPCKGNATITVFFRPREEVWNQTKGWERPFYNGMMPNRNPNLGPPEYKPIADIPV<br>MDYYMYLQYDYWFVDYYDGRIDFEYPKEKLFDLARRSGEYGQLVDSSVYFWLNVTDWYSGMNRTGWAGTIMASSEVQL<br>QWVGEPVRTFKPRSTMKVQIAVSMADGTPVRDSTKFVTLSERIAGTTTSGTQGNPQTRTPVNGIVEFEIYLPSNAQTMTLTAT<br>YNNDYLRTKQIEMKASRYYSPSNSYITITTSTDHAKVNEYMIFHVKTSHFIPRIYYQVVAQGNIIIGDELEMTSKQKTFAVALS<br>REMVPIARIVVYYLRQPEEIVTDVLNFFVNGTRQNQVKLGINRGKDFSRDTVEFNAEADPGSYVAFSGMLLDLYSRGLNDGI<br>TENSLIDELATYDEPANGTFRHLWRISDTDYEYKFFHGSDYGVDANTSFSSAGLLILTDAELTRVRNAELCDPRLDQYPCLTG<br>EGQCYTKEQRCNGKFDACTDGGDEYGCGVEETGLKHASAMDRVSRVMRFYDNSSWAWQEIFVKPDGRVDFRVNVPKYPL<br>SWVINGLSISRDLGLGIMTRPIRYDAARYMYIQVEHPEIIARGEQVGVRVTVFNYWYDDDYLEVLITMHGSENYEFVTVEELG<br>WVYSYRPNTHKGDHQTIVFLEPGESKDIYMPIVPSQDLIQGMLTFHVSATCFMERDEYTGNMTVIPDGVINYYHTPYLIDLIRF<br>ASIQVPDFDVPVPEQFVVPEVREHLYVPGSAKSVPSLFGDVVTPGFFEEYLNAENVMHRPYGGGEQITFNFAYNLMTLKFMK<br>ASQQLSDEQLLNTLRKCNIAMQRVLSYMNGSEGSFHMFRDDPNPSVWLTAFVAKTMHEARFGEWERDLFIPLELINRMVIY<br>MCSQQNKTTGAWDPDPNRPTYDRKMASYEAMKSEQMHANRIPLTAYVLIALYETKDVSGEALGCLDTARASAANFLASQV<br>DDIPKEEVFHLSITAYALSLSQEKSSKAFDRLWGWVRNDSEGLYFADRKVPENPSEILNNVRFLHPRQELMNDGYATQATAY<br>ALMALINNNKPKVYRDLLMEWLNSMRNTIGGFASTQDTLVAMDALFKFTQVDPNRNVFDLMVRLESTATPTWYRERYLTK<br>TDYTKLYLDSIPIVWGMVKVLAQGTGRALMQLTTTVNVEYPHLLKPTEREDMNDRDSDPIQFFDLMIDRIQWHGRNFSIME<br>MYPCARWIYTERSLTSGMAVMEIDIPSGYVVMNDTLRDYVRSGLVPNLKRAEFYGRKVVFYFSYLDQSYSCVMFRAERWY<br>PVANATIQHRMRVYDYYEPGMHNTTMYTTYNLFTLNICYVCGSYQCPYCPYFNVATAIKATITLLGLISGFLIQRFYILRS

**MyCD109**	MLWAGLITLLLGLAAAKDTYVVITPKDVRPGVPLNISVNILHATGDVHVTAKLIHVTDKSVKASSSATFQQHVPDTMRIMVP<br>DTIPSGTYQLTVEGSNGLTFTDKTNLHYASKGMSIFIQTDKAMYKPGQTVNFRTFAIYPNLTVYSGPMDIEIYDPNSNKIKQW<br>FGLKDASGVLTNFMAMDTKPVLGDWKIRVKTHGLTKDKMFTVAHYVLPKFEVTVDLPSYDLTSATDIKGTVKAKYTYGKP<br>VNGTVKIRAHVDFYQSQYYHPEPIPTIELTMDINGEAKFTLPVSGLTSHTYYTSLNGHNVVVEANVTESLTQITLDGTSKMHF<br>YTHAEKIELLPSNPTTFKPGLQYIAYAKVVQQDDMPLAAGSSKSLIVHTSVTANLPETTTPMYYYGPRTMNYQLPDQSFTLTD<br>TGLVQAKIDIPHNATSISLNFKYGQITQYKSVQRSYSPSDSFMQIFLESNNLQAGHDKVVDFRVVSTSPIDKLIYQVLGRGSIAV<br>SGSINGNNAKAFQFHVPLNAKMAPNARIVAYYVRADGEIVTDSISFDVSGTFENEVSIRFDKTKAQPGEGINVDVTADPNSIV<br>NLLAVDQSVLLLKSGNDITPAEVVDELKSYDTIVHSNNGGPIFLGGGGFGGGGIMPEPMPVGRKKRMIWWPFTTYYGGSDAE<br>QIFQNAGVNVMTDALVYHHVEPHIYYPQMFPMAAAGMAPGAAMGGSMGAGMATGLATGGMGGNPVLKEVETIRSDFPET<br>WLWLNKTIGPDGHVTIAATIPDTITSWVASAFAVHATSGLGIAPTSAKVEAFRPFFVSLTLPYSVVRGEQLVLQANVFNYMT<br>MDMDVVVTLEKNDDLVNVVFNTHGTESYIAQTTTKTVHVTAGGTKSVFFPVVPAGLGSVSINVKAQSTLAADAVRRQLLIE<br>AEGVPKEYNIPMLVDLKHNTNFAETVDVTLPAGVVAGSQRVRVSAIGDLMGPTVNGLDKLLRMPTGCGEQTMLGFAPDVF<br>VTNYLTDTHQLTSSVEEKAINFMEKGYQRELTFQHTDGSFSAFGDRDPSGSMWLTAFVAKSFHQAKRHVFIDDETLTRAIDW<br>MINRQAANGSFPEPGRIIHKNMQGGSASGASLTAFVLIALLENSDLQGGVHLRIQSAANKAQTYLEGEVSAMTDPYGLSICSY<br>ALTLARSHSSATTFHKLMATAVTKDGMTHWHEPESASSSTGHYWSPPHQQSKPVDIEMTSYGLMVFAHNGQFTEGLPFMK<br>WITKQRNPNGGFSSTQDTVLALQSLSEFARIGYSEHFNMQIGITAGQTTHKFNVTRQNALVLQSLELPSIPSHVTVTGTGSGM<br>GLVEVSVFFNVEQEVEQPSFEVDVTIIEETINSLKVRSCTKWLKTGASGMAVQEVGVPTGFAPDVESIGKIATLKKTETENRK<br>VILYFDEITTTPLCVTMDAFRTDKVAKSQPAPIRVYDYYEPRTTLD
**SmCD109**	MLVVVWALIALFAYSYGQESTTVASTSSAVYTVIAPVKLRPNIPFHVSASAHNINSPLEMKIEIEGPADNGQYNNIAKQVTLNS<br>GETQILNFEIGEWSPGNYSLTVIGEGGMSFRNQTLLTYEHKSYSVFIQTDKAIYKPGQLVQFRVLVVNPHLLPTVTGAINMFIT<br>DASGNRIKQWNRQFTSKGAVSADLLLSDQPVLGDWSINVDILGQMFKKTFTVAEYVLPNFEVQLSLPTYVTYSKPDFVATVK<br>AKYTYGKPVKGHAKLLVKPSLRYGYLANENKPAVTEAEIDGSVNIPMNLVRDLGLKEDALTLEIDVVAEVEEYLTKRRYNA<br>TSVIKMYDRDIKVELVKSSESFKPGLKYTAHLKVCYQDDTPVVVTNGDQIVLKYGYTYDEREWDSRSIAVPSNGLLSVDFYP<br>PVNSNVSLLSMNAEFRGLEYHLDNVEAAMSPSNSFIQILLRTENPTVDKEVELEVNATEPLSQLVYEVLGRGDIVLAGAINIPN<br>VKTYRFSIPVTHKMAPKARVVVYYVKAENNEIVADAVNFDVAGVFRTPVSVSTDVKETKPGALVNVTVETRPNALVGLLGI<br>DQSVLLLKSGNDITQNDVIAELETYDGGKKKYRYNPFYRKKRSLWWPGSATAHEVFDDSGVVVLTNGLVYRYIQLIMYRSY<br>HPLAESLDDELNAVDSMMYNHGNGYGSKPRVRKNFPETWIWDLGTAGNDGKMSLAKNIPDTITSWIISAFAMDSVNGLGIA<br>TENAKVTVFRPFFVKLNLPYSVIRSESLYVEVIVFNYKKKPTKADVTLENSNGDFEFTTAGNEITTPNDQMTKTVEVPAEDGIS<br>VLFLITPKTLGYIDLKVTAVSENAGDSVVRKLLVKPEGAPQFFNKAILMDLRNPTANVVKESVAIPVPEEAVPGSTSVQLSAIG<br>DLLGPTVNNLDKLLRMPQGCGEQNMLNFVPNIVITEYLQRVNRLTPVIQKKSLNFMQSGYQRELTYRRNDGSFSAFGNNDK<br>NGSTWLTAFVVRSFHQAKEFIDVDENVMTSALEWLSRRQKPDGSFDEPGEVHHKAMQGGSGAKNALTAYVLLAFLENQAQ<br>KTFGQEMDKATKFLLRELKESQDPYFVSIVTYAFHLAEHPEKDAALQKLLSLSTRGVETIHWKRPQEETVSYYRPQSQDVEM<br>TAYALLTYSVRGDVAGALPILRWLISQQNENGGYSSTQDTVVGIQALASLGFRLASTSISINATYSFGNMEKSLNINSENAMV<br>LQNVILPSSTRAVNLRAEGFGVGIVQVTWSYNVKSTVKTPAFNLKPVLGKASTDDYMELDICTSYKEKGASNMAVMEVSLP<br>SGFQADSETFPMIKKLEKIKRVETQNGDTNVVIYFDRIDGEEMCVNVPAFRNHKVANQKPVPVQVYDYYDLSKSARMFYEP<br>KVVSLCSVCEGKDCGSGCNDVADITNSSPSFAYSAPLIAVASFIILCFMRLVR
**EsCD109**	MNSLTRFCWLLAIVVATCYGEKIETIRPPVEQGGKYLVYLVDQVSSEETLNISVSIFNTTGDVHVKVALRETRNKTVVATIED<br>NISSESTKMLAMPLPFLLNNNYLVEVNGTGALNFSDNAYIYVSHRKSDSKKHSIIIQTDKPLYKPSDSVKFRVFGIQPNLLASK<br>APMNISIINPKRTIMAHWTNMENKTGVISLEYELSDKPVFGTWTIEMDQENKKERHQFKVREYVLPNYKVTVESEKTLASLG<br>HGYLFIYEDVIKVNVTAKYTFGKSVRGDLVLNATIGDSDDALRLTFEQKIEGTNHIEFKMNDFIKTNMVKVSRYQSFCTPRKII<br>FKAMVTEALTGKKMYSEPFTVEIVNSPIKLEIDTPIIYKSGLPSQVHMSVSRLDDKPLTEEDRKFPVSVMIKYGNGYTHRDEY<br>DIPQNGSIKFKLLLPDYKEGPVTIEAKYTGHRGSVKYETTDTAYPRFFMNNHHDNVEATLLQERVILGDDIKVFMNSTAPISW<br>ALITIISNKKMLSNELRNMEDKTHFVLEIPTSEIMIPEASVIISCLTSNGKFMVDASIAKITIAFKNDVVGTFTEKAGKPGTNVDF<br>KVKTMPGSAVFVMAVDKNLHLLESAKYITEDMIYDALLGSENSNRWRYSVTDINGVINSLGMEIMTNFIKKHRSISYNTLGR<br>TAYHMESAPMGPGPIPVSSYYSPVSSSVSARIRKYFPETWIWLSGDASDAGEFSFNNVVPDSITKWTTNAFAFHPEKGLGIMK<br>ESAEFLSYQDFFIVPVLPPFIIRGESFVLKVSIFSNLTQDQEVTVTLPESDQFTLKDSENAKTVTVNAHSDNTVQFEVVPTAVGE<br>IPVKVQASCEVAADEVHKKITVKPEGYTLYFNKAFLLKIEDGVPAEIDYEFEAKPEFVKDSERVEVSIIGDIMGPSFENLENLIR<br>IPSGCGEQNMINFAPNVYAKNYLSARAKLTDELAKKILYYTRRGYKNQMQYQHSDGSFSAFGHRDKVGSTWLTAFVLRCFG<br>QAHKSMPDLYIDEGVFRQGITWLLRQQNKQGYFVEYGRVIHSAMQGGTNNGLTLTIYTLISLVENKHIMPNVTDSVIRDVTA<br>NLPIMRNDTYSLGMMLYLMTLVDDTDRFEEIQTILEDKAINKDDMKYWDTEVDTESDPYSHSVSTRVELASYILMAYVNKE<br>NIEEGLPIMKYILSQRNSNGGFQSTQDTILGIEALGKFAQNIKSPTTFTVTVTSDEKDSYTFPEINSEMSTVLYTHVLPSSTKTIHI<br>KGEGTTSEDATATYAFAQISWQYNIKLGSADKVFEGKVEYRTLSSNEMALNICHKMKKMNITGMVIIEAALPSGYMLSNKK<br>ELLASENISKVEIEETEVVLYLNKLDTSFSCHELEATKIYEVKGHTSPLPVKIRLYYQPDLELVTMYTITGGSE
**HsCD109**	MQGPPLLTAAHLLCVCTAALAVAPGPRFLVTAPGIIRPGGNVTIGVELLEHCPSQVTVKAELLKTASNLTVSVLEAEGVFEKG<br>SFKTLTLPSLPLNSADEIYELRVTGRTQDEILFSNSTRLSFETKRISVFIQTDKALYKPKQEVKFRIVTLFSDFKPYKTSLNILIKD<br>PKSNLIQQWLSQQSDLGVISKTFQLSSHPILGDWSIQVQVNDQTYYQSFQVSEYVLPKFEVTLQTPLYCSMNSKHLNGTITAK<br>YTYGKPVKGDVTLTFLPLSFWGKKKNITKTFKINGSANFSFNDEEMKNVMDSSNGLSEYLDLSSPGPVEILTTVTESVTGISRN<br>VSTNVFFKQHDYIIEFFDYTTVLKPSLNFTATVKVTRADGNQLTLEERRNNVVITVTQRNYTEYWSGSNSGNQKMEAVQKIN<br>YTVPQSGTFKIEFPILEDSSELQLKAYFLGSKSSMAVHSLFKSPSKTYIQLKTRDENIKVGSPFELVVSGNKRLKELSYMVVSR<br>GQLVAVGKQNSTMFSLTPENSWTPKACVIVYYIEDDGEIISDVLKIPVQLVFKNKIKLYWSKVKAEPSEKVSLRISVTQPDSIV<br>GIVAVDKSVNLMNASNDITMENVVHELELYNTGYYLGMFMNSFAVFQECGLWVLTDANLTKDYIDGVYDNAEYAERFME<br>ENEGHIVDIHDFSLGSSPHVRKHFPETWIWLDTNMGYRIYQEFEVTVPDSITSWVATGFVISEDLGLGLTTTPVELQAFQPFFIF<br>LNLPYSVIRGEEFALEITIFNYLKDATEVKVIIEKSDKFDILMTSNEINATGHQQTLLVPSEDGATVLFPIRPTHLGEIPITVTALS<br>PTASDAVTQMILVKAEGIEKSYSQSILLDLTDNRLQSTLKTLSFSFPPNTVTGSERVQITAIGDVLGPSINGLASLIRMPYGCGE<br>QNMINFAPNIYILDYLTKKKQLTDNLKEKALSFMRQGYQRELLYQREDGSFSAFGNYDPSGSTWLSAFVLRCFLEADPYIDID<br>QNVLHRTYTWLKGHQKSNGEFWDPGRVIHSELQGGNKSPVTLTAYIVTSLLGYRKYQPNIDVQESIHFLESEFSRGISDNYTL<br>ALITYALSSVGSPKAKEALNMLTWRAEQEGGMQFWVSSESKLSDSWQPRSLDIEVAAYALLSHFLQFQTSEGIPIMRWLSRQ<br>RNSLGGFASTQDTTVALKALSEFAALMNTERTNIQVTVTGPSSPSPVKFLIDTHNRLLLQTAELAVVQPTAVNISANGFGFAIC<br>QLNVVYNVKASGSSRRRRSIQNQEAFDLDVAVKENKDDLNHVDLNVCTSFSGPGRSGMALMEVNLLSGFMVPSEAISLSET<br>VKKVEYDHGKLNLYLDSVNETQFCVNIPAVRNFKVSNTQDASVSIVDYYEPRRQAVRSYNSEVKLSSCDLCSDVQGCRPCE<br>DGASGSHHHSSVIFIFCFKLLYFMELWL
**SpC3**	MGSIILIFVLAISASLVPGGVTQDPPATFFVISPNVFRAGVEEKVVVTLIRSPPNVQNIEVKVSLMRPGSTVTFSEDTRLVSPGSS<br>QSFSVVIQAGDLMRNEGAFQHMVLKAESLNPIYPFEEQTDILVTLQSGYVFVQTDKPIYTPNQDVMIKVMSLDQDMLPSDRE<br>LHVEIMDPSGTSVKRLRETHADVPASIGRLQVHNHPASVNGPFRLSMDPSSLSSQVTFVVKEYVLPTFSVSIETPKYILAGADS<br>VVSTIIAEYVFGKPVIGRYTFKYGVVDNGTVQELGTKQGLLGDSGRAEVTLDDLSTDFGEDWFVRFRGRHFHANAIVHESAT<br>QFSEASINTKAIFVDSPYKFSTKRTVQHFKAGLNLQVKLDLTFANGDVAPNVPVNVIATATLRDGSEELLRRPDIAGGQEEISN<br>TDDQGGVSMVLNVQSSITSIHIVAATHDPQYPNNQADISFEVSPGVSANGHDYLVIRPQNTDDRNLNVDVSTDFLIQRIGTTG<br>DQDIDLHFLCITGGKVVLEGVQRSISAAGTNLAITIRAYMAPQMRLIVYYVTMDGSVIADSLLLGVEEKCRQDPQLSLDILPR<br>NVGPERDVYEPNGQIQVEVTAPIDSNVGLLAVDKAVYLLRDKDRMGKQKMYERMRSYDTGCGPGGGQNTAQIFKDCGMT<br>VLTNAGLDVPIREDVECMDEDTRRKRSIDRDQLCLYDPTYLADCLADKPRLRRVLTEGGWLCPRRARVLEAECNLEPDQTQ<br>VYRDCCINSMNPVVTATSRSGGDGGEQNAAVKVRDDFRETWFFDVVSMPEDGSPYLYPVSIPSSITDWHLTAVSLSPTQGMC<br>VEDETTVSVFQDFFIQLHLPYSVVRLEQTQVIATIFNYGFSDFEVSVNFTVDQGLCTAENPAIRHVFVESKRAASTTFHCTPCG<br>SRGIPYTVTAGGSKKRDSVRNNLRVVSQGVMQRKSRSLTLNPGRVMFSDDVTTPSPNNSLGSGPGTIFGEGFQHEEIAISLPGS<br>SIPDTESCSVKLIGNLLGTASTDPIGGLDHLVRQPRGCGEQTMIYLAPTLFVYQYLIAVGSDTAEQEARIYDYIADGVARELTY<br>RQDNGAYAAWKHRPGSTWLTAFVVKVFSQANRFTRVEPGHVEGSINWLIDNNQLPSGAFQESQQVIHQEMIGAVKGETSMT<br>AFVLISLLESRNLLVPANQKIDEAIGKATEYLVTQVENIDRVYDKALVTSALRESASVGTANGKLWEDRNEDGTGAVSFTPD

	DANYEDGSQPFWLQRKPSAIEIETSGYALLAQLALLDYQKAGKIALWLSKQQNDGGGFVSPQDTVVALQALAKYTERPEFN<br>TIEMNCDVATEQIPLHRYHIGNDNAKVQEEVDVSPSIGRSLTFDSRGTGVAKANVELRYNTEKSDIDTCPFHLNISAVEVPSDE<br>VSGQNVKGLMITVCTSYNGDGTTHMIIVDVGLYSGFKAVEEGLTGLKQVVNGSSLISSYEASSRSVIFYLDTIPSDEDLCFTFS<br>AESDVVVGNVQAAAVHVYDYYDPEKSCTIFYKPGDGSALLSTLCSENECICSGGSLEYCNIDPCPGPYTRIDLEGTACASHSS<br>YALKIRIDEVEIKEGFRICKFTVLNPIKTGDEDVPHQAQRQLFINEGCDCPKVKGRNIGGTFLLVGQKSLKYTTEQGEERYRYV<br>YGPTSKLEFWPATRKARNAAVFDKLVAFEAQMAPASACSNEN
**MmCD109**	MRSRRLLSAAHLLCLCAVALAAPGSRFLVTAPGIIRPGANVTIGVDLLENSPPQVLVKAQVFKIASNKSRSILEAEGVFHRGHF<br>KTLVLPALPLSSADKIYELHINGQSENEIVFSNRTRLTFESKSISVLIQTDKAFYKPKQEVKFRVLTLCSDLKPYRTSVDIFIKDP<br>KSNVIQQWFSQKGDLGVVSKTFQLSSNPIFGDWSIQVQVNDQQYYQSFQVLEYVLPKFEVTVQTPLYCSLKSKQLNGSVIAK<br>YTYGKPVKGSLSLTFLPLSFWGKKKNITKSFEINGFANFSFDNYEMKKVMNLKPLTDVSEGSYENVDPSFPGPAEIIATVTESL<br>TGISRMASTNVFFKQHDYIIEIFDYTTVLKPSLNFTATVKVSRSDGNQLTPEEIENDLVTVVTQRKNNHPESQRDQEMDYIQTV<br>NYTIPQNGIIKIEFPVMSISGELQLKAYFLDGTSSVTVHSMFTSPSKTYIQLKTRDEYIKVGSPFDLMVSGNRQFKDLSYMVISK<br>GQLVAAGKQSSRTFSLTPEASWAPKACIIAYYIAEDGEIINDILKIPVQLVFENKVKLFWSKPTVKPSDKVSLRISATQSDSLVG<br>IVAVDKSVTLMENSNSITMETMVHELELYNTEYYLGMFMNSFAVFQECGLWVLTDATLIRDSIDEVYDTEEYSERFAEENEA<br>NLVDFEDASSVNNVHVRKNFPETWIWLDAYMGSKIYEEFEVTVPDSITSWVASAFVISEDLGFGLTTVPAELQAFQPFFLFLN<br>LPYSVIRGEEFALEVSIVNYLKDTIKVVILIEESDSFDILMTSNDTNGTIYRKTVQVPRDNGVTLVFPIKPTHLGEIPITVTAASPT<br>ASDAVTQTIVVKPEGIEKSYSKSVLLDLTDSNVESKQQSMRFSFPPDTVIGSERVQITAIGDILGSSINGLSSLIRMPYGCGEQN<br>MIYFAPNIYILDYLTKQKQLTVNLKEKALSYMRQGYQRELLYQREDGSFSAFGDIDSSGSTWLSAFVLRCFLEADYYIDIDQD<br>VLHRTYTWLNAHKKFNGEFWEPGRVIHSELQGGTKSPVTLTAYIVTSVLGYKKYQPNIDVQDSIKFLEFEFSRGISDNYTLAII<br>SYALSTVGSPKAEEALNLLMQRSEKEGDTQFWLSSGPALSGSWQPRSVDIEIAAYALLAHTLHHVSEGIPVMRWLIQQRNSL<br>GGFVSTQDTVVALKALSEFSALVHKENTDIQLTVTGPGIPRSIHFRIDSQNLFLLHQEELHALDPITVNVSAHGSGFAICQLNV<br>DYNVKGSGSSKRRRSTENQEVFDLDVIVNNEDDISHLNLNVCTSHLGSERTGMVLMEVNLLSGFSASSDSIPLSETLKKVEYD<br>NGKLNLYLDSVNESQFCVNIPTVRDYKVSNIRDGSVSVMDYYEPRRQAVRSYNTQVKLSSCYLSPDTNCKSHTDGATDSLRR<br>SSSLLVFCSVLLYFVQHHsC3MGPTSGPSLLLLLLTHLPLALGSPMYSIITPNILRLESEETMVLEAHDAQGDVPVTVTVHDFPG<br>KKLVLSSEKTVLTPATNHMGNVTFTIPANREFKSEKGRNKFVTVQATFGTQVVEKVVLVSLQSGYLFIQTDKTIYTPGSTVLY<br>RIFTVNHKLLPVGRTVMVNIENPEGIPVKQDSLSSQNQLGVLPLSWDIPELVNMGQWKIRAYYENSPQQVFSTEFEVKEYVLP<br>SFEVIVEPTEKFYYIYNEKGLEVTITARFLYGKKVEGTAFVIFGIQDGEQRISLPESLKRIPIEDGSGEVVLSRKVLLDGVQNPRA<br>EDLVGKSLYVSATVILHSGSDMVQAERSGIPIVTSPYQIHFTKTPKYFKPGMPFDLMVFVTNPDGSPAYRVPVAVQGEDTVQS<br>LTQGDGVAKLSINTHPSQKPLSITVRTKKQELSEAEQATRTMQALPYSTVGNSNNYLHLSVLRTELRPGETLNVNFLLRMDR<br>AHEAKIRYYTYLIMNKGRLLKAGRQVREPGQDLVVLPLSITTDFIPSFRLVAYYTLIGASGQREVVADSVWVDVKDSCVGSL<br>VVKSGQSEDRQPVPGQQMTLKIEGDHGARVVLVAVDKGVFVLNKKNKLTQSKIWDVVEKADIGCTPGSGKDYAGVFSDAG<br>LTFTSSSGQQTAQRAELQCPQPAARRRRSVQLTEKRMDKVGKYPKELRKCCEDGMRENPMRFSCQRRTRFISLGEACKKVF<br>LDCCNYITELRRQHARASHLGLARSNLDEDIIAEENIVSRSEFPESWLWNVEDLKEPPKNGISTKLMNIFLKDSITTWEILAVS<br>MSDKKGICVADPFEVTVMQDFFIDLRLPYSVVRNEQVEIRAVLYNYRQNQELKVRVELLHNPAFCSLATTKRRHQQTVTIPP<br>KSSLSVPYVIVPLKTGLQEVEVKAAVYHHFISDGVRKSLKVVPEGIRMNKTVAVRTLDPERLGREGVQKEDIPPADLSDQVP<br>DTESETRILLQGTPVAQMTEDAVDAERLKHLIVTPSGCGEQNMIGMTPTVIAVHYLDETEQWEKFGLEKRQGALELIKKGYT<br>QQLAFRQPSSAFAAFVKRAPSTWLTAYVVKVFSLAVNLIAIDSQVLCGAVKWLILEKQKPDGVFQEDAPVIHQEMIGGLRNN<br>NEKDMALTAFVLISLQEAKDICEEQVNSLPGSITKAGDFLEANYMNLQRSYTVAIAGYALAQMGRLKGPLLNKFLTTAKDK<br>NRWEDPGKQLYNVEATSYALLALLQLKDFDFVPPVVRWLNEQRYYGGGYGSTQATFMVFQALAQYQKDAPDHQELNLDV<br>SLQLPSRSSKITHRIHWESASLLRSEETKENEGFTVTAEGKGQGTLSVVTMYHAKAKDQLTCNKFDLKVTIKPAPETEKRPQD<br>AKNTMILEICTRYRGDQDATMSILDISMMTGFAPDTDDLKQLANGVDRYISKYELDKAFSDRNTLIIYLDKVSHSEDDCLAFK<br>VHQYFNVELIQPGAVKVYAYYNLEESCTRFYHPEKEDGKLNKLCRDELCRCAEENCFIQKSDDKVTLEERLDKACEPGVDY<br>VYKTRLVKVQLSNDFDEYIMAIEQTIKSGSDEVQVGQQRTFISPIKCREALKLEEKKHYLMWGLSSDFWGEKPNLSYIIGKDT<br>WVEHWPEEDECQDEENQKQCQDLGAFTESMVVFGCPN
