# Supplementary figures and images for: Thioester-containing protein TEP15 promotes malaria parasite development in mosquitoes through negative regulation of melanization
Source: Parasit Vectors. 2025 Apr 1;18:124. doi: 10.1186/s13071-025-06772-5 (PMC11963550; doi:10.1186/s13071-025-06772-5)

**Additional file 3: Figure S1 Full Western Blot results.**

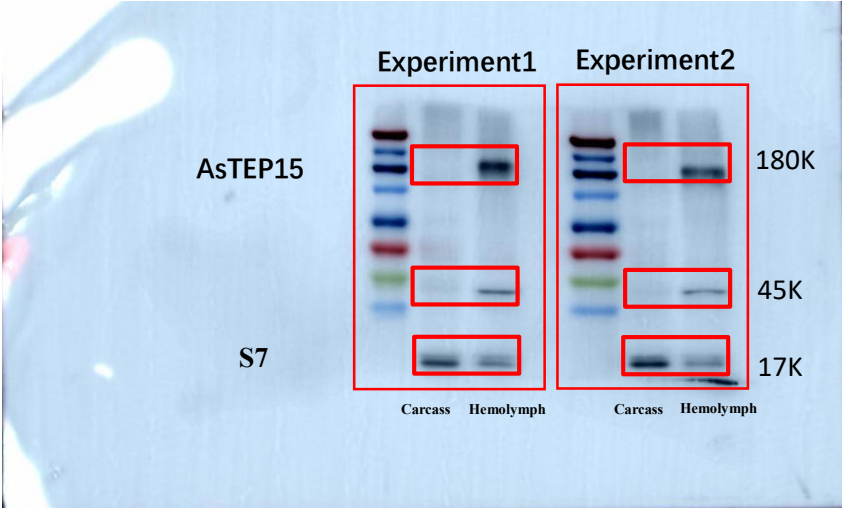

Supplement: Supplementary file 3 — Additional file 3: Fig. S1. Full Western blot results. [file 13071_2025_6772_MOESM3_ESM.pdf]
