# Supplementary material for: Thioester-containing protein TEP15 promotes malaria parasite development in mosquitoes through negative regulation of melanization
Source: Parasit Vectors. 2025 Apr 1;18:124. doi: 10.1186/s13071-025-06772-5 (PMC11963550; doi:10.1186/s13071-025-06772-5)
Supplement: Supplementary file 4 — Additional file 4: Fig. S2. The differential mRNA abundance of AsTEP15 in uninfected blood-fed versus sugar-fed mosquitoes. [file 13071_2025_6772_MOESM4_ESM.pdf]

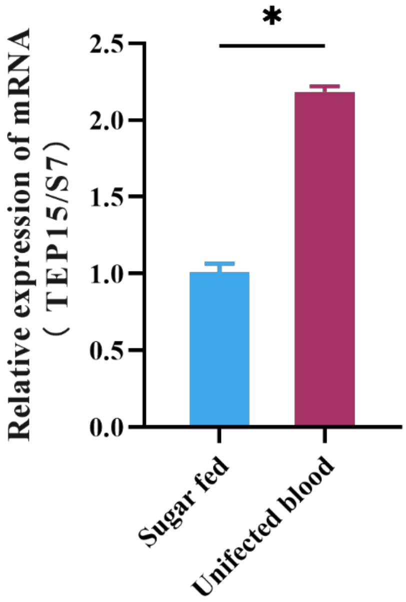

Additional file 4: Figure S2 The differential mRNA abundance of AsTEP15 in uninfected blood-fed versus sugar-fed mosquitoes. The mRNA expression of AsTEP15 in mosquitoes (n=15) fed with uninfected blood was measured using real-time PCR on 1 day after feeding. TEP15 levels were normalized to ribosomal protein S7 and expressed as fold induction compared to sugar-fed controls. Experiments were repeated three times, with error bars representing standard errors.
