# Supplementary material for: Thioester-containing protein TEP15 promotes malaria parasite development in mosquitoes through negative regulation of melanization
Source: Parasit Vectors. 2025 Apr 1;18:124. doi: 10.1186/s13071-025-06772-5 (PMC11963550; doi:10.1186/s13071-025-06772-5)
Supplement: Supplementary file 5 — Additional file 5: Fig. S3. Gene ontology (GO) analysis of differential gene expression in P. yoelii-infected mosquitoes after AsTEP15 knockdown on day 7 post-infection (PI). [file 13071_2025_6772_MOESM5_ESM.pdf]

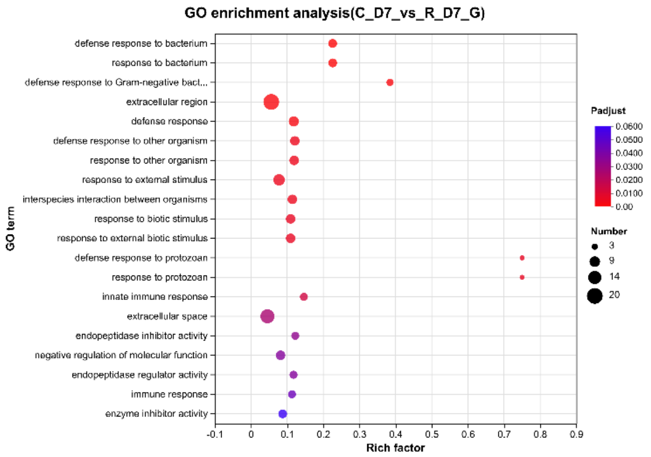

Additional file 5： Figure S3 Gene ontology (GO) analysis of differential gene expression in *P. yoelii*-infected mosquitoes after AsTEP15 knockdown on day 7 post infection (PI).
