# Supplementary material for: Thioester-containing protein TEP15 promotes malaria parasite development in mosquitoes through negative regulation of melanization
Source: Parasit Vectors. 2025 Apr 1;18:124. doi: 10.1186/s13071-025-06772-5 (PMC11963550; doi:10.1186/s13071-025-06772-5)
Supplement: Supplementary file 6 — Additional file 6: Table S3. List of abbreviations and gene names in the heatmap comparing the differential expression of immune-related pathways genes. [file 13071_2025_6772_MOESM6_ESM.pdf]

Additional file 6：Table S3 List of abbreviation and gene name in the heatmap comparing the differential expression of immune-related pathways genes

Pathway	Gene Number	Abbreviation of Gene Name	Gene Name	TPM (C_D7_1)	TPM (C_D7_2)	TPM (R_D7_1)	TPM (C_D7_2)	Mean (C_D7)	Mean (R_D7)	Fold change (R_D7/C_D7)
STAT	ASTE011642	STAT-A	signal transducer and transcription activator	29.13	27.49	34.34	30.01	28.31	32.175	1.14
JNK	ASTE007552	Jun	c-Jun N-terminal kinase	0.9	1.01	1.88	0.49	0.955	1.185	1.24
	ASTE005706	MAP2K	Mitogen-activated protein kinase kinase	31.64	26.02	29.47	25.59	28.83	27.53	0.95
	ASTE009465	MKK4	mitogen-activated protein kinase kinase 4	31.29	31.85	33.35	29.48	31.57	31.415	1.00
	ASTE001480	MAPK1	mitogen-activated protein kinase 1	37.2	39.59	36.11	33.98	38.395	35.045	0.91
	ASTE009854	MKK7	mitogen-activated protein kinase kinase 7	33.16	35.52	37.66	33.71	34.34	35.685	1.04
	ASTE008972	AP-1	Activating protein-1	29.83	32.87	38.6	28.75	31.35	33.675	1.07
	ASTE000190	Rac2	Rac family small GTPase 2	19.83	20.47	21.9	14.93	20.15	18.415	0.91
	ASTE010709	Rac1	Rac family small GTPase 1	33.06	29.31	45.49	24.91	31.185	35.2	1.13
	ASTE002506	MAPK-p38b	p38b MAP kinase	0	1.5	0	0	0.75	0	0
PGRP	ASTE000822	PGRP	Peptidoglycan-recognition protein	13.5	11.83	49.3	284.82	12.665	167.06	13.19
IMD	ASTE000778	Caspar	Caspar	79.6	82.5	87.15	74.11	81.05	80.63	0.99
	ASTE011281	Defensin	Defensin	1041.9	1304.19	1923.51	4545.26	1173.045	3234.385	2.76
	ASTE008048	TRAF	TNF-receptor-associated factor	17.17	16.36	18.44	15.37	16.765	16.905	1.01
	ASTE001196	CLIPC6	serine protease persephone	47.33	50.98	46.53	45.51	49.155	46.02	0.94
	ASTE009496	PIK3R3	phosphoinositide-3-kinase, regulatory subunit	20.27	17.9	17.58	17.6	19.085	17.59	0.92
	ASTE001304	kayak (kay)	Transcription factor kayak	41.72	41.84	59.32	43.13	41.78	51.225	1.23
	ASTE016290	APL1	Anopheles Plasmodium-responsive leucine-rich repeat 1	95.06	132.58	231.27	279.53	113.82	255.4	2.24
	ASTE000988	FADD	Fas-Associated Death Domain	9.43	7.07	7.08	6.38	8.25	6.73	0.82
	ASTE010360	Rel2	NF-kappaB Relish-like transcription factor	26.28	28.27	34.42	33.66	27.275	34.04	1.25
GNBP	ASTE010371	GNBP	beta-1,3-glucan-binding protein	6.78	9.34	13.61	16.18	8.06	14.895	1.85
	ASTE016199	GNBPB1	beta-1,3-glucan-binding protein B1	46.56	59.27	84.78	96.05	52.915	90.415	1.71
Toll	ASTE016386	TLR1A	TOLL-like receptor 1A	29.12	30.5	25.97	27.96	29.81	26.965	0.90
	ASTE004892	NEMO	Nuclear factor kappa B essential modulator	67.13	75.65	79.02	74.8	71.39	76.91	1.08
	ASTE003293	Ankyrin	Ankyrin	1.51	2.39	2.27	2.06	1.95	2.165	1.11
	ASTE003061	Trypsin	Trypsin	9.24	10.23	10.58	8.24	9.735	9.41	0.97
	ASTE009923	Ankyrin3	Ankyrin	12.33	15.44	18.91	12.98	13.885	15.945	1.15
	ASTE011577	TRAF4	TNF Receptor-Associated Factor4	1.23	0.8	2.72	0.83	1.015	1.775	1.75
	ASTE005309	TOLLIP	Toll-interacting protein	61.68	70.3	64.72	64.92	65.99	64.82	0.98
	ASTE004928	TLR11	Toll-like receptor 11	1.84	1.81	2.86	2.06	1.825	2.46	1.35
	ASTE004926	TLR10	Toll-like receptor 10	0.62	0.75	1.38	0.54	0.685	0.96	1.40
	ASTE000234	TLR7	Toll-like receptor 7	0.47	0.59	0.77	0.49	0.53	0.63	1.19

	ASTE004591	TLR6	Toll-like receptor 6	0.17	0.15	0.41	0.11	0.16	0.26	1.625
	ASTE010442	TLR9	Toll-like receptor 9	10.16	11.99	16.77	11.75	11.075	14.26	1.29
	ASTE011378	Rel1	NF-kappaB Relish-like transcription factor	52.48	57.99	57.75	51.5	55.235	54.625	0.99
TEP	ASTE016444	TEP1	thioester-containing protein 1	45.54	66.27	123.17	154.85	55.905	139.01	2.49
	ASTE016445	TEP3	thioester-containing protein 3	12.05	20.44	32.82	39.97	16.245	36.395	2.24
	ASTE008182	TEP4	thioester-containing protein 4	5.67	6.95	16.69	29.8	6.31	23.245	3.68
PPO	ASTE004215	PPO1	prophenoloxidase 1	0	0.05	0.07	0.03	0.025	0.05	2
	ASTE016298	PPO2	prophenoloxidase 2	13.12	17.02	41.3	29.12	15.07	35.21	2.34
	ASTE016298	PPO3	prophenoloxidase 3	1.22	1.19	9.45	5.41	1.205	7.43	6.17
	ASTE016300	PPO9	prophenoloxidase 9	2.83	5.24	8.26	2.73	4.035	5.495	1.36
