# Supplementary material for: Thioester-containing protein TEP15 promotes malaria parasite development in mosquitoes through negative regulation of melanization
Source: Parasit Vectors. 2025 Apr 1;18:124. doi: 10.1186/s13071-025-06772-5 (PMC11963550; doi:10.1186/s13071-025-06772-5)
Supplement: Supplementary file 7 — Additional file 7: Fig. S4. The expression levels of genes discovered in transcriptome sequencing were further confirmed by quantitative PCR experiments. (a) Detection of changes in the expression of AsAPL1 after AsTEP15 knockdown. The mRNA levels of APL1 in P. yoelii-parasite-infected mosquitoes (n = 15) were determined by using real-time PCR following AsTEP15 knockdown at day 7 PI. (b–d) Detection of immune-related signal pathway expression changes after AsTEP15 knockdown. The mRNA levels of JNK, Rel1, and STAT-A in P. yoelii-parasite-infected mosquitoes (n = 15) determined using real-time PCR following AsTEP15 knockdown on day 7 PI. [file 13071_2025_6772_MOESM7_ESM.pdf]

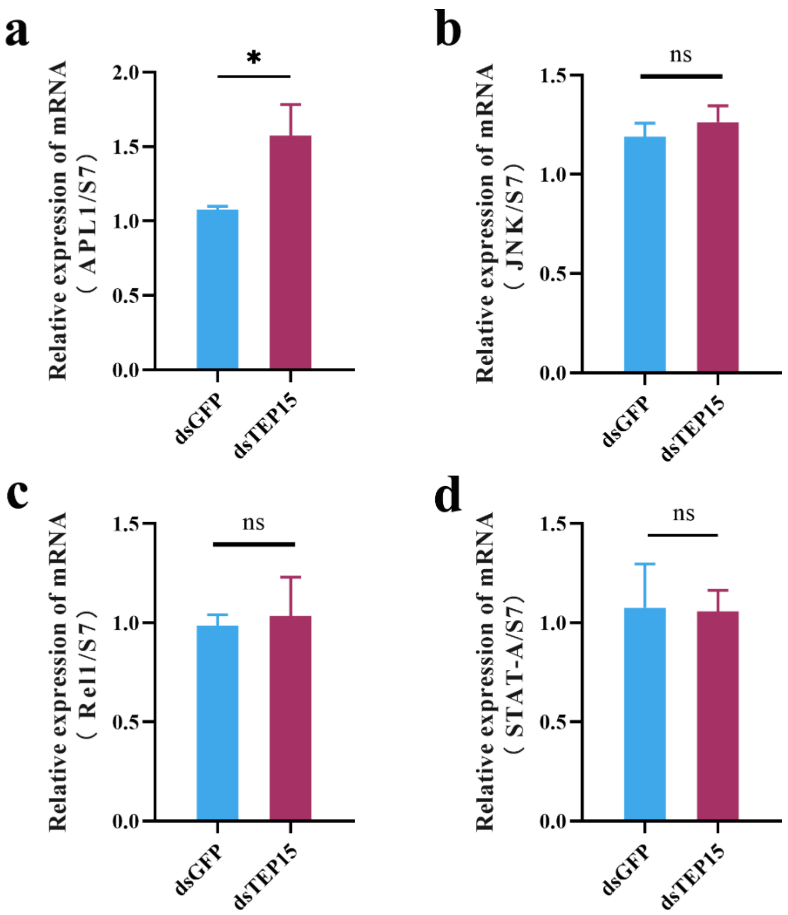

Additional file 7: Figure S4. The expression levels of genes discovered in transcriptome sequencing were further confirmed by quantitative PCR experiments. (**a**) Detection of changes in the expression of AsAPL1 after AsTEP15 knockdown. The mRNA levels of APL1 in *P. yoelii* parasite-infected mosquitoes (n=15) were determined by using real-time PCR following AsTEP15 knockdown at day 7 PI. **b-d** Detection of immune-related signal pathways expression changes after AsTEP15 knockdown. The mRNA levels of JNK **(b)**, Rel1 **(c)** and STAT-A **(d)** in *P. yoelii* parasite-infected mosquitoes (n=15) determined by using real-time PCR following AsTEP15 knockdown at day 7 PI.
