# Supplementary material for: Thioester-containing protein TEP15 promotes malaria parasite development in mosquitoes through negative regulation of melanization
Source: Parasit Vectors. 2025 Apr 1;18:124. doi: 10.1186/s13071-025-06772-5 (PMC11963550; doi:10.1186/s13071-025-06772-5)
Supplement: Supplementary file 8 — Additional file 8: Fig. S5. Detection of AsTEP1 expression change after AsTEP15 and AsRel2 double knockdown. [file 13071_2025_6772_MOESM8_ESM.pdf]

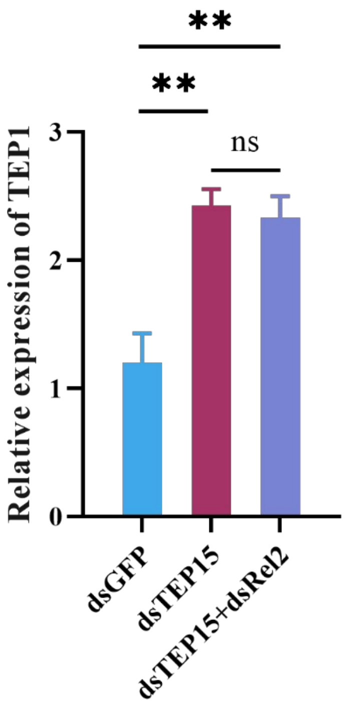

Additional file 8: Figure S5 Detection of AsTEP1 expression change after AsTEP15 and AsRel2 double knockdown.
